# Chiral Copper Catalysis in Enantioselective Domino Reactions

**DOI:** 10.3390/molecules30173654

**Published:** 2025-09-08

**Authors:** Hélène Pellissier

**Affiliations:** Aix Marseille Univ., CNRS, Centrale Med, iSm2, Marseille, France; h.pellissier@univ-amu.fr

**Keywords:** asymmetric copper catalysis, enantioselective domino reactions, metal catalysis, chirality, asymmetric catalysis, green chemistry

## Abstract

This review updates the field of enantioselective copper-catalysed domino reactions promoted by chiral green copper catalysts, covering the literature since 2017. These complexes are derived from a diversity of chiral ligands, including mostly bisoxazolines and biphosphines along with monophosphines, *N-*heterocyclic carbenes, proline derivatives, phosphoric acids, phosphoramidates, and different types of *N*,*N*-ligands. The review shows that asymmetric copper catalysis, that suits the growing demand for greener processes, offers a real opportunity to replace toxic and expensive metals in the near future.

## 1. Introduction

Combining asymmetric metal catalysis [1,2,3,4,5,6,7,8,9,10,11,12,13,14,15] with the concept of domino reactions [16,17,18,19,20,21,22,23,24,25,26,27] has allowed the easy synthesis of many types of chiral complex products to be simply synthesised with generally remarkable enantioselectivities. In comparison with other transition metals, copper presents the advantages of being not toxic, abundant, and cheap, which has resulted in its wide application to promote a diversity of greener reactions [28,29,30], among which are many types of enantioselective domino reactions [31]. Indeed, in the last decade the blooming of these reactions has permitted the synthesis of a wide diversity of cyclic as well as acyclic complex chiral molecules. The chiral copper catalysts employed in these reactions were prepared from a diversity of chiral ligands, including mostly bisoxazolines and biphosphines along with monophosphines, *N-*heterocyclic carbenes, proline derivatives, phosphoric acids, phosphoramidates, and different types of *N*,*N*-ligands. The goal of this review is to update the field of enantioselective copper-catalysed domino reactions since 2017 as this field was most recently reviewed in 2018, including references ≤2016 [31]. It must be noted that a more general review entitled “Recent Developments in Enantioselective Domino Reactions. Part B: First Row Metal Catalysts” was published in 2023 but it included only nine references ≥2017 dealing with copper as metal catalyst [27]. Moreover in 2025, Papis et al. published a review almost completely dedicated to racemic copper-catalysed multicomponent reactions, including no reference of asymmetric reactions ≥2017 [32]. The present review is divided into two parts, dealing successively with enantioselective copper-catalysed one- and two-component domino reactions, and multicomponent domino processes. The first part includes asymmetric Michael-initiated reactions, Kinugasa-initiated reactions, borylcupration-initiated reactions, reactions based on intramolecular cyclisation, reactions initiated by decarboxylative propargylation, Mannich-initiated reactions, arylation-initiated reactions, domino yne–allylic substitution/cyclisation reactions, and miscellaneous domino reactions. The second part of the review incorporates Michael-initiated three-component reactions, photoredox-catalysed three-component reactions, multicomponent reactions initiated by hydrocupration, and miscellaneous asymmetric multicomponent reactions.

## 2. One- and Two-Component Domino Reactions

### 2.1. Michael-Type-Initiated Domino Reactions

**Scheme 1:**
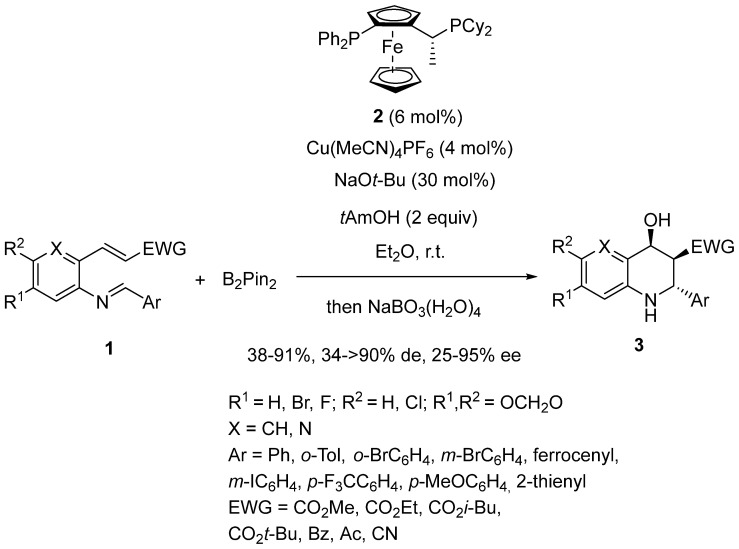
Domino conjugate borylation/Mannich reaction of Michael acceptors with B_2_Pin_2_ in the presence of a biphosphine ligand [34].

A range of copper-catalysed Michael-type additions have been employed to initiate many types of enantioselective domino reactions [33]. As an example, Lautens et al. described in 2020 a novel diastereo- and enantioselective copper-catalysed conjugate borylation/Mannich cyclisation reaction of Michael acceptors **1** with B_2_Pin_2_ (Scheme 1) [34]. It involved a catalyst system in situ generated from 4 mol% of Cu(MeCN)_4_PF_6_ and 6 mol% of chiral ferrocenyl biphosphine ligand **2**. The process was performed under mild conditions at room temperature in diethyl ether as the solvent in the presence of 30 mol% of NaO*t*-Bu as a base and two equivalents of bulky alcohol *t*AmOH as a super stoichiometric additive. After subsequent oxidation by treatment with NaBO_3_‧4H_2_O, it afforded a range of chiral tetrahydroquinolines **3** bearing three contiguous stereocentres with moderate to high yields (38–91%), diastereo- (34- > 90% de), and enantioselectivities (25–95% ee).

**Scheme 2:**
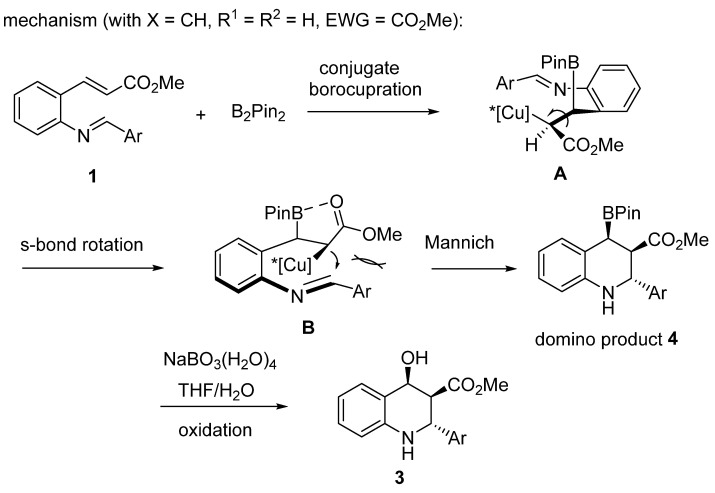
Mechanism for domino conjugate borylation/Mannich reaction of Michael acceptors with B_2_Pin_2_ in the presence of a biphosphine ligand [34].

As illustrated in Scheme 2, the reaction began by the *syn*-addition of the boronate and copper on the same face of the conjugated double bond to give intermediate **A**. Then, related to the oxophilic nature of boron, a rotation around the sigma bond occurred to generate five-membered intermediate **B** exhibiting the methyl ester group *cis* to the BPin group. To achieve the final Mannich cyclisation, the copper enolate added to the imine moiety through a half-chair transition state, allowing a lower interaction between the catalyst and the aryl group of the imine, which resulted in the formation of chiral borylated tetrahydroquinoline **4**. The latter was subsequently oxidised into the corresponding chiral hydroxy ester **3** with complete retention of enantio- and diastereoselectivities. The catalyst system tolerated various aryl groups (Ar), leading to the corresponding products with both high de, and ee values with the best results obtained in the case of electron-rich imines. Thiophenyl- and ferrocenyl-containing substrates (Ar = 2-thienyl, ferrocenyl) also reacted with high enantio- (82–96% ee) and diastereoselectivities (84- > 90% de). Substituents on the aryl backbone (R^1^ = Br, F; R^2^ = Cl) were compatible since the desired products were obtained with 70–85% yields, 84- > 90% de, and 63–90% ee. Studying the nature of the Michael acceptor, the authors found lower diastereo- and enantioselectivities upon increasing the size of the ester group (EWG = CO_2_Me: >90% de, and 89% ee; EWG = CO_2_Et: 90% de, and 86% ee; EWG = CO_2_*i*-Bu: 78% de, and 80% ee; EWG = CO_2_*t*-Bu: 34% de, and 68% ee). Along with esters, ketones (EWG = Bz, Ac) also reacted to give the corresponding tetrahydroquinolines with 74–78% yields, 60–70% de, and 63–95% ee. Moreover, an excellent ee value (94% ee) was obtained in the reaction of a nitrile (EWG = CN) while combined with moderate yield (56%) and de value (42% de). It must be noted that this work represented a rare example of copper-catalysed borylation of an 1,4-unsaturated Michael system with subsequent cyclisation, and also the first to involve an imine. Considering the importance of chiral trisubstituted tetrahydroquinolines in medicinal chemistry as well as the limited synthetic methods for their preparation, this novel simple methodology constituted an important advance.

**Scheme 3:**
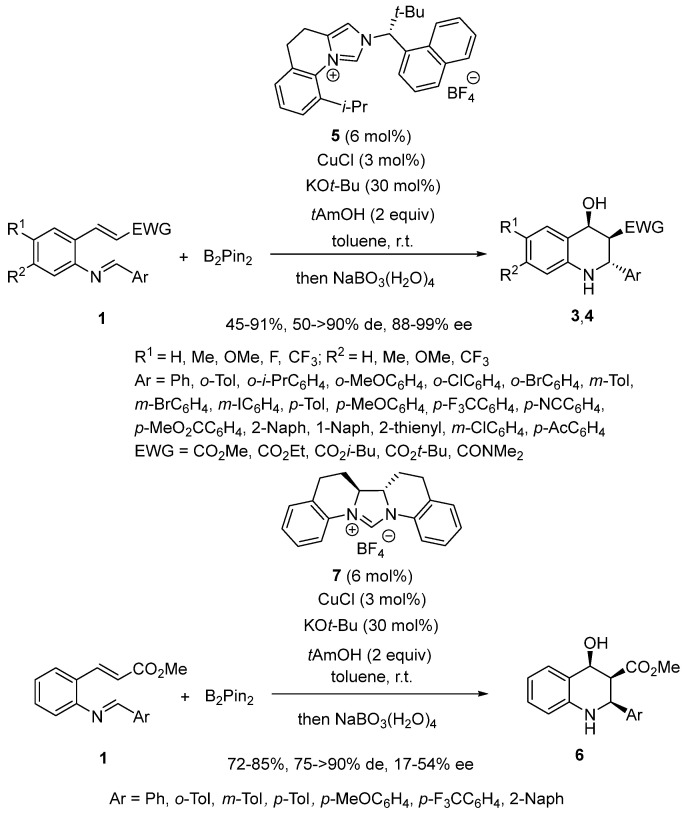
Domino conjugate borylation/Mannich reactions of Michael acceptors with B_2_Pin_2_ in the presence of NHC ligands [35].

Later in 2023, Fan et al. reinvestigated these reactions by using another type of chiral copper ligands, such as *N-*heterocyclic carbenes (NHCs) [35]. Using novel NHC ligand **5** (6 mol%) combined with CuCl (3 mol%) as a precatalyst in toluene at room temperature in the presence of *t*AmOH as a super stoichiometric additive and KO*t*-Bu as a base allowed an impressively broad variety of chiral 2,3-*trans*-3,4-*cis*-trisubstituted hydroxylated tetrahydroquinolines **3**,**4** to be achieved after further oxidation with good to high yields (45–91%), uniformly excellent ee values (88–99% ee), combined with generally high de values (50- > 90% de), as shown in Scheme 3. This demonstrated that this class of ligands showed better performance than chiral biphosphine **2**. Notably, even an α,β-unsaturated amide (EWG = CONMe_2_) could be involved as a Michael acceptor, leading to the corresponding product with both excellent diastereo- (>90% de) and enantioselectivities (95% ee). Investigating other NHC ligands in these reactions, the authors found that the steric hindrance of the ligand played an important role in determining the diastereoselectivity of the reaction. Indeed, thermodynamically challenging all-*cis* chiral 2,3,4-trisubstituted hydroxylated tetrahydroquinolines **6** could be generated by using a less bulky C_2_-symmetric NHC ligand, such as **7**.

**Scheme 4:**
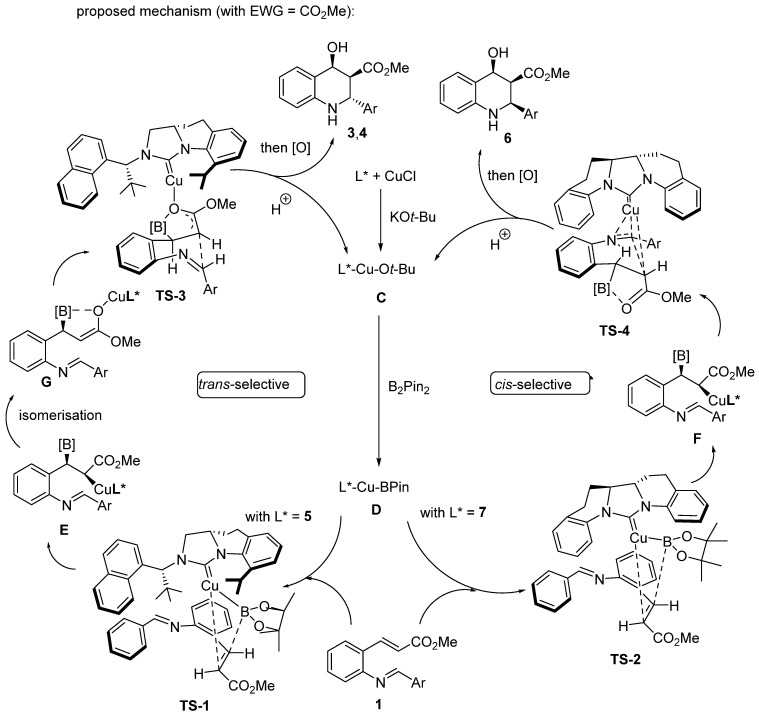
Mechanism for domino conjugate borylation/Mannich reactions of Michael acceptors with B_2_Pin_2_ in the presence of NHC ligands [35].

As illustrated in Scheme 4, the employment of a chiral copper catalyst in situ prepared from 3 mol% of CuCl and 6 mol% of chiral NHC ligand **7** resulted in the formation of a series of all-*cis* chiral 2,3,4-trisubstituted tetrahydroquinolines **6** with both good to high yields (72–85%) and diastereoselectivities (75- > 90% de), albeit combined with low to moderate enantioselectivities (17–54% ee) after oxidation. To explain the diastereoselectivity of the domino reaction, two catalytic cycles are proposed in Scheme 4 for the two types of chiral NHC ligands **5** and less sterically hindered **7**. They both began with a *syn*-1,2-borocupration of the Michael acceptor by boryl-copper species **D**, evolving through respective transition states **TS**-**1** and **TS**-**2,** which led to chiral intermediates **E** and **F**, respectively. Subsequently, intermediate **D** underwent a reversible isomerisation to a more stable cyclic enolate intermediate **G** due to the steric hindrance of ligand **5**. Finally, 2,3-*trans* products **3**,**4** were produced via transition state **TS**-**3**. In contrast, in the case of using the less bulky ligand **7**, intermediate **F** was directly converted into the all-*cis* product **6** through transition state **TS**-**4**. In this case, the diastereoselectivity arose from the steric interaction between the Ar group of the imine and the bulky Cu-NHC catalyst.

**Scheme 5:**
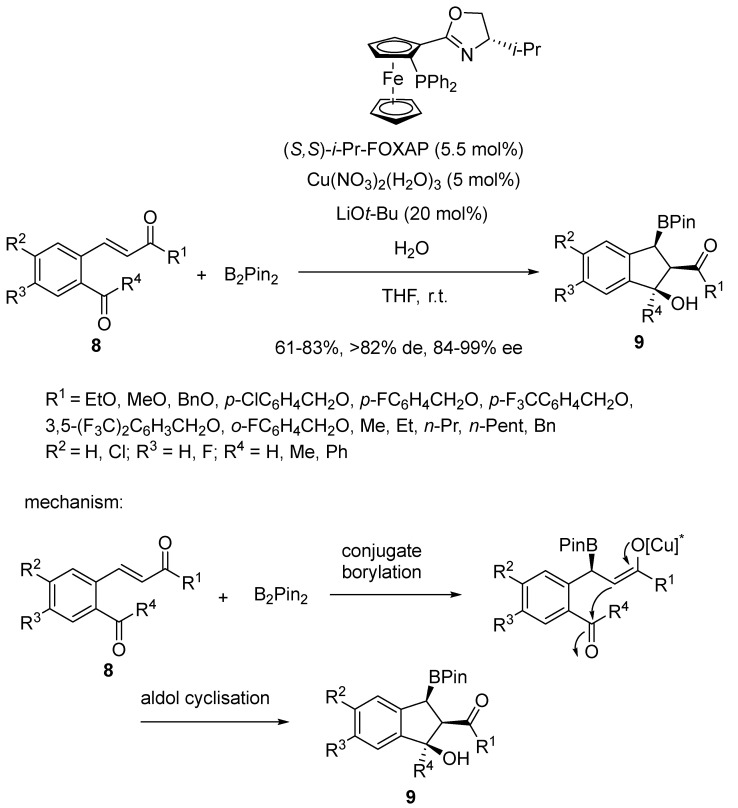
Domino conjugate borylation/aldol reaction of β-(*o*-acylphenyl)-substituted electron-deficient alkenes with B_2_Pin_2_ [36].

Earlier in 2020, Zhang and Li developed asymmetric domino conjugate borylation/aldol reactions, which involved β-(*o*-acylphenyl)-substituted electron-deficient alkenes **8** and B_2_Pin_2_ as the substrates (Scheme 5) [36]. A chiral copper catalyst in situ prepared from 5 mol% of Cu(NO_3_)_2_(H_2_O)_3_ and 5.5 mol% of (*S*,*S*)-*i*-Pr-FOXAP as chiral phosphine ligand was employed to promote the reaction at room temperature in THF as solvent in the presence of water as an additive and LiO*t*-BuO as a base, which resulted in the formation of chiral indanes **9** exhibiting three consecutive stereogenic centres with good to high yields (61–83%) and uniformly high enantioselectivities (84–99% ee). In all cases, almost single diastereomers were obtained (>82% de). The catalyst system tolerated a variety of ester groups, spanning from simple alkyl esters (R^1^ = OMe, OEt) to differently substituted benzyl groups, delivering the corresponding indane derivatives with 62–76% yields and 84–99% ee. Along with acrylate substrates, α,β-unsaturated ketones (R^1^ = alkyl) reacted smoothly to produce the desired products with 63–83% yields and 90–99% ee. Moreover, the aldehyde group at the *ortho* position of the phenyl ring (R^4^ = H) could be changed into a methyl or a phenyl group (R^4^ = Me, Ph), which allowed 3-(2-acylphenyl)acrylates and β-(2-acylaryl)enones bearing a tertiary alcohol to be achieved in good yields (62–75%) and excellent enantioselectivities (90–99% ee). The advantages of this novel simple methodology relied on its good yields, excellent chemo-, regio-, diastereo-, and enantioselectivities, broad functional group tolerance, and its cheap catalyst.

**Scheme 6:**
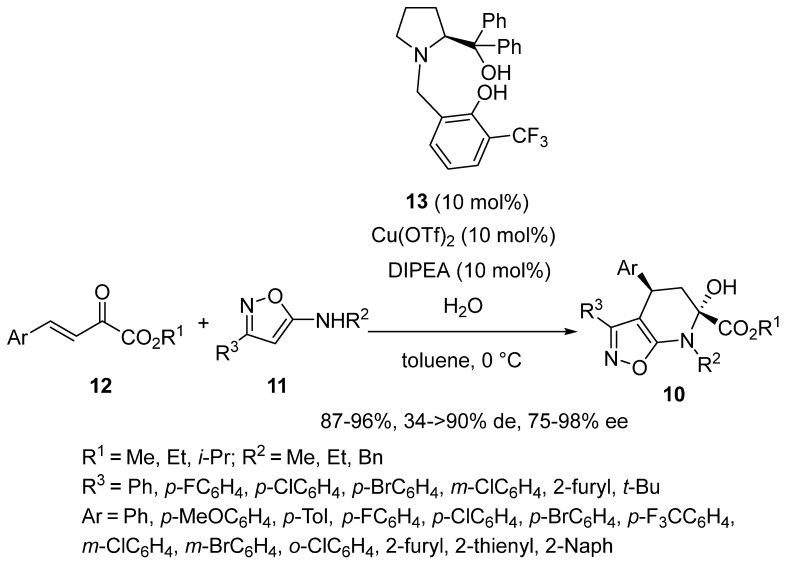
Domino Michael/*N-*hemiacetalisation reaction of 5-aminoisoxazoles with β,γ-unsaturated α-ketoesters [37].

Isoxazole derivatives constitute key molecules in medicinal chemistry and, consequently, extensive effort has been devoted to their synthesis. In 2023, Wang et al. developed a novel route to chiral isoxazole-derived six-membered *N*,*O*-hemiaminals **10** arisen from an enantioselective domino Michael/*N*-hemiacetalisation reaction of 5-aminoisoxazoles **11** with β,γ-unsaturated α-ketoesters **12** (Scheme 6) [37]. This one-pot process was catalysed in toluene at 0 °C by a combination of 10 mol% of Cu(OTf)_2_ and the same quantity of chiral proline-derived diol **13** as a ligand. In the presence of DIPEA as a base and water as an additive, a range of chiral *N*,*O*-hemiaminals **10** were produced with high yields (87–96%), moderate to excellent diastereoselectivities (34- > 90% de), and good to very high ee values (75–98% ee). Both the two substrates tolerated different types of substituents. Surprisingly, along with 3-aryl-5-aminoisoxazoles (R^3^ = aryl), a 5-aminoisoxazole exhibiting a *tert-*butyl group on the isoxazole part (R^3^ = *t*-Bu) also led to the corresponding product with 96% yield and 98% ee.

### 2.2. Kinugasa-Initiated Domino Reactions

**Scheme 7:**
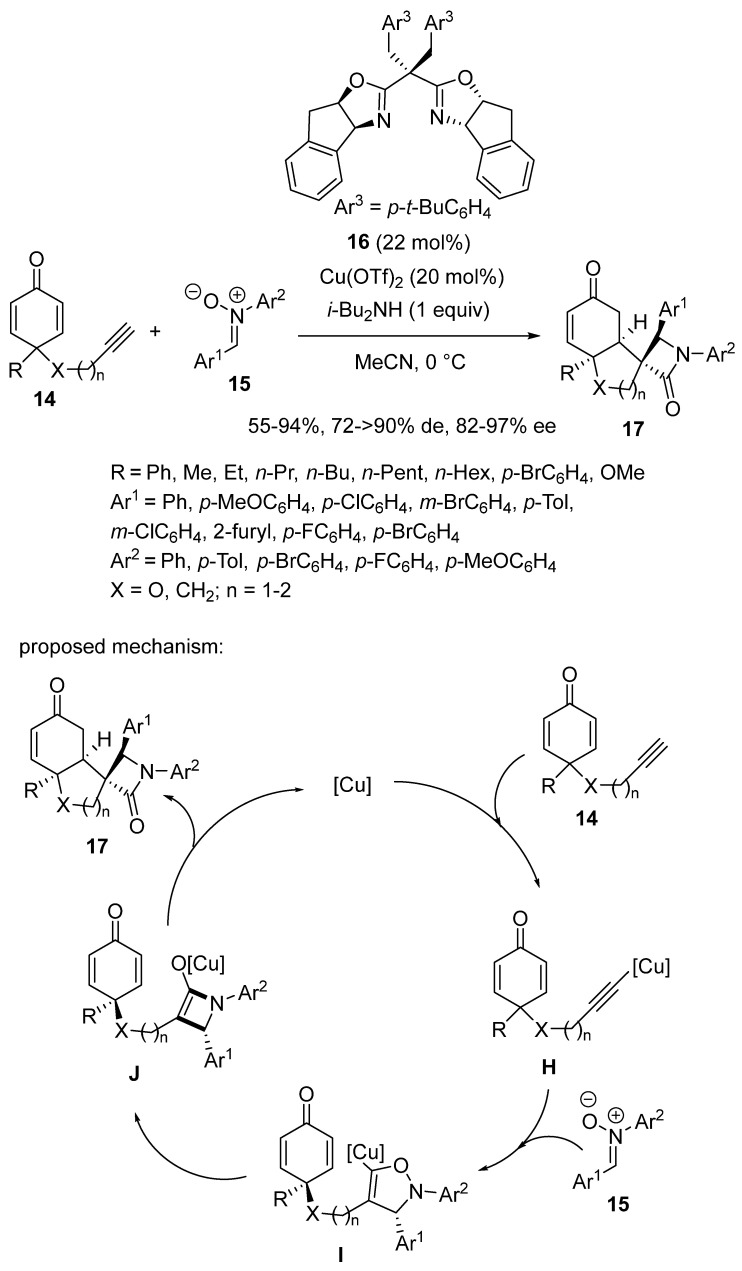
Domino Kinugasa/Michael reaction of alkyne-tethered cyclohexadienones with nitrones [40].

The Kinugasa reaction allows a direct access to β-lactams from terminal alkynes and nitrones [38,39]. In spite of its utility, this reaction has been rarely included in domino sequences. To fill this gap, Enders et al. reported in 2018 the first example of enantioselective copper-catalysed domino Kinugasa/Michael reaction between alkyne-tethered cyclohexadienones **14** and nitrones **15** (Scheme 7) [40]. It was promoted at 0 °C in acetonitrile as the solvent by a chiral copper catalyst in situ generated from 20 mol% of Cu(OTf)_2_ and 22 mol% of chiral indane-bisoxazoline ligand **16** in the presence of *i*-Bu_2_NH as a base. This resulted in the unprecedented regio-, chemo-, diastereo-, and enantioselective formation of a range of chiral spirocyclic β-lactams **17** exhibiting a fused bicyclic and spiro-fused bicyclic framework with four contiguous stereocentres with good to high yields (55–94%). In almost all cases of substrates, the complex and densely functionalised products were achieved as almost single diastereomers (>90% de) with high enantioselectivities (82–97% ee). Indeed, rare lower diastereoselectivities (72–83% de) were observed in the reaction of cyclohexadienones bearing longer alkyl chains (R = *n*-Bu, *n*-Pent, *n*-Hex) albeit combined with excellent ee values (94–95% ee). To explain the results, the authors proposed the mechanism depicted in Scheme 7, beginning with the reaction between the Cu(II) catalyst and the alkyne-tethered cyclohexadienone to give copper acetylide intermediate **H**. Subsequently, the latter underwent [3 + 2] cycloaddition with the nitrone to form the copper-bound isoxazoline intermediate **I**. Then, the latter species underwent rearrangement into tethered four-membered copper enolate intermediate **J**, which was further submitted to Michael addition to afford the final spirocyclic β-lactam along with regenerated copper catalyst.

**Scheme 8:**
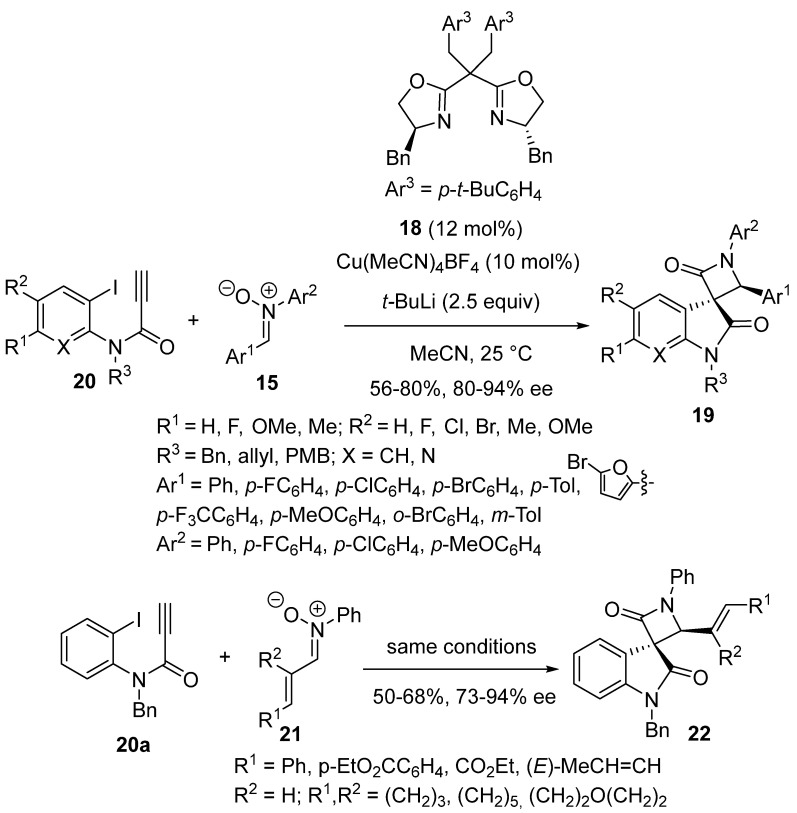
Domino Kinugasa/C—C coupling reactions of *N*-(2-iodo-aryl)-propiolamides with nitrones [41].

Among bioactive products are complex spiro[azetidine-indolines]. While the synthesis of chiral spiro[azetidine-2,3′-indolines] has been developed, that of chiral spiro[azetidine-3,3′-indolines] remains rarely investigated. To fill this gap, Cai and Zhou reported in 2022 the first copper-catalysed asymmetric domino Kinugasa/aryl C—C coupling reaction which allowed the synthesis of these molecules (Scheme 8) [41].This involved at 25 °C a mixture of 10 mol% of Cu(MeCN)_4_BF_4_ as precatalyst and 12 mol% of chiral bisoxazoline ligand **18** in acetonitrile as the solvent. Indeed, densely functionalised chiral spiro[azetidine-3,3′-indoline]-2,2′-diones **19** were produced as single diastereomers with 56–80% yields and 80–94% ee from the reaction of the corresponding *N*-(2-iodo-aryl)-propiolamides **20** with diaryl nitrones **15**. Studying the substrate scope, it was found that several protecting groups were tolerated on the amide nitrogen (R^3^), as well as various electron-donating and electron-withdrawing substituents on the phenyl ring of the *N*-(2-iodo-aryl)-propiolamides. Moreover, diarylnitrones could bear either electron-deficient or electron-rich substituents. The scope of the methodology was extended to α,β-unsaturated nitrones **21,** which were proven to smoothly react with *N*-(2-iodo-phenyl)-propiolamide **20a** to afford the corresponding chiral spiro[azetine-3,3′-indoline]-2,2′-diones **22** bearing alkene moieties with 50–68% yields and 73–94% ee as single diastereomers (Scheme 8). Conjugated ester (R^1^ = CO_2_Et) and diene (R^1^ = (*E*)-MeCH=CH) were compatible, leading to the desired products with 50–56% yields and 89–90% ee.

**Scheme 9:**
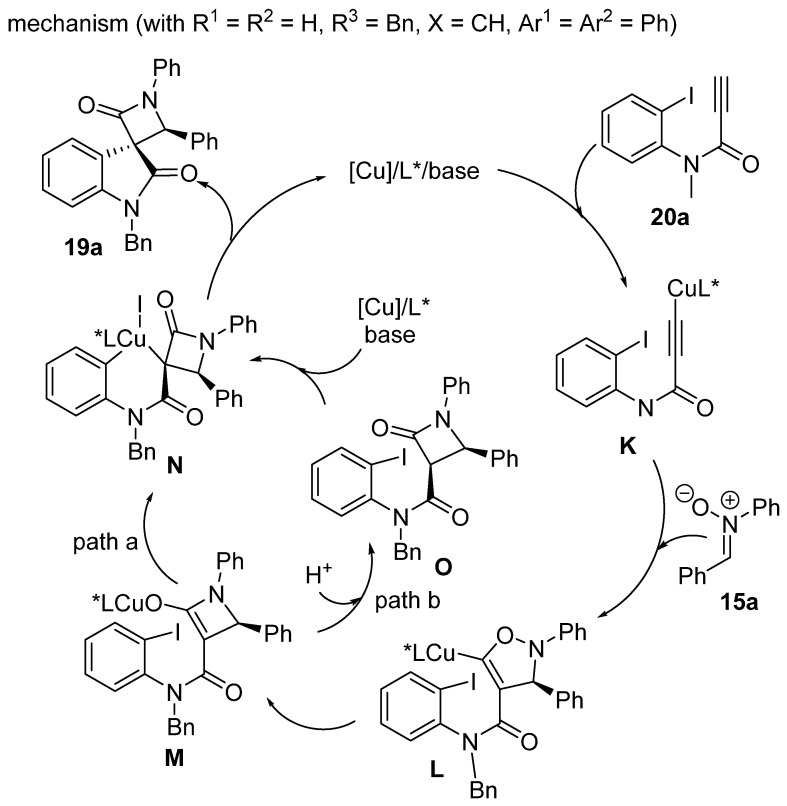
Mechanism for domino Kinugasa/C—C coupling reactions of *N*-(2-iodo-aryl)-propiolamides with nitrones [41].

Two pathways a and b depicted in Scheme 9 have been proposed by the authors to explain the results. The first one involved a direct trapping of the in situ generated copper intermediate **M** in the Kinugasa reaction for the aryl C—C coupling, while the second one evolved through a copper-catalysed C—C coupling of intermediate **N**, arising from protolysis of **M** under basic conditions.

**Scheme 10:**
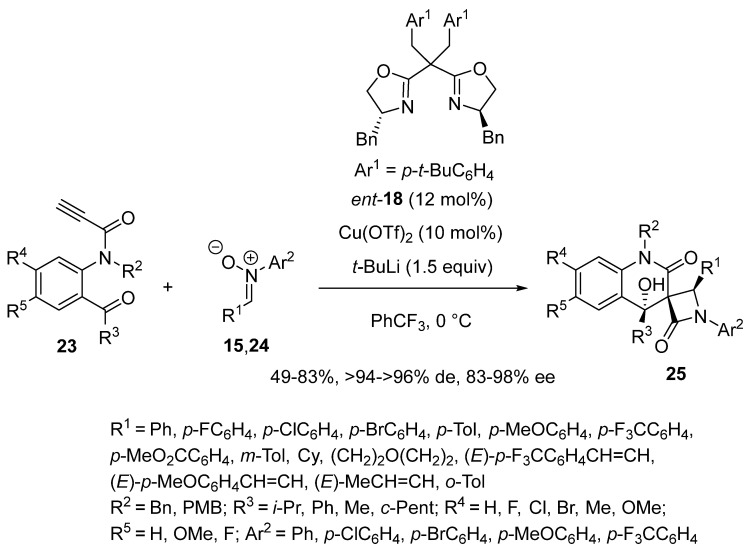
Domino Kinugasa/aldol reaction of ketone-tethered propiolamides with nitrones [42].

Later in 2023, enantiomeric ligand *ent-***18** was applied by the same group to promote the first enantioselective copper-catalysed domino Kinugasa/aldol-type reaction between ketone-tethered propiolamides **23** and nitrones **15**,**24** (Scheme 10) [42]. The process was performed at 0 °C with 12 mol% of chiral oxazoline *ent-***18** combined with 10 mol% of Cu(OTf)_2_ in the presence of *t*-BuLi as a base in PhCF_3_ as the solvent, thus resulting in the formation of a diversity of novel chiral spiro β-lactams **25** exhibiting three contiguous stereogenic centres as single diastereomers (>94- > 96% de) with 49–83% yields and uniformly high ee values (83–98% ee). This constituted the first example of a cascade Kinugasa/aldol reaction involving ketones as the nucleophiles. The scope of the process was found broad since different types of substituents could be present on both the amide nitrogen and the *N*-tethered phenyl ring. Furthermore, both electron-withdrawing and electron-donating groups could be present on the two phenyl rings of diarylnitrones (R^1^ = aryl).

**Scheme 11:**
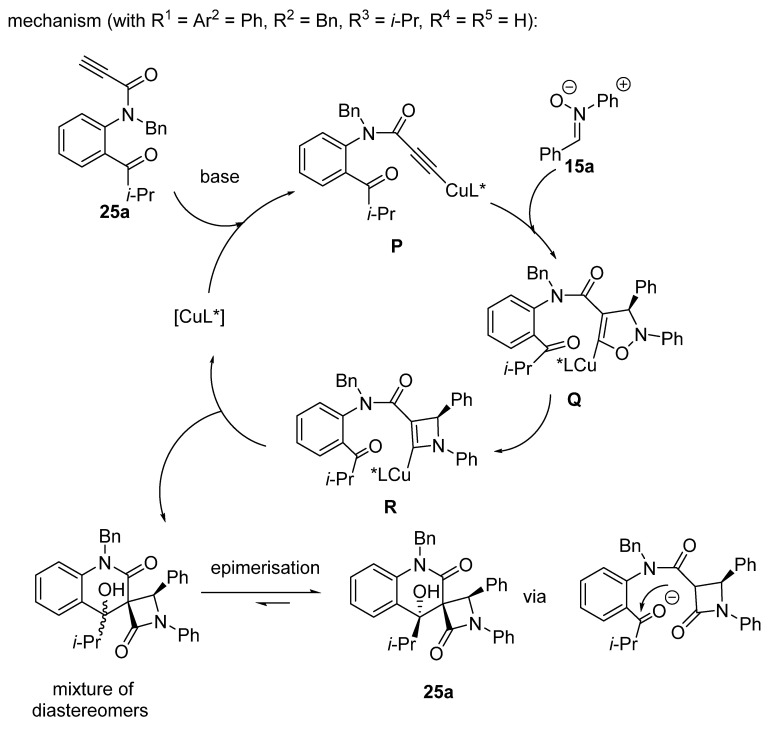
Mechanism for domino Kinugasa/aldol reaction of ketone-tethered propiolamides with nitrones [42].

Mechanistically, the authors proposed the catalytic cycle depicted in Scheme 11, beginning with the enantioselective copper-catalysed Kinugasa reaction, which afforded enolate intermediate **R**. Then, the latter underwent intramolecular aldol reaction with the carbonyl group to produce a diastereomeric mixture of spirobilactams. With the assistance of two electron-withdrawing amide groups, this mixture of diastereomers underwent a retro-aldol/aldol equilibrium process under basic conditions, which led to the formation of thermally more stable **25** as the almost single diastereomer.

**Scheme 12:**
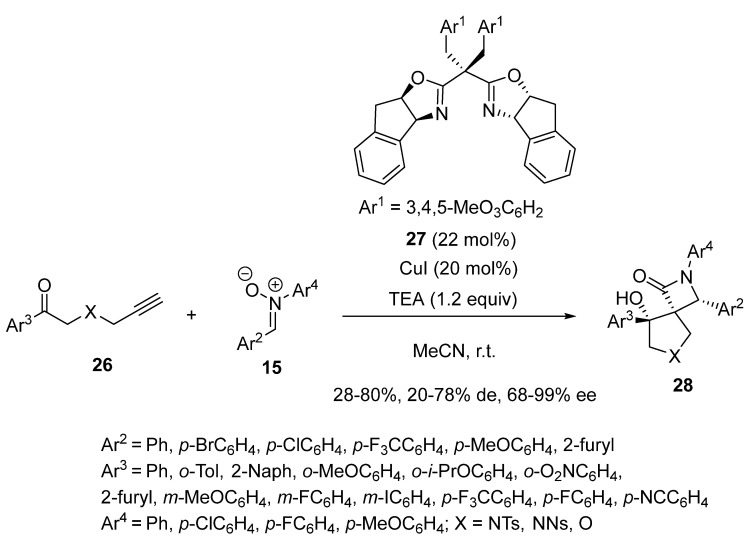
Domino Kinugasa/aldol reaction of alkyne-tethered ketones with diarylnitrones [43].

Another type of alkyne-tethered ketones, such as substrates **26**, was involved by Lautens et al. in comparable asymmetric domino Kinugasa/aldol reactions with diarylnitrones **15**, in 2023 [43]. In this case, 20 mol% of CuI was combined with 22 mol% of chiral bisoxazoline **27** to in situ generate the active catalyst system in acetonitrile as the solvent. The process was carried out at room temperature in the presence of TEA as a base, leading to chiral spirocyclic β-lactam pyrrolidinones **28** exhibiting three contiguous stereocentres with 28–80% yields, 20–78% de, and 68–99% ee, as illustrated in Scheme 12. Electron-withdrawing as well as electron-donating substituents could be present on the phenyl rings of the two substrates. Along with nitrogen-tethered (X = NTs, NNs) alkynes, an oxygen-tethered substrate (X = O) was also investigated, albeit giving the corresponding dihydrofuran product with the lowest yield (28%), as well as diastereo- (20% de), and enantioselectivities (68% ee).

**Scheme 13:**
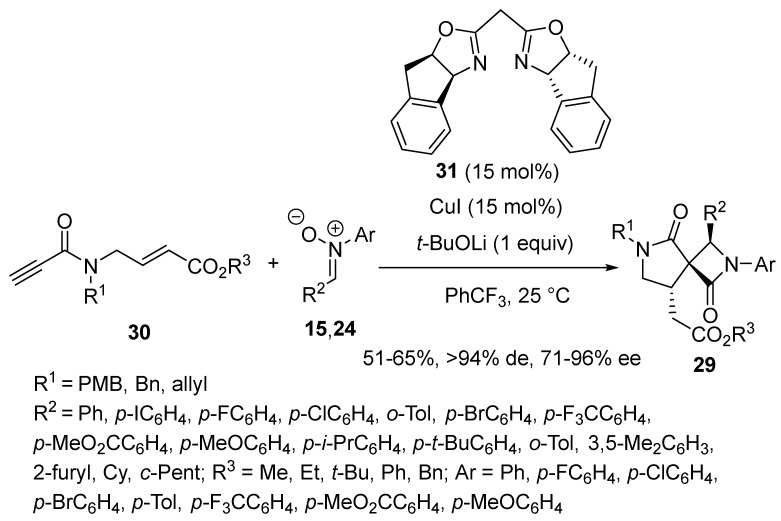
Domino Kinugasa/Michael reaction of α,β-unsaturated ester-tethered propiolamides with nitrones [44].

In 2024, Zhang and Cai described the synthesis of another type of functionalised chiral spiro β-lactams, such as 2,6-diazaspiro [3.4]octane-1,5-diones **29**, on the basis of an asymmetric domino Kinugasa/Michael reaction between α,β-unsaturated ester-tethered propiolamides **30** and nitrones **15**,**24** (Scheme 13) [44]. The process involved 15 mol% of CuI combined with the same quantity of chiral bisoxazoline ligand **31** as catalyst system in CF_3_Ph as solvent. Performed at 25 °C in the presence of LiO*t*Bu as base, it delivered a wide range of structurally diverse chiral spiro β-lactams **29** as almost single diastereomers (>94% de) with 51–65% yields and 71–96% ee. The α,β-unsaturated ester-tethered propiolamides tolerated different *N*-substituents as well as ester groups, providing the desired products with both high diastereo- and enantioselectivities. Furthermore, along with variously substituted diarylnitrones, C-alkyl nitrones (R^2^ = Cy, *c*-Pent) also reacted smoothly albeit with moderate enantioselectivities (71–76% ee). On the other hand, *N*-alkyl nitrones were not compatible.

### 2.3. Borylcupration-Initiated Domino Reactions

**Scheme 14:**
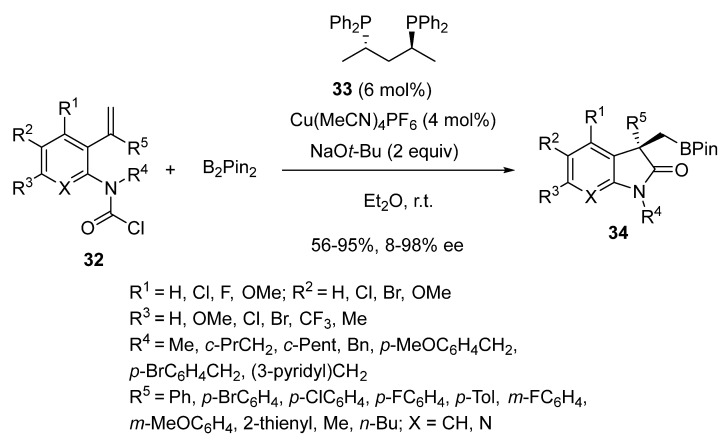
Domino borylcupration/acylation reaction of styrenes with B_2_Pin_2_ [45].

Recently, chiral copper catalysts have allowed asymmetric difunctionalisations of alkenes through domino reactions evolving through borylcupration followed by electrophilic trapping. As an example, enantioselective domino borylcupration/acylation reactions between functionalised styrenes **32** and B_2_Pin_2_ were developed by Lautens et al., in 2018 (Scheme 14) [45]. These processes were carried out in the presence of a base, such as NaO*t*-Bu, and a chiral catalyst in situ generated from 4 mol% of Cu(MeCN)_4_PF_6_ and 6 mol% of chiral biphosphine ligand **33** in diethyl ether as the solvent. After enantioselective borylcupration of the alkene moiety, a nucleophilic attack on the tethered carbamoyl chloride delivered a range of chiral borylated 3,3-disubstituted oxindoles **34** with 56–95% yields and 8–98% ee. Studying the substrate scope of the reaction, the authors found that varying the substituent on the nitrogen atom (R^4^) did not affect the level of enantioselectivity of the reaction (96–98% ee). In contrast, the presence of substituents at the *ortho* position (R^1^) of the styrene moiety dramatically affected the enantioselectivity of the process, since the corresponding products were obtained with 23–77% ee. Moreover, the nature of the pendant aromatic ring (R^5^) also influenced the stereoselectivity of the reaction (R^5^ = *p*-BrC_6_H_4_: 8% ee).

**Scheme 15:**
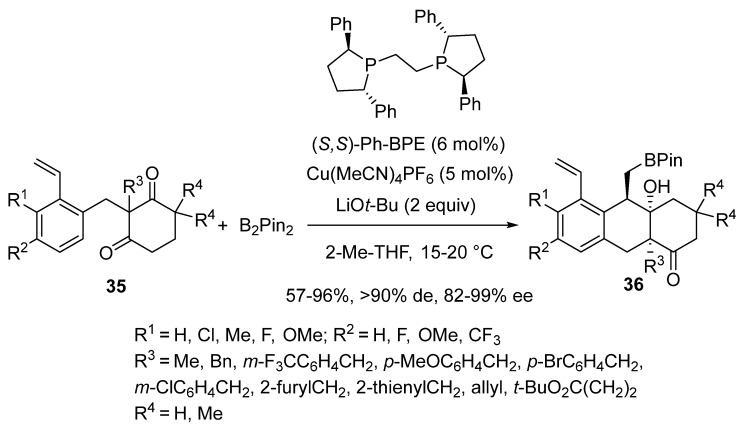
Domino borylcupration/addition reaction of styrenes with B_2_Pin_2_ [46].

In the same area, Xu and Zhao reported in 2019 a copper-catalysed desymmetrisation of 1,3-diketones involving borylation of styrenes [46]. As illustrated in Scheme 15, the domino borylcupration/addition reaction occurred between styrenes **35** and B_2_Pin_2_ in the presence of 5 mol% of Cu(MeCN)_4_PF_6_ combined with 6 mol% of chiral diphosphine ligand (*S*,*S*)-Ph-BPE in 2-Me-THF as solvent. Enantioselective borylcupration of the styrene moiety, followed by addition to the 1,3-diketone, provided access to a range of tricyclic functionalised chiral products **36** bearing three stereogenic centres including two quaternary ones as single diastereomers (>90% de) with 57–96% yields and uniformly high ee values (82–99% ee). Remarkably, a wide variety of substrates bearing different functional groups on the aryl skeletons, electron-neutral, electron-donating, as well as electron-withdrawing substituents on different positions, reacted smoothly to give the corresponding products in high yields and excellent enantioselectivities. Excellent results were also achieved with the presence of various substituents on the 1,3-diketone moiety.

**Scheme 16:**
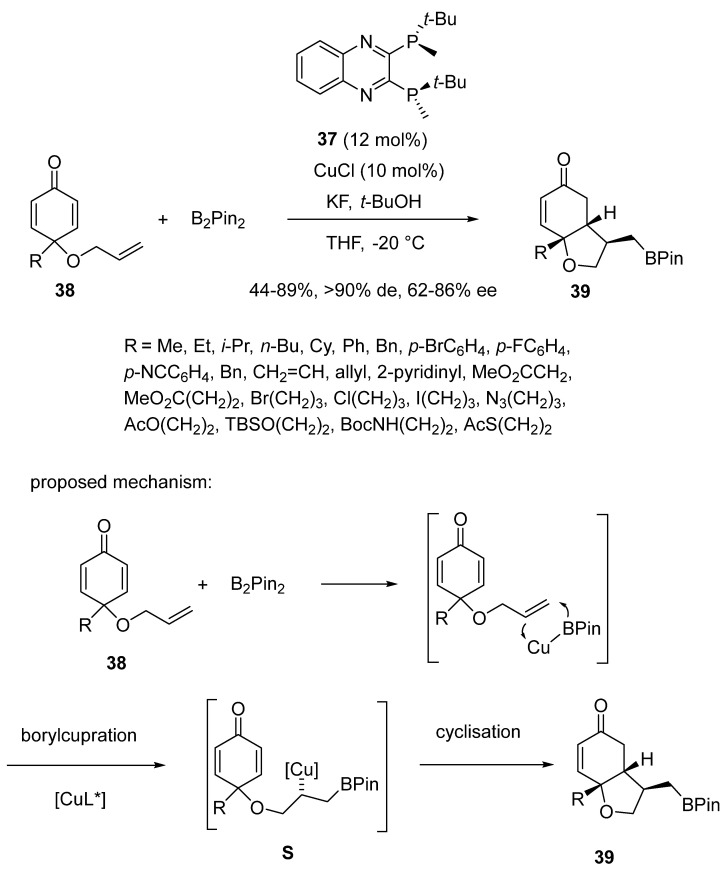
Domino borylcupration/cyclisation reaction of monosubstituted terminal alkene-tethered cyclohexadienones with B_2_Pin_2_ [47].

Another chiral biphosphine, such as **37** (12 mol%), was combined by Lin and Tian in 2020 to CuCl (10 mol%) as precatalyst to promote an enantioselective domino borylcupration/cyclisation reaction between alkene-tethered cyclohexadienones **38** and B_2_Pin_2_ (Scheme 16) [47]. Performed at room temperature in THF as the solvent, the reaction afforded chiral *cis-*fused bicyclic products **39** as single diastereomers (>90% de) in 44–89% yields and 62–86% ee. It involved regioselective anti-Markovnikov borylcupration of the tethered terminal alkene followed by cyclisation through conjugate addition of a stereospecific alkyl copper intermediate **S** onto the cyclohexadienone. The authors suggested that the 1,4-borylation of the electron-deficient double bond of the cyclohexadienone was avoided due to the presence of two substituents at the C4 position, which induced steric hindrance, and thus the tethered alkene was preferentially borylated.

### 2.4. Domino Reactions Based on Intramolecular Cyclisation

**Scheme 17:**
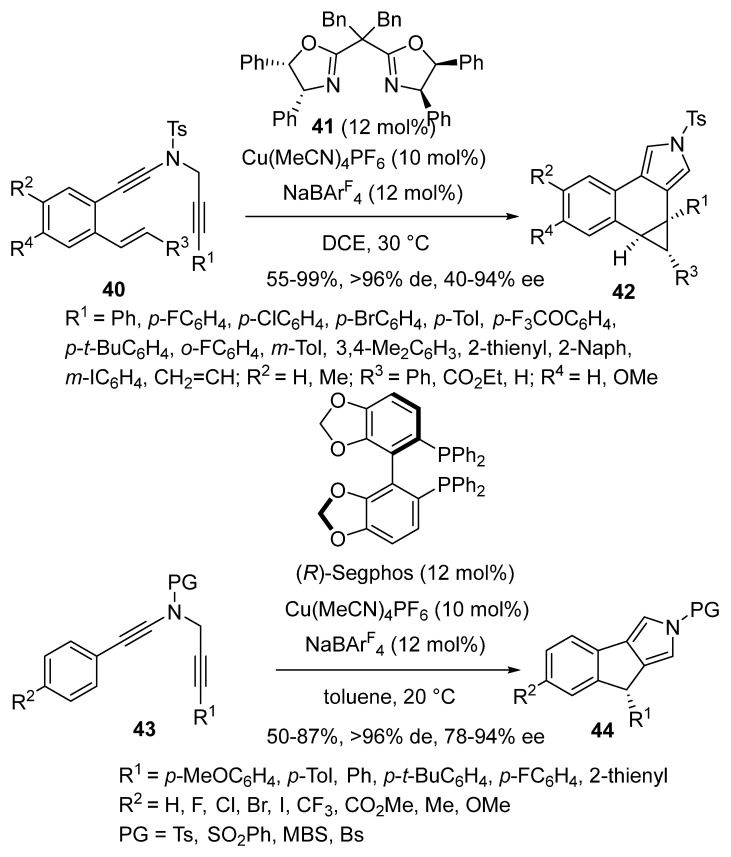
Domino cyclisation/cyclopropanation and cyclisation/C—H insertion reactions of (alkenyl) *N*-propargyl ynamides [48].

In 2019, Ye et al. reported enantioselective copper-catalysed domino reactions of *N*-propargyl ynamides which evolved through intramolecular cyclisation, allowing an easy access to different types of chiral polycyclic pyrroles [48]. In a first time, the domino cyclisation/cyclopropanation reaction of alkenyl *N*-propargyl ynamides **40** catalysed by a combination of 10 mol% of Cu(MeCN)_4_PF_6_ and 12 mol% of chiral bisoxazoline **41** in DCE at 30 °C resulted in the formation of the corresponding chiral tetracyclic pyrroles **42** as single diastereomers (>96% de) with 55–99% yields and 40–94% ee (Scheme 17). This result constituted the first example of enantioselective copper-catalysed diyne cyclisation. In a second time, the reaction of *N*-propargyl ynamides **43** was investigated in the presence of another type of chiral ligand, such as diphosphine (*R*)-Segphos. In this case, chiral tricyclic pyrroles **44** were formed as single diastereomers (>96% de) with 50–87% yields and 78–94% ee (Scheme 17). These molecules arose from a domino cyclisation/C—H insertion reaction performed in toluene at 20 °C. Mechanistic studies demonstrated the involvement of donor/donor copper carbenes.

**Scheme 18:**
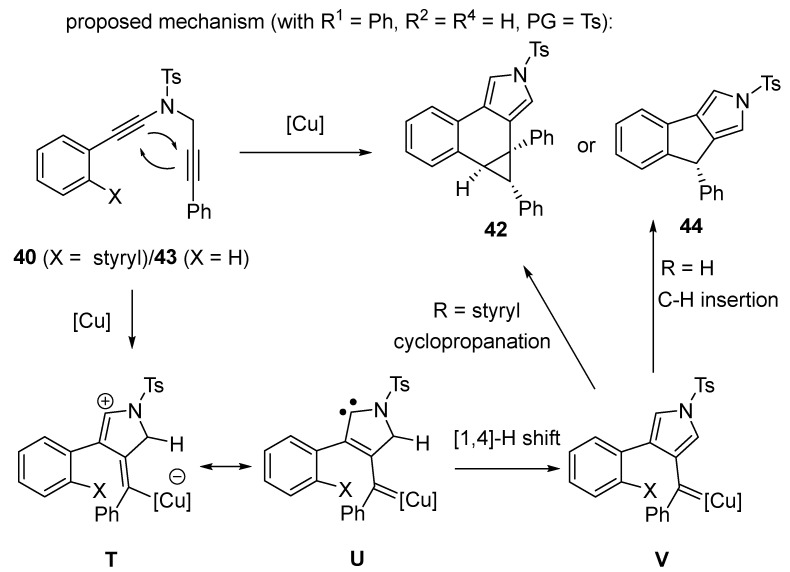
Mechanism for domino cyclisation/cyclopropanation and cyclisation/C—H insertion reactions of (alkenyl) *N*-propargyl ynamides [48].

A possible mechanism for the formation of the two types of products **42** and **44** is illustrated in Scheme 18. It began with the formation of the pyrrole vinyl copper intermediate **T** or its resonance form **U** through copper-catalysed cyclisation of the two alkyne moieties. Then, intermediate **U** underwent [1,4]-H shift, which generated donor/donor copper carbene intermediate **V**. Then, in the case of alkenyl *N*-propargyl ynamides **40**, an intramolecular cyclopropanation delivered the desired tetracyclic product **42**. Alternatively, in the case of *N*-propargyl ynamides **43**, donor/donor copper carbene intermediate **V** could be trapped by the aryl group via C−H insertion, yielding tricyclic pyrrole **44**.

**Scheme 19:**
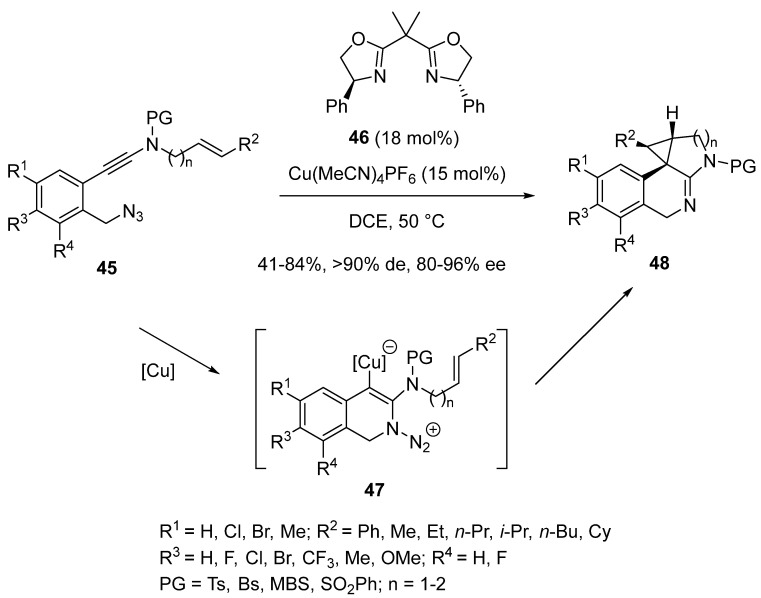
Domino cyclisation reaction of azide-ynamides [49].

Later in 2020, the same authors also described an enantioselective copper-catalysed domino cyclisation of azide-ynamides **45** (Scheme 19) [49]. The catalyst system arose from the reaction of 18 mol% of chiral bisoxazoline ligand **46** with 15 mol% of Cu(MeCN)_4_PF_6_. Carried out at 50 °C in DCE as the solvent, the process evolved through the generation of an imino copper carbene intermediate **47**. The latter subsequently underwent cyclopropanation to afford a series of chiral tetracyclic heterocycles **48** as almost single diastereomers (>90% de) in 41–84% yields and 80–96% ee. The catalyst tolerated different alkyl-substituted (azido)ynamides (R^2^ = alkyl) as well as phenyl-substituted substrates (R^2^ = Ph). Moreover, the phenyl ring of the ynamides could exhibit different types of substituents (R^1^,R^3^,R^4^). Along with Ts-protected ynamides (PG = Ts), various other *N*-protected groups, including Bs, MBS, and SO_2_Ph, were also tolerated.

**Scheme 20:**
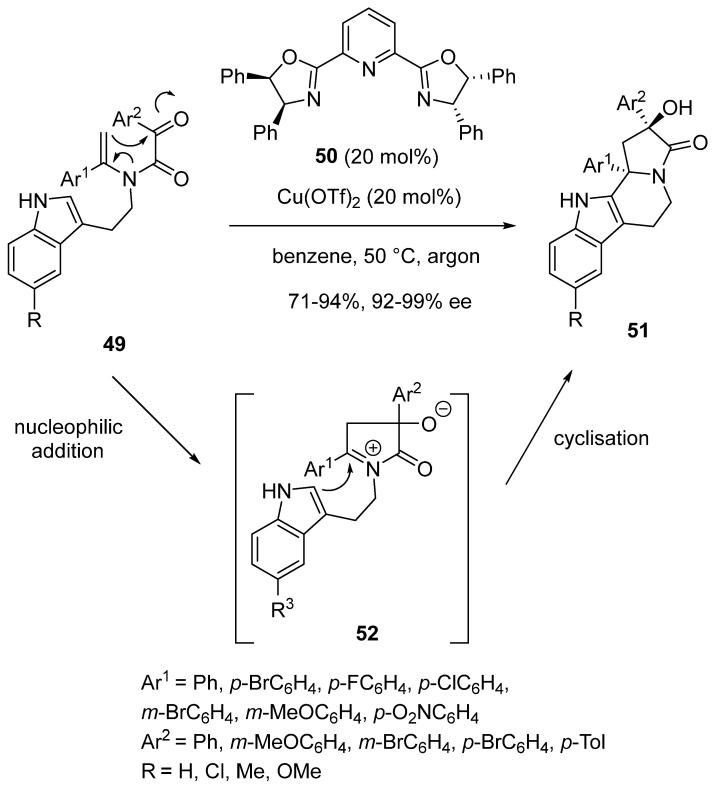
Domino cyclisation reaction of tertiary enamides [50].

Indolizino [8,7-b]indole structures are present in many bioactive products, which has made their synthesis challenging. In this context, Wang and Xu reported in 2021 an enantioselective domino cyclisation reaction involving tertiary enamides **49** as the substrates (Scheme 20) [50]. Promoted by 20 mol% of a copper catalyst in situ generated from Cu(OTf)_2_ and chiral Pybox ligand **50** in benzene at 50 °C under argon atmosphere, the cascade process delivered a diversity of chiral indolizino [8,7-b]indole derivatives **51** as single diastereomers with uniformly high yields (71–94%) and enantioselectivities (92–99% ee) whatever the electronic nature of substituents exhibited on the different phenyl rings. It evolved through the enantioselective intramolecular addition of the tertiary enamide to the ketonic carbonyl to give intermediate **52** followed by the diastereoselective trapping of acyliminium **52** by the tethered indole moiety, thus forming the desired indolizino [8,7-b]indole. The utility of this novel methodology was shown by converting products into bioactive harmicine analogues.

**Scheme 21:**
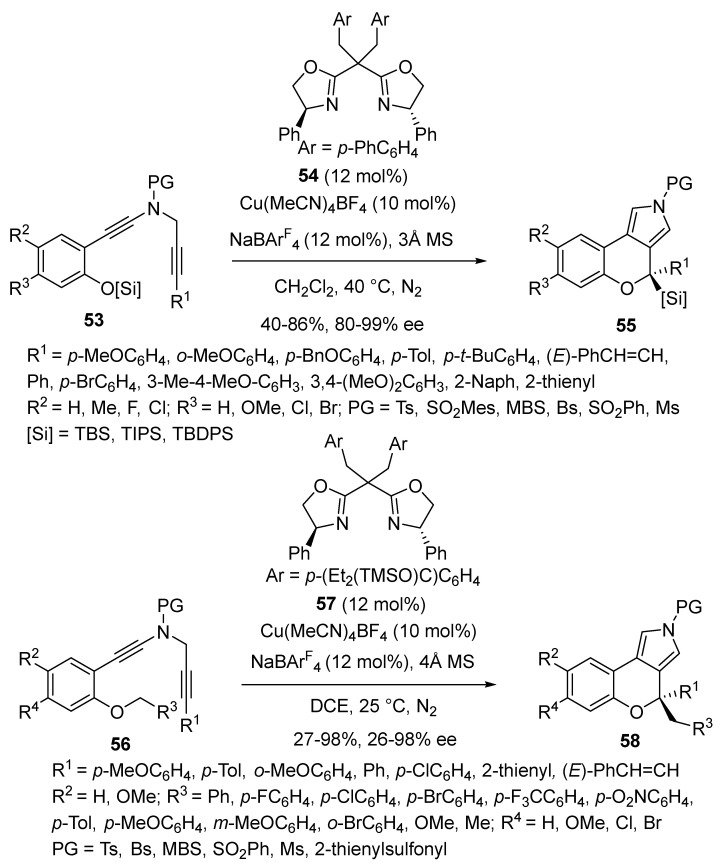
Domino cyclisation/[1,2]-Stevens-type rearrangement reactions of *N*-propargyl ynamides [51].

In 2022, Ye and Lu described a novel synthesis of chiral chromeno [3,4-c]pyrroles based on an enantioselective domino cyclisation/[1,2]-Stevens-type rearrangement reaction of *N*-propargyl ynamides (Scheme 21) [51]. For example, silylated *N*-propargyl ynamides **53** reacted at 40 °C in the presence of 10 mol% of Cu(MeCN)_4_BF_4_ and 12 mol% of chiral bisoxazoline ligand **54** in dichloromethane under N_2_ atmosphere in Schlenk tubes to give the corresponding chiral tricyclic pyrroles **55** bearing a quaternary stereocentre with moderate to high yields (40–86%) and uniformly high ee values (80–99% ee). Moreover, the domino reaction of related *N*-propargyl ynamides **56** performed at 25 °C in DCE as solvent in the presence of chiral bisoxazoline ligand **57** (12 mol%) combined with 10 mol% of Cu(MeCN)_4_BF_4_ led to desired chiral chromeno [3,4-c]pyrroles **58** with both moderate to excellent yields (27–98%) and enantioselectivities (26–98% ee), as presented in Scheme 21.

**Scheme 22:**
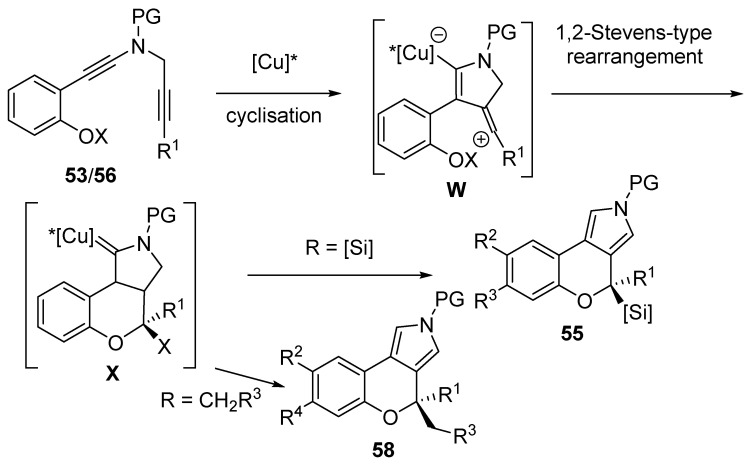
Mechanism for domino cyclisation/[1,2]-Stevens-type rearrangement reactions of *N*-propargyl ynamides [51].

A mechanism depicted in Scheme 22 proposes that the OX-substituted (X= [Si] or CH_2_R) vinyl cation **W** was generated through copper-catalysed cyclisation of the OX-substituted *N*-propargyl ynamide. Then, this carbene equivalent underwent cyclisation through asymmetric [1,2]-Stevens-type rearrangement, which provided donor/donor copper carbene **X**. Finally, a further [1,4]-H shift of the latter furnished the final chiral chromeno [3,4-c]pyrrole **55** or **58**. It must be noted that this work not only represented the first enantioselective [1,2]-Stevens-type rearrangement based on alkynes, but also constituted the first asymmetric formal carbene insertion into a Si—O bond.

### 2.5. Domino Reactions Initiated by Decarboxylative Propargylation

**Scheme 23:**
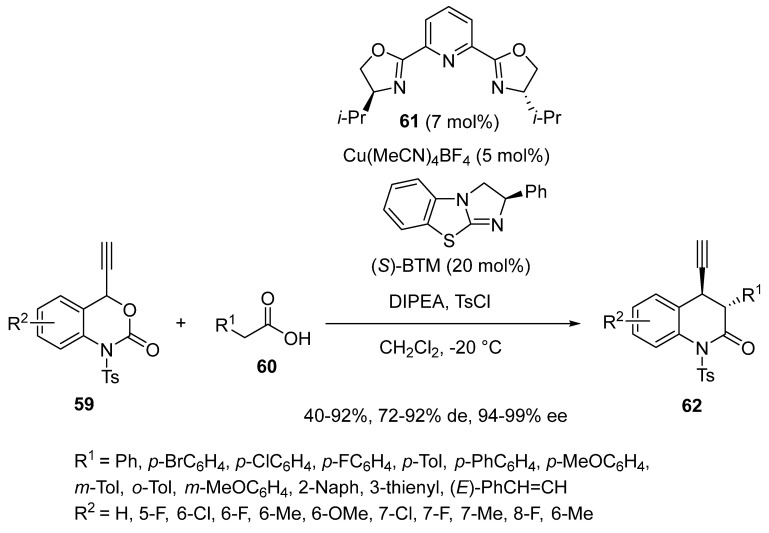
Domino decarboxylative propargylation/lactamisation reaction of 4-ethynyl benzooxazinones with carboxylic acids [52].

In 2017, a cooperative copper/Lewis base catalysis was applied by Gong et al. to promote an enantioselective [4 + 2] annulation of 4-ethynyl benzooxazinones **59** with carboxylic acids **60** (Scheme 23) [52]. This dealt with a domino decarboxylative propargylation/lactamisation reaction performed at −20 °C in dichloromethane in the presence of a chiral copper catalyst in situ generated from 5 mol% of Cu(MeCN)_4_PF_6_ and 7 mol% of chiral Pybox ligand **61**, associated to 20 mol% of chiral organocatalyst (*S*)-BTM. The process also required the use of DIPEA as a base, thus affording chiral 3,4-dihydroquinolin-2-ones **62** exhibiting two contiguous stereocentres with 40–92% yields and 72–92% de, combined with remarkable enantioselectivities (94–99% ee).

**Scheme 24:**
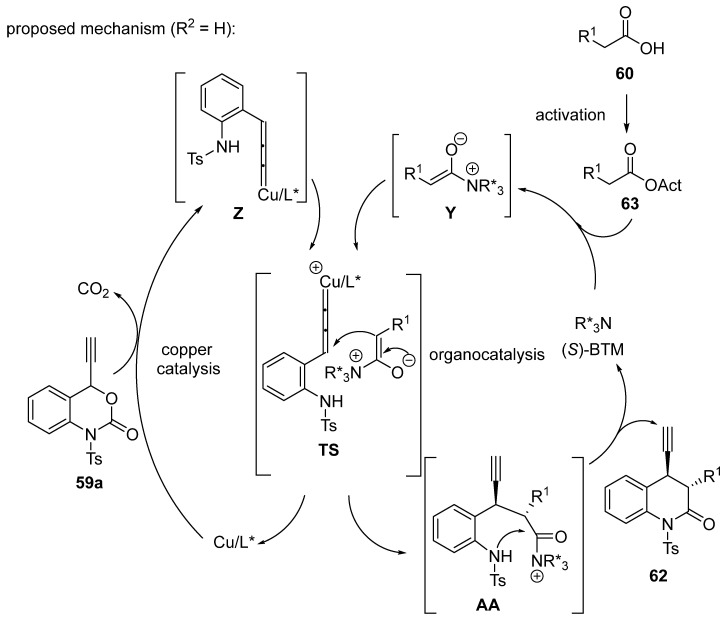
Mechanism for domino decarboxylative propargylation/lactamisation reaction of 4-ethynyl benzooxazinones with carboxylic acids [52].

As illustrated in Scheme 24, the mechanism began with the decarboxylation of the ethynyl benzoxazinanone in the presence of the copper catalyst, generating the copper allenylidene intermediate **Z**. Simultaneously, the carboxylic acid was converted into active electrophilic species **63**, which reacted with chiral tertiary amine catalyst (*S*)-BTM to give transient C1 ammonium enolate intermediate **Y**. Subsequently, the enantioselective addition of intermediate **Y** to the γ-carbon atom of intermediate **Z** generated chiral intermediate **AA** along with regenerated copper catalyst. Finally, intermediate **AA** underwent lactamisation to deliver the product and release the chiral organocatalyst.

**Scheme 25:**
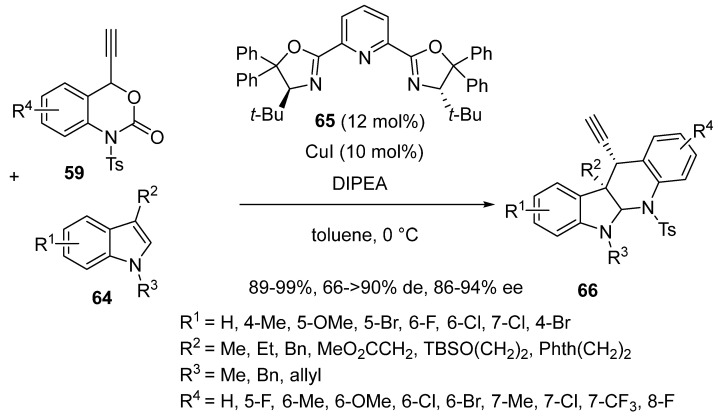
Domino decarboxylative propargylation/cyclisation reaction catalysed of 4-ethynyl benzooxazinones with indoles [53].

With the aim of developing a direct route to chiral tetrahydro-5H-indolo [2,3-b]quinolines, constituting the core structure of many indole alkaloids, You and Shao investigated in 2017 related cascade reactions between of 4-ethynyl benzooxazinones **59** and indoles **64** (Scheme 25) [53]. The synthesis evolved through an enantioselective decarboxylative propargylation/cyclisation reaction catalysed by 10 mol% of CuI and 12 mol% of chiral Pybox ligand **65** in toluene at 0 °C. In the presence of DIPEA as a base, a range of chiral tetrahydro-5*H*-indolo [2,3-b]quinolines **66** was produced in both high yields (89–99%) and enantioselectivities (86–94% ee) combined with moderate to excellent diastereoselectivities (66- > 90% de).

**Scheme 26:**
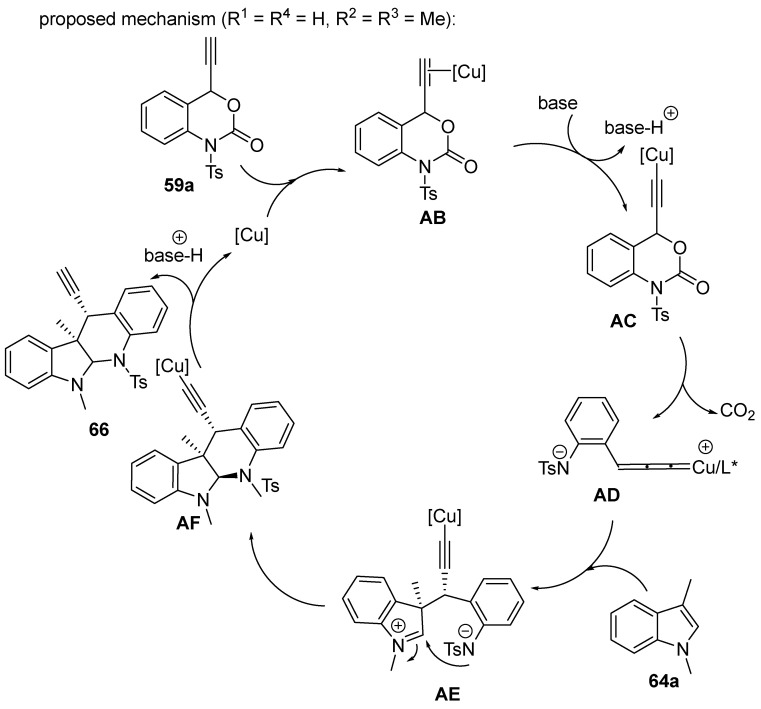
Mechanism for domino decarboxylative propargylation/cyclisation reaction catalysed of 4-ethynyl benzooxazinones with indoles [53].

A closely related mechanism as above (Scheme 24) is proposed in Scheme 26. In a first time, the benzooxazinone was activated by the copper catalyst to give complex **AB** which, upon deprotonation in the presence of DIPEA, afforded species **AC**. A subsequent decarboxylation of **AC** provided copper-allenylidene intermediate **AD**, which then underwent propargylic dearomatisation on the C3 position of the indole nucleus to give intermediate **AE**. Then, cyclisation of the latter afforded tetracyclic intermediate **AF,** which led to the final product after protonation, thus regenerating the catalyst.

**Scheme 27:**
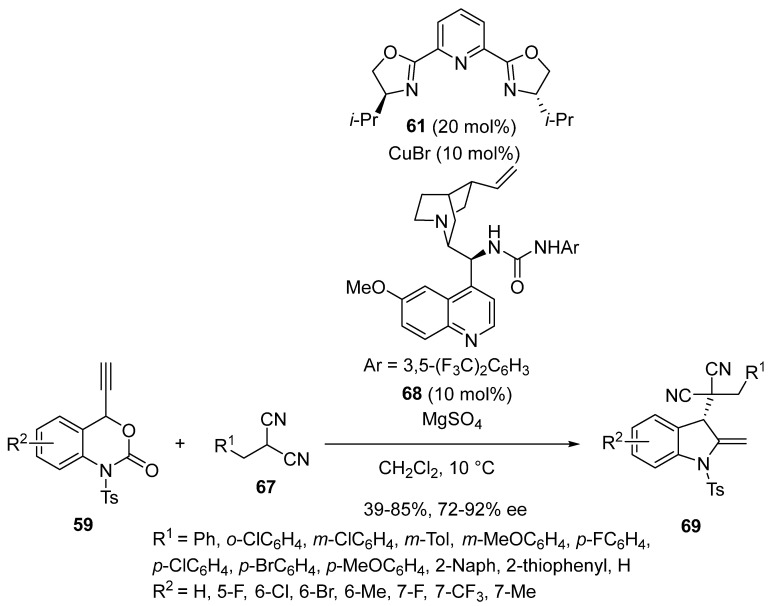
Domino decarboxylative propargylation/hydroamination reaction of ethynyl benzoxazinanones with malononitriles [54].

Later in 2018, the first enantioselective domino decarboxylative propargylation/hydroamination reaction between ethynyl benzoxazinanones **59** and malononitriles **67** was developed by Song et al. (Scheme 27) [54]. It was based on another organo/copper cooperative catalyst system constituting by the combination of 10 mol% of chiral cinchona alkaloid-derived urea **68** with a chiral copper catalyst derived from 10 mol% of CuBr and 20 mol% of chiral Pybox ligand **61**. The process enabled the formation at 10 °C in dichloromethane as the solvent the formation of chiral 3-indolin-malononitrile derivatives **66** displaying a high tolerance for functional groups. These products of high importance for medicinal chemistry were synthesised with 39–85% yields and 72–92% ee.

**Scheme 28:**
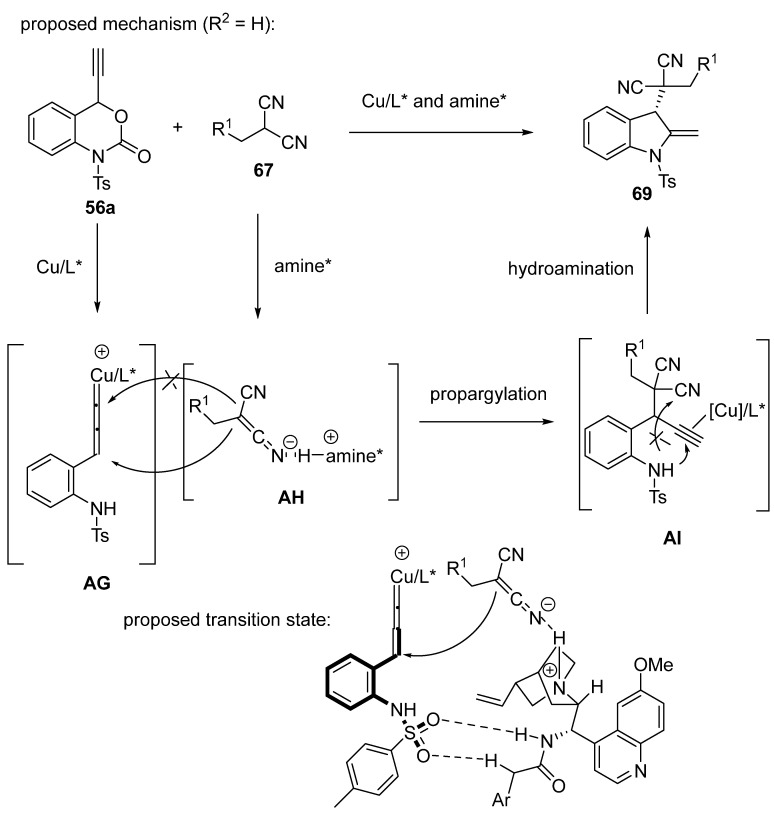
Mechanism for domino decarboxylative propargylation/hydroamination reaction of ethynyl benzoxazinanones with malononitriles [54].

The authors proposed the mechanism detailed in Scheme 28 in which electrophilic chiral copper-allenylidene intermediate **AG** was generated through copper-mediated decarboxylation of the ethynyl benzoxazinanone. Meanwhile, the combination of the malononitrile with the chiral organocatalyst provided nucleophilic chiral intermediate **AH**, which added to intermediate **AG** to give intermediate **AI**. Finally, the latter cyclised through hydroamination to provide the indoline malononitrile product. To explain the stereoselectivity of the reaction, the authors proposed the transition state depicted in Scheme 19 for the reaction between chiral intermediates **AG** and **AH**. The urea moiety of the organocatalyst served as an activator for the tosyl group of the copper-allenylidene intermediate **AG** through a dual hydrogen-bond interaction; while the tertiary amine part of the quinine unit acted as a Brønsted base to deprotonate and activate the malononitrile via a strong hydrogen bond with a nitrogen atom of the malononitrile.

**Scheme 29:**
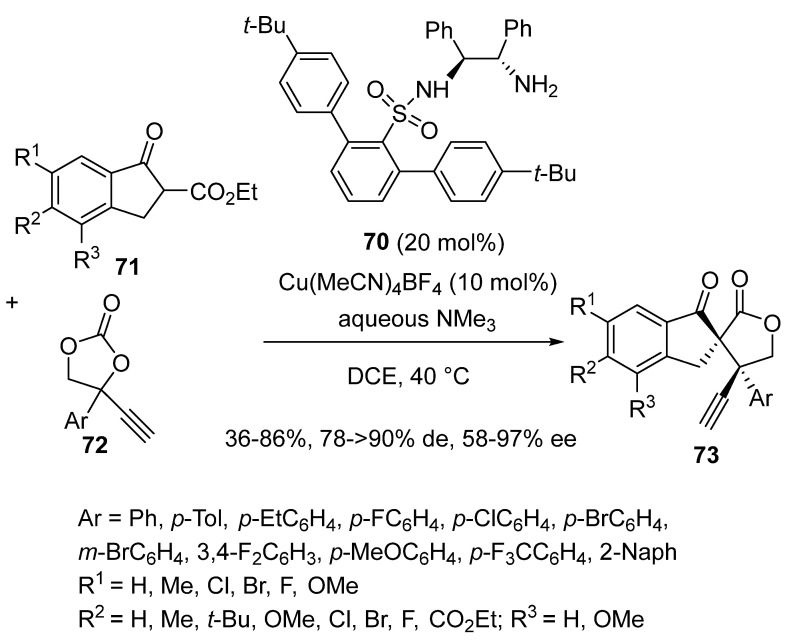
Domino decarboxylative propargylation/cyclisation reaction of 1-indanone-2-carboxylates with ethynylethylene carbonates [55].

In 2023, chiral diphenylethylenediamine-based sulfonamide ligand **70** (20 mol%) was combined by Chen and Ning with Cu(MeCN)_4_BF_4_ (10 mol%) in order to promote an asymmetric decarboxylative propargylation between 1-indanone-2-carboxylates **71** and ethynylethylene carbonates **72** (Scheme 29) [55]. Carried out at 40 °C in DCE as the solvent in the presence of aqueous NMe_3_ as a base, the domino decarboxylative propargylation/cyclisation reaction afforded chiral spirolactones **73** bearing vicinal quaternary stereocentres with moderate to high yields (36–86%), generally very high diastereoselectivities (78- > 90% de) and moderate to excellent ee values (58–97% ee).

**Scheme 30:**
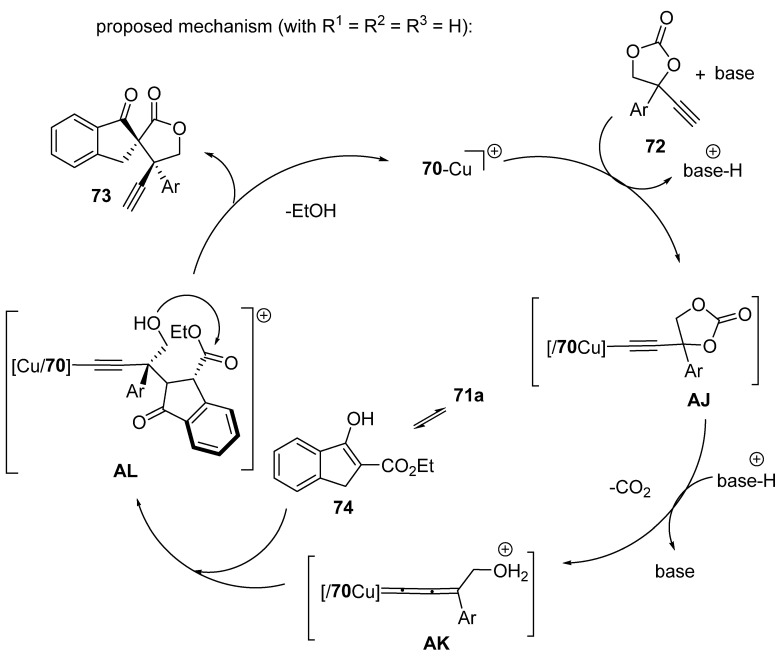
Mechanism for domino decarboxylative propargylation/cyclisation reaction of 1-indanone-2-carboxylates with ethynylethylene carbonates [55].

The mechanism detailed in Scheme 30 shows that the process began with the copper-catalysed decarboxylation of the ethynylethylene carbonate through intermediate **AK** to give the copper-allenylidene intermediate **AL**. Subsequently, the stereoselective trapping of the latter by compound **74** generated intermediate **AL,** which further underwent intramolecular cyclisation to afford the final spirolactone.

**Scheme 31:**
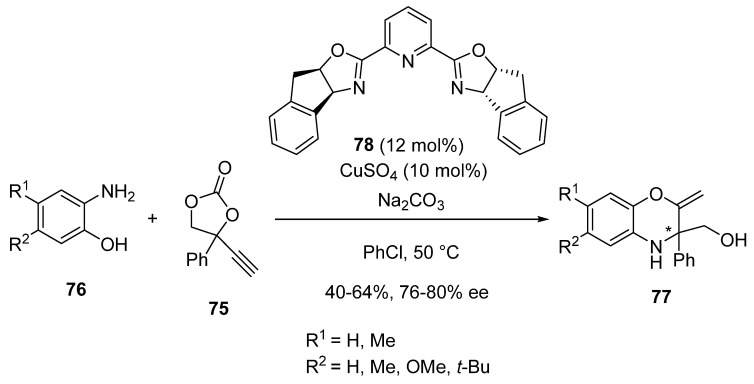
Domino decarboxylative propargylic substitution/cyclisation reaction of 2-aminophenols with an ethynylethylene carbonate [56].

Later in 2024, asymmetric domino decarboxylative propargylic substitution/cyclisation reactions of ethynylethylene carbonate **75** and 2-aminophenols **76** were reported by Huang et al. [56]. As shown in Scheme 31, these formal [4 + 2] cycloaddition reactions led to chiral 3,4-dihydro-2H-benzo[b][1,4]oxazines **77** with moderate yields (40–64%) and good enantioselectivities (76–80% ee) when catalysed at 50 °C in chlorobenzene as solvent by a chiral copper complex in situ generated from 10 mol% of CuSO_4_ and 12 mol% of chiral bisoxazoline ligand **78**.

**Scheme 32:**
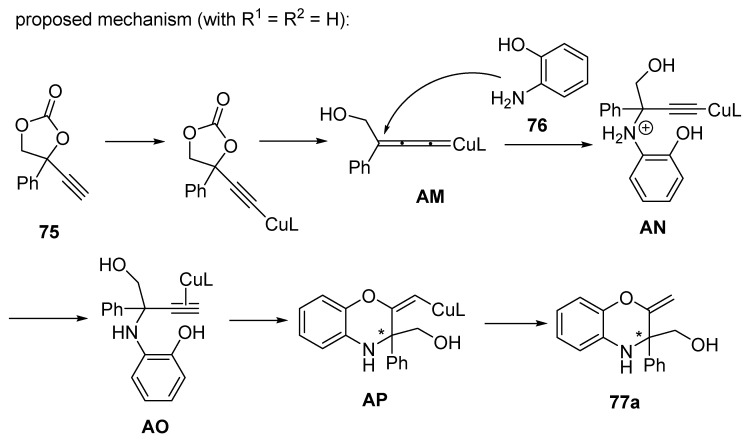
Mechanism for domino decarboxylative propargylic substitution/cyclisation reaction of 2-aminophenols with an ethynylethylene carbonate [56].

The domino process evolved through successive propargylic amination, intramolecular alkyne hydroxylation, and ring closure (Scheme 32). Indeed, the reaction started with the formation of copper allenylidene intermediate **AM** through deprotonation and CO_2_ elimination of the ethynylethylene carbonate under basic conditions. Subsequently, the amino group of the 2-aminophenol underwent a nucleophilic attack on copper allenyl intermediate **AM**, resulting in the formation of copper acetylide **AN**. Then, a proton transfer of **AN** provided γ-aminopropyne intermediate **AO**. Then, copper activated the alkyne group, and the hydroxyl group of the 2-aminophenol acted as second nucleophilic group to complete the cyclisation reaction, delivering the final product.

### 2.6. Mannich-Initiated Domino Reactions

**Scheme 33:**
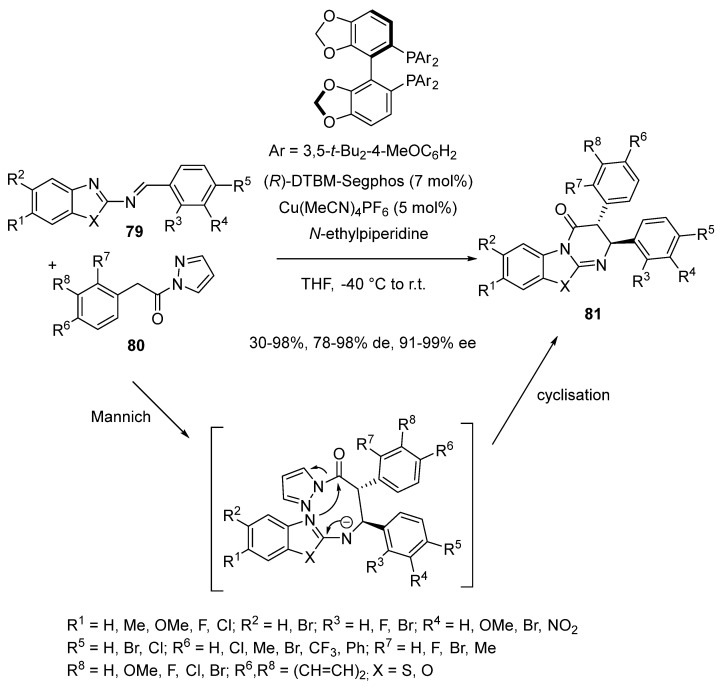
Domino Mannich/cyclisation reaction of 2-benzothiazolimines with *N*-acylpyrazoles [57].

In 2021, Chen and Dong described an enantioselective domino Mannich/cyclisation reaction of 2-benzothiazolimines (X = S) **79** with *N*-acylpyrazoles **80** (Scheme 33) [57]. It was promoted at −40 °C to room temperature by a chiral catalyst in situ generated from 5 mol% of Cu(MeCN)_4_PF_6_ and 7 mol% of (*R*)-DTBM-Segphos in THF in the presence of *N-*ethylpiperidine as a base, thus producing chiral benzothiazolopyrimidines **81** (X = S) with 30–98% yields, 78–98% de, and 91–99% ee. The catalyst system tolerated the presence of various substituents on the phenyl rings of both the two substrates. Furthermore, in addition to benzothiazole units (X = S), a benzoxazole substrate (X = O) afforded the corresponding product with 72% yield, 98% de, and 98% ee.

**Scheme 34:**
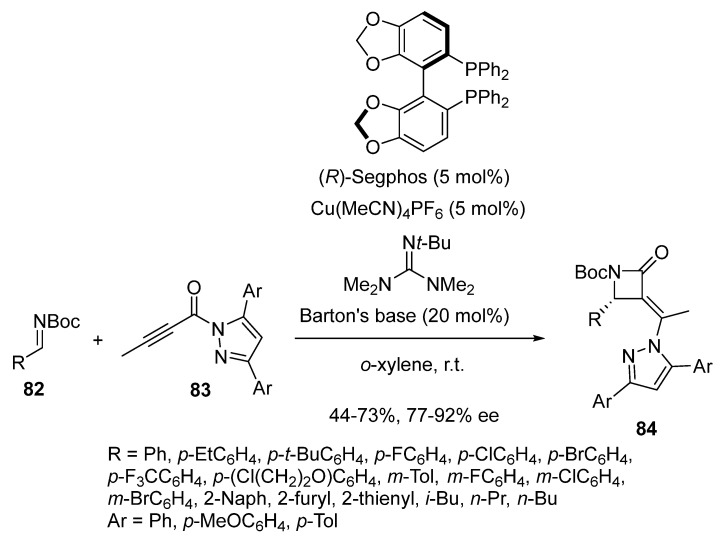
Domino Mannich-type/transamination/aza-Michael/protonation reaction of aldimines with pyrazoleamides [58].

Later in 2022, Yin et al. disclosed an enantioselective cascade cyclisation of aldimines **82** with alkyne-tethered pyrazoleamides **83**, allowing a novel entry to chiral *β*-lactams (Scheme 34) [58]. Performed at room temperature in *o*-xylene in the presence of 5 mol% of a chiral copper catalyst generated from Cu(MeCN)_4_PF_6_ and (*R*)-Segphos, the domino reaction resulted in the formation of chiral *β*-lactams **84** including a pyrazole moiety with 44–73% yields and 77–92% ee. Along with (hetero)aromatic aldimines, aliphatic aldimines were also compatible substrates, leading to the corresponding products with comparable yields (54–73%) albeit associated with lower enantioselectivities (73–82% ee).

**Scheme 35:**
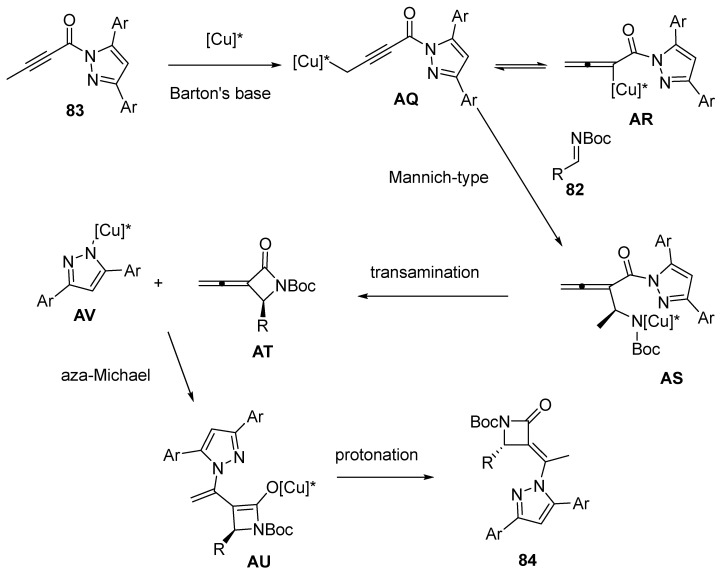
Mechanism for domino Mannich-type/transamination/aza-Michael/protonation reaction of aldimines with pyrazoleamides [58].

The authors proposed the mechanism depicted in Scheme 35, evolving through successive Mannich-type reaction, transamination, aza-Michael addition, and protonation, which began with the formation of intermediate **AQ** from the pyrazoleamide through *γ*-deprotonation in the presence of copper(I)-biphosphine complex and Barton′s base. Intermediate **AQ** could be in equilibrium with allenyl copper(I) species **AR**; however, the addition of **AQ** to the aldimine was favoured. Therefore, *γ*-addition of allenyl copper(I) species **AR** to the aldimine was significantly attenuated, which led to an excellent α-regioselectivity. Indeed, an *α*-selective Mannich-type reaction occurred predominantly between intermediate **AQ** and the aldimine to give intermediate **AS**. The latter was subsequently submitted to an intramolecular transamination to provide intermediate **AV** along with nucleophilic species **AT**. Then, an aza-Michael addition of **AT** to **AV** led to intermediate **AU**, which underwent protonation to deliver the final *β*-lactam.

### 2.7. Arylation-Initiated Domino Reactions

**Scheme 36:**
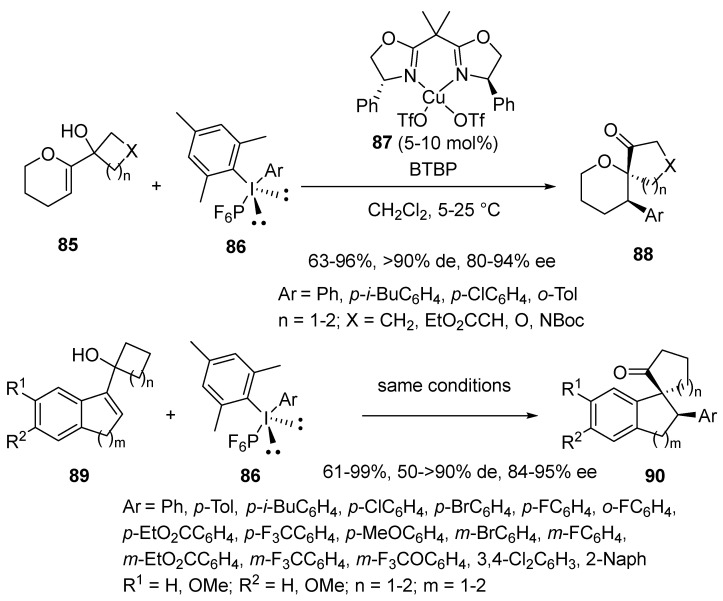
Domino arylation/rearrangement reactions of allylic alcohols with diaryliodonium salts [59].

In 2017, Gaunt and Lukamto disclosed a novel asymmetric synthesis of spirocyclic ketones based on an enantioselective copper-catalysed arylative semipinacol rearrangement of allylic alcohols [59]. The domino arylation/rearrangement reaction of allylic alcohols **85** with diaryliodonium salts **86** was promoted at 5–25 °C by 5–10 mol% of preformed catalyst **87** including a chiral bisoxazoline ligand in dichloromethane as the solvent in the presence of 2,6-di-*tert*-butylpyridine (DTBP) as a base, thus leading to the corresponding chiral spirocyclic ketones **88** as single diastereomers (>90% de) with 63–96% yields and 80–94% ee (Scheme 36). In addition to tetrahydropyran derivatives **85**, indene- and tetrahydronapthalene-based substrates **89**, exhibiting either cyclobutyl or cyclopentyl carbinol substituents, reacted smoothly to afford desired products **90** in 61–99% yields, 50- > 90% de, and 84–95% ee (Scheme 24). It must be noted that in almost all cases of these substrates, the spirolactones were obtained as single diastereomers.

**Scheme 37:**
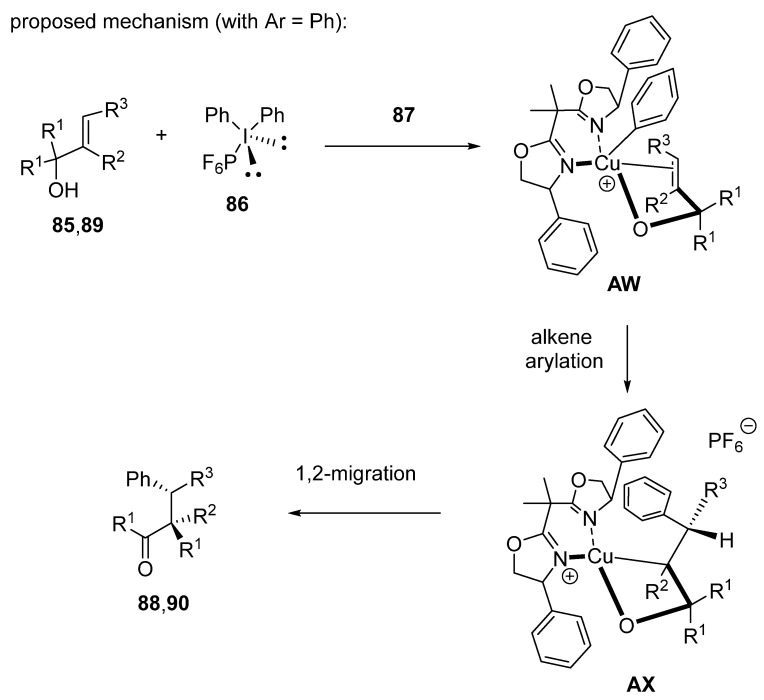
Mechanism for domino arylation/rearrangement reactions of allylic alcohols with diaryliodonium salts [59].

A possible mechanism is detailed in Scheme 37, beginning with the formation of intermediate **AW** from the interaction between the allylic alcohol, the diaryliodonium salt, and the chiral copper complex. Then, insertion of the Cu(III)−Ar bond to the alkene generated the Cu(III)−alkyl intermediate **AX**. The latter subsequently triggered the stereoselective 1,2-migration of one of the carbinol substituents, thus completing an arylation-driven semipinacol rearrangement to deliver the final α,α′-disubstituted-β-arylketone.

**Scheme 38:**
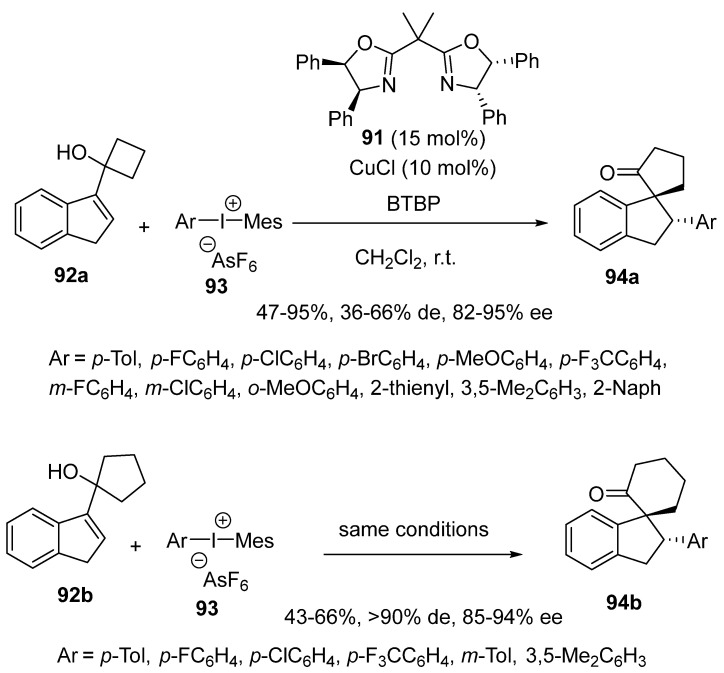
Domino arylation/rearrangement reactions of vinylcyclobutanol/vinylcyclopentanol with diaryliodonium salts [60].

Almost concomitantly, Zhu et al. also investigated these reactions in the presence of a chiral copper catalyst in situ formed from 10 mol% of CuCl and 15 mol% of chiral bisoxazoline ligand **91** (Scheme 38) [60]. At room temperature in dichloromethane as the solvent, the asymmetric domino arylation/rearrangement reaction of vinylcyclobutanol **92a** with mesityl(aryl)iodonium trifluoromethanesulfonate **93** afforded desired chiral spirocyclopentanones **94a** with 47–95% yields and 82–95% ee albeit combined with moderate diastereoselectivities (36–66% de). On the other hand, the reaction of vinylcyclopentanol **92b** with diaryliodonium salts **93** with different electronic properties led to the corresponding chiral spirocyclohexanones **94b** as almost single diastereomers (>90% de) in 43–66% yields and uniformly high ee values (85–94% ee). The high diastereoselectivity observed in the reaction of vinylcyclopentanol in comparison with that of vinylcyclobutanol was noteworthy and could be explained by the fact that the diminished ring strain release going from a five- to a six-membered ring rendered the 1,2-migration process more selective.

### 2.8. Domino Yne-Allylic Substitution/Cyclisation Reactions

**Scheme 39:**
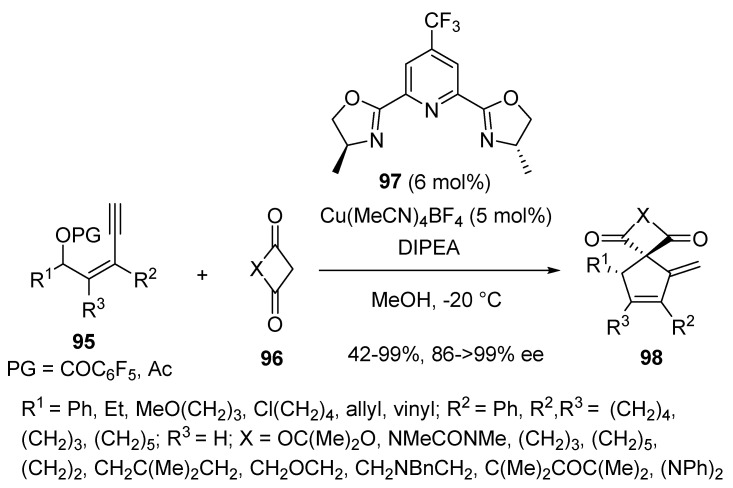
Domino yne-allylic substitution/Conia-ene cyclisation reaction of enyne esters with cyclic 1,3-dicarbonyl compounds [62].

A diversity of asymmetric copper-catalysed domino yne-allylic substitution/cyclisation reactions have been described in the last few years [61]. For example in 2022, Qi and Xu developed the first copper-catalysed enantioselective [4 + 1] annulation of enyne esters **95** with cyclic 1,3-dicarbonyl compounds **96**, evolving through a domino yne-allylic substitution/Conia-ene cyclisation reaction (Scheme 39) [62]. It involved 5 mol% of Cu(MeCN)_4_BF_4_ associated to 6 mol% of chiral Pybox ligand **97** as catalyst system in methanol at -20 °C in the presence of DIPEA as base. The first formed yne-allylic substitution products further underwent an intramolecular cyclisation to regioselectively afford chiral spirocyclic products **98** in moderate to quantitative yields (42–99%) and homogeneously high enantioselectivities (86- > 99% ee). A wide variety of 1,3-dicarbonyl compounds were compatible, including four-, five-, and six-membered 1,3-diketones, which could include heteroatoms. Moreover, variously substituted yne-allylic esters were tolerated.

**Scheme 40:**
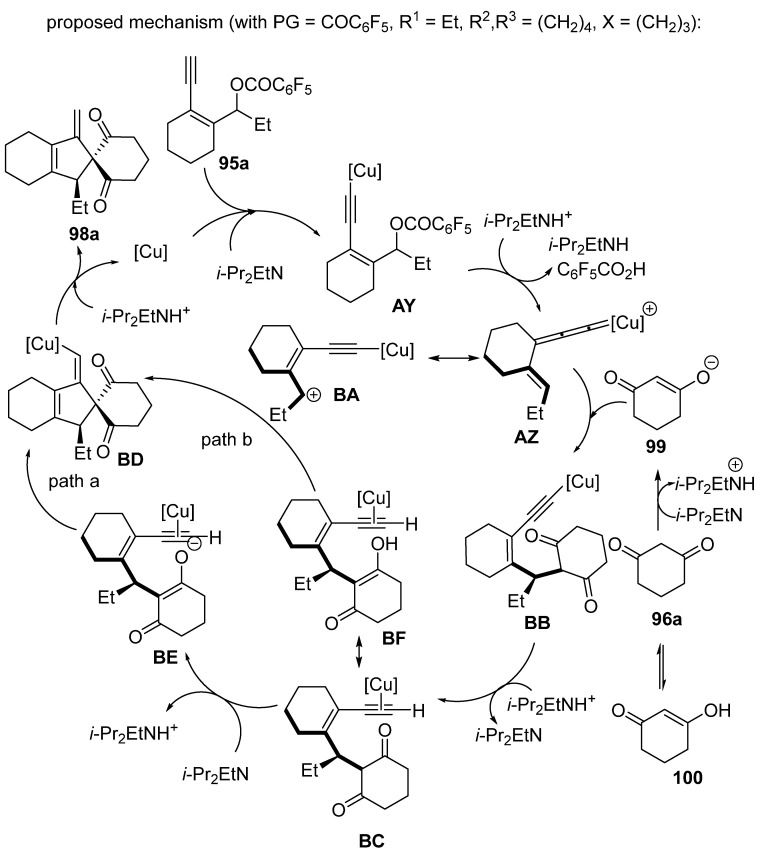
Mechanism for domino yne-allylic substitution/Conia-ene cyclisation reaction of enyne esters with cyclic 1,3-dicarbonyl compounds [62].

The authors proposed the mechanism detailed in Scheme 40, which began with the activation of the terminal alkyne of substrate **95a** by the chiral copper catalyst, thus generating copper acetylide species **AY**. Then, the latter eliminated a pentafluorobenzoate group to give copper vinylvinylidene intermediate **AZ** along with its copper acetylide resonance species **BA**. Subsequently, a nucleophilic addition of in situ formed enolate anion **99** preferentially occurred at the ε site of the copper vinylvinylidene species **AZ**, thus producing intermediate **BB**. Alternatively, enol form **100** could also undergo the nucleophilic attack. Then, protodemetalation of intermediate **BB** generated species **BC**. The latter was further submitted to a Conia-ene cyclisation, which afforded intermediate **BD** through intermediate **BE** (path a) or **BF** (path b). Finally, the protodemetalation of intermediate **BD** furnished the spirocyclic product along with regenerated catalyst.

**Scheme 41:**
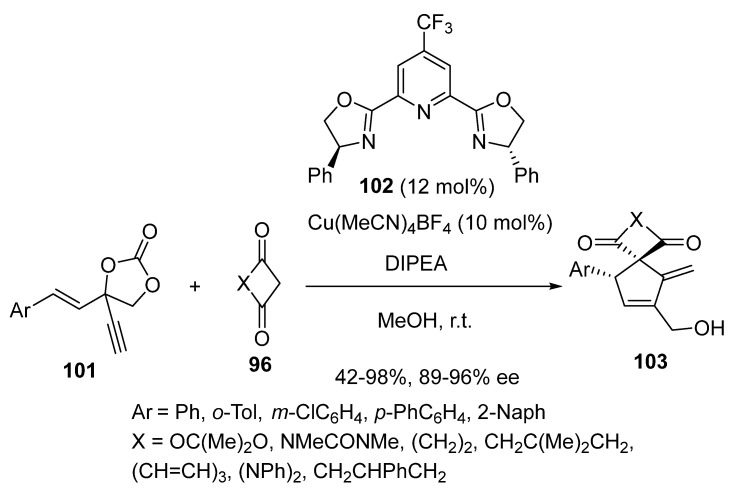
Domino yne-allylic substitution/Conia-ene cyclisation reaction of vinyl ethynylethylene carbonates with cyclic 1,3-dicarbonyl compounds [63].

Later in 2023, Han and Huang reported related asymmetric domino substitution/Conia-ene cyclisation reactions between vinyl ethynylethylene carbonates **101** and cyclic 1,3-dicarbonyl compounds **96** (Scheme 41) [63]. These formal [4 + 1] cycloadditions performed at room temperature in methanol in the presence of DIPEA as base and a chiral copper catalyst in situ generated from 10 mol% of Cu(MeCN)_4_BF_4_ and 12 mol% of chiral Pybox ligand **102** allowed the regioselective synthesis of chiral spirocyclic products **103** with 42–98% yields and 89–96% ee.

**Scheme 42:**
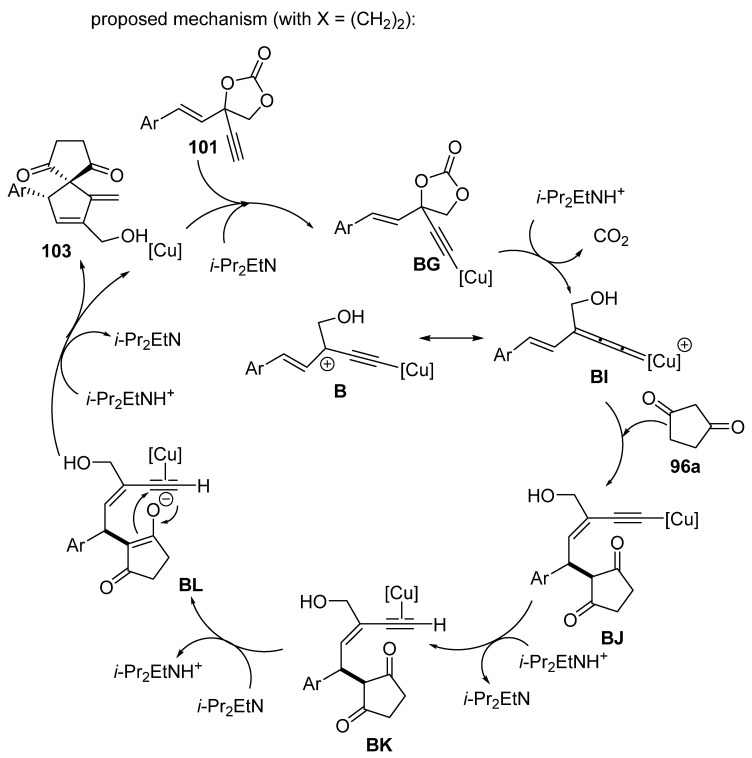
Mechanism for domino yne-allylic substitution/Conia-ene cyclisation reaction of vinyl ethynylethylene carbonates with cyclic 1,3-dicarbonyl compounds [63].

A catalytic cycle is proposed in Scheme 42, which started with the formation of copper acetylide **I** in the presence of the catalyst and DIPEA. Then, intermediate **BG** underwent elimination of CO_2_ and protonation to provide propargylic cation **BH**, which is a resonance form of copper allenylidene intermediate **BI**. Subsequently, the nucleophilic attack of **101a** at the ε position of intermediate **BI** afforded intermediate **BJ**, due to the steric hindrance of the bulky ligand blocking the γ position. Subsequently, a protonation and a Conia-ene reaction delivered the spirocyclic product.

**Scheme 43:**
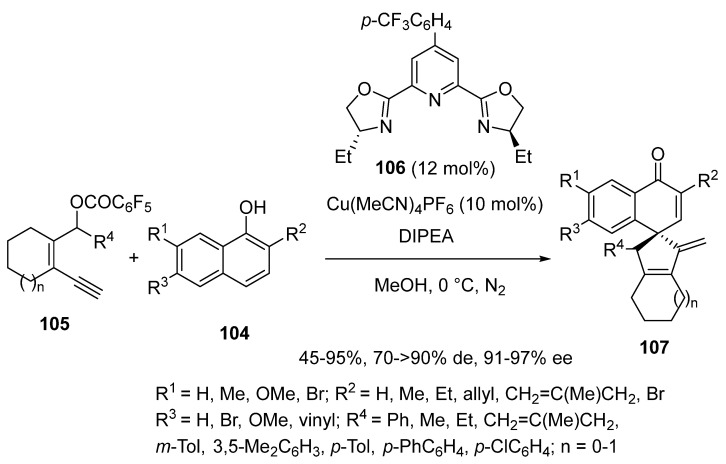
Domino yne-allylic substitution/cyclisation reaction of enyne esters with 1-naphthols [64].

An unprecedented dearomatisation of nonfunctionalised 1-naphthols **104** was reported in 2024 by Li, Yu and Liu via copper-catalysed remote enantioselective [4 + 1] spiroannulation with enyne esters **105** [64]. This chemo-, regio-, and stereoselective domino reaction evolved at 0 °C in methanol through nucleophilic substitution followed by cyclisation under catalysis with a combination of 10 mol% of Cu(MeCN)_4_PF_6_ and 12 mol% of chiral Pybox ligand **106** (Scheme 43). Performed in the presence of DIPEA as base under N_2_ atmosphere, the protocol allowed the formation of chiral spirocyclic enones **107** bearing a chiral quaternary stereocentre with 45–95% yields, 70- > 90% de, and 91–97% ee.

**Scheme 44:**
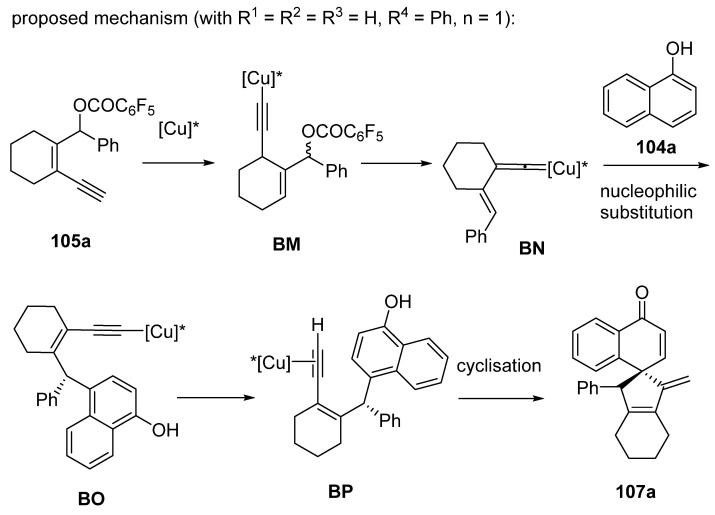
Mechanism for domino yne-allylic substitution/cyclisation reaction of enyne esters with 1-naphthols [64].

A possible mechanism is depicted in Scheme 44, beginning with the formation of copper alkyne species **BM** from the reaction between the alkyne unit of the enyne ester substrate and the chiral catalyst. Then, the latter underwent elimination of the ester group to give copper allenylidene intermediate **BN**. Subsequent nucleophilic addition of the 1-naphthol in the presence of DIPEA generated intermediate **BO**. Protodemetalation of **BO** provided intermediate **BP**, which finally cyclised into the spirocyclic product.

**Scheme 45:**
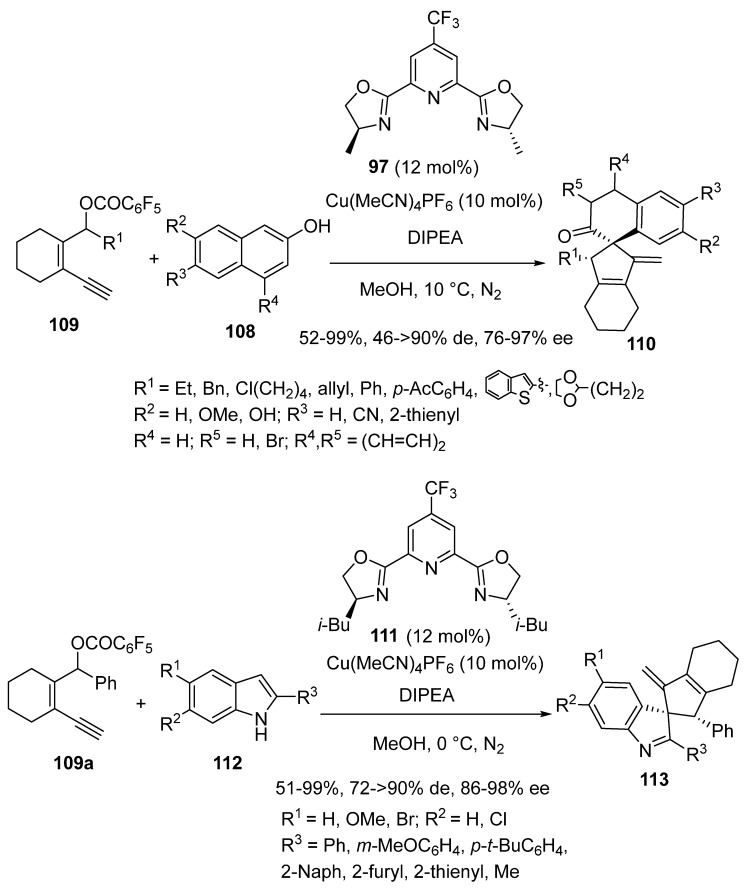
Domino yne-allylic substitution/cyclisation reactions of enyne esters with 2-naphthols/indoles [65].

In the same year, Qi and Xu investigated related dearomative spiroannulation of 2-naphthols **108** with enyne esters **109** [65]. In this case, Pybox ligand **97** was found optimal when employed at 12 mol% of catalyst loading with 10 mol% of Cu(MeCN)_4_PF_6_ in methanol at 10 °C under N_2_ atmosphere (Scheme 45). The desired chiral naphthalene-2-ones **110** were regioselectively produced with moderate to quantitative yields (52–99%), diastereo- (46- > 90% de) and enantioselectivities (76–97% ee). Using related chiral ligand **111** at 0 °C, the domino nucleophilic substitution/cyclisation reaction between indoles **112** and enyne esters **109** also proceeded smoothly, resulting in the formation of the corresponding chiral spiroindolenines **113** with 51–99% yields, 72- > 90% de, and 86–98% ee (Scheme 45).

**Scheme 46:**
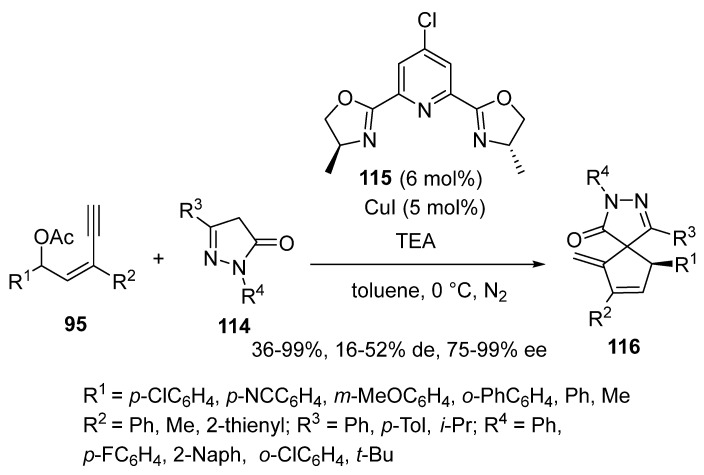
Domino yne-allylic substitution/cyclisation reaction of enyne esters with pyrazolones [66].

Spiropyrazolones constitute the scaffolds of many bioactive and natural products. Consequently, considerable efforts have been devoted to the asymmetric synthesis of these molecules. For example, Xu et al. reported in 2024 the asymmetric [4 + 1] annulation of enyne esters **95** with pyrazolones **114** promoted by a chiral copper catalyst in situ generated from 6 mol% of chiral Pybox ligand **115** and 5 mol% of CuI in toluene at 0 °C under N_2_ atmosphere (Scheme 46) [66]. A range of complex chiral spiropyrazolones **116** were produced in moderate to high yields (36–99%), moderate diastereoselectivities (16–52% de), and moderate to excellent enantioselectivities (75–99% ee). The catalyst system tolerated a variety of aryl yne-allylic acetates exhibiting either electron-donating or electron-withdrawing substituents, which afforded the corresponding spiropyrazolones in excellent yields with good enantioselectivities. Furthermore, a series of pyrazolones bearing different aryl-substituents at the 3-position of the pyrazolones reacted smoothly. *N*-aryl- as well as *N*-alkyl-substituted pyrazolones were compatible.

**Scheme 47:**
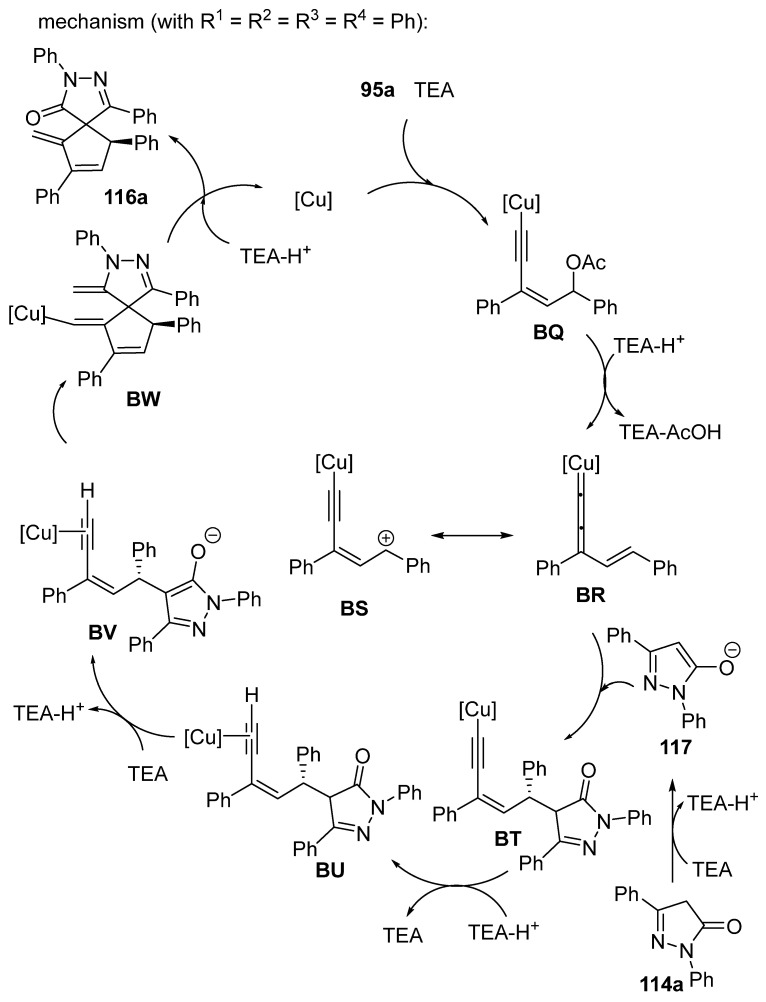
Mechanism for domino yne-allylic substitution/cyclisation reaction of enyne esters with pyrazolones [66].

The authors proposed the mechanism detailed in Scheme 47 beginning with the formation of copper acetylide intermediate **BQ** from the reaction between the chiral copper catalyst and the terminal alkyne. Subsequently, elimination of an acetyl group from **BQ** generated copper allenylidene intermediate **BR** along with its resonance form acetylide intermediate **BS**. Then, an in situ formed enolate anion **117** underwent nucleophilic addition at the ε site of intermediate **BR** to give intermediate **BT**. The further protodemetalation of **BT** produced intermediate **BU** and then **BV** through protonation. The Conia-ene cyclisation of intermediate **BV** occurred to deliver intermediate **BW,** which furnished through final protodemetalation the desired product **116a** along with regenerated copper catalyst.

**Scheme 48:**
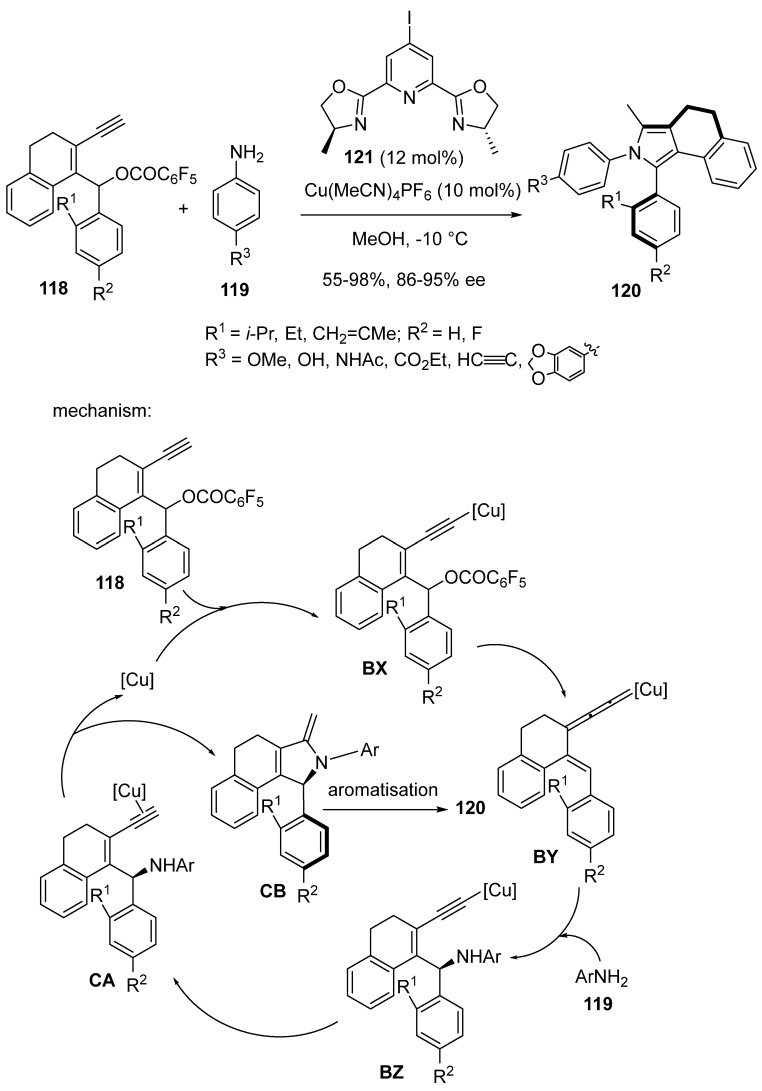
Domino yne-allylic substitution/cyclisation/aromatisation reaction of enyne esters with anilines [67].

In the same year, these authors also reported an enantioselective [4 + 1] annulation between enyne esters **118** and anilines **119**, evolving through successive nucleophilic substitution, cyclisation and aromatisation to synthesise axially chiral arylpyrroles **120** with 55–98% yields and uniformly high ee values (86–95% ee), as illustrated in Scheme 48 [67]. The reaction was performed in methanol at −10 °C in the presence of 10 mol% of Cu(MeCN)_4_PF_6_ and 12 mol% of chiral Pybox ligand **121**. A mechanism is proposed in Scheme 48, starting with the coordination and deprotonation of the terminal alkyne with the copper catalyst to provide copper acetylide intermediate **BX**. Then, the latter eliminated the leaving group to generate copper vinylallenylidene species **BY**. Subsequently, **BY** underwent remote amination to form intermediate **BZ** bearing a stereocentre, which further cyclised into species **CB**. Finally, aromatisation of **CB** delivered the product.

**Scheme 49:**
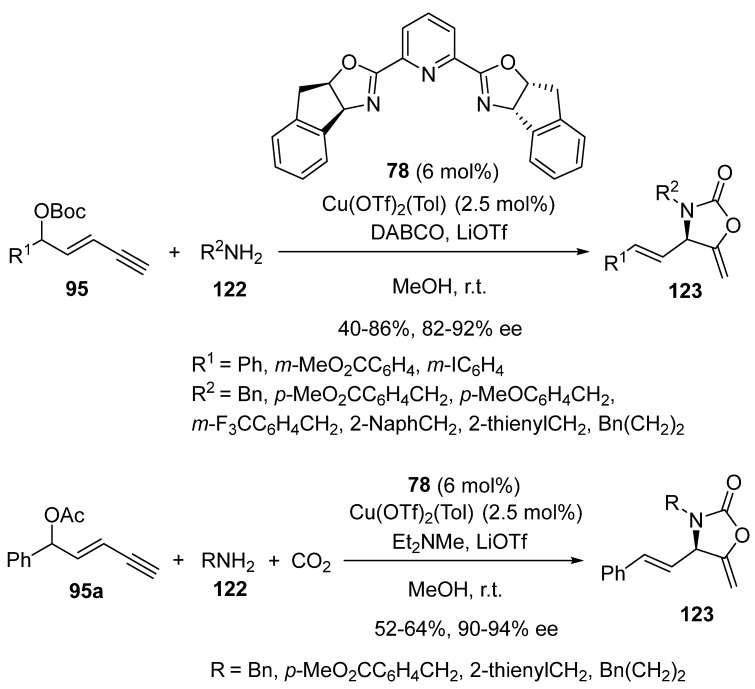
Domino decarboxylation/yne-allylic substitution/CO_2_ capture/cyclisation reaction of enyne esters with amines and three-component version under CO_2_ atmosphere [68].

In 2024, He et al. investigated the combination of asymmetric copper-catalysed domino yne-allylic substitution/cyclisation reactions with CO_2_ shuttling (Scheme 49) [68]. The process performed at room temperature in methanol with DABCO as base involved enyne esters **95** and alkyl amines **122** as the substrates and a chiral copper catalyst derived from 2.5 mol% of Cu(OTf)_2_(Tol) and 6 mol% of Pybox ligand **78**. The domino reaction evolved through successive decarboxylation, alkynylallylic substitution, capture the CO_2_, and cyclisation to furnish a range of chiral oxazolidinones **123** in 40–86% yields and 82–92% ee. In a second time, the authors showed that the protocol could also be performed under CO_2_ atmosphere (Scheme 49). Indeed, the strategy was also suitable for a three-component reaction between enyne esters **95**, alkyl amines **122** and CO_2_ performed under the same conditions to produce chiral oxazolidinone scaffolds **123** in comparable yields (52–64%) and enantioselectivities (90–94% ee) to those obtained through the aforementioned CO_2_ shuttling route. It must be noted that this work should be situated in Section 3 focusing on multicomponent reactions but for commodity it was decided to locate it in this place. The utility of these novel methodologies was demonstrated in a concise total synthesis of natural product (−)-cytoxazone.

**Scheme 50:**
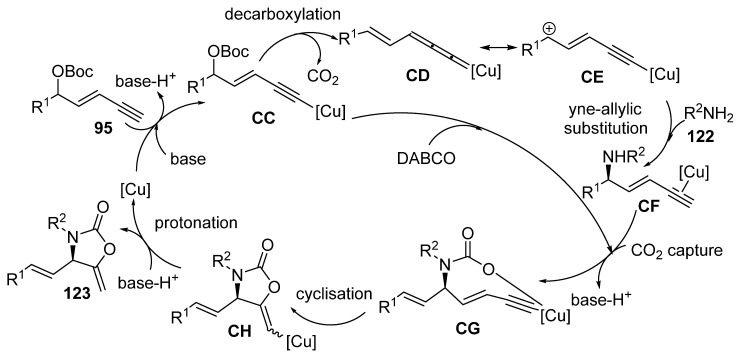
Mechanism for domino decarboxylation/yne-allylic substitution/CO_2_ capture/cyclisation reaction of enyne esters with amines and three-component version under CO_2_ atmosphere [68].

Mechanistically, in a first time the copper catalyst reacted with terminal alkyne **95a** in the presence of DABCO to generate intermediate **CC** (Scheme 50). Then, the copper allenylidene species **CD** was formed from the cleavage of the carbonate leaving group along with the release of CO_2_. The following asymmetric propargylic substitution with the primary amine **122** yielded **CF**, which further captured the released CO_2_ that might be activated by DABCO to form species **CG**. Subsequently, the intramolecular cyclisation of **CG** yielded intermediate **CH** which was further protonated to deliver the final product along with regenerated catalyst.

### 2.9. Miscellaneous Domino Reactions

#### 2.9.1. Single Copper Catalysis

**Scheme 51:**
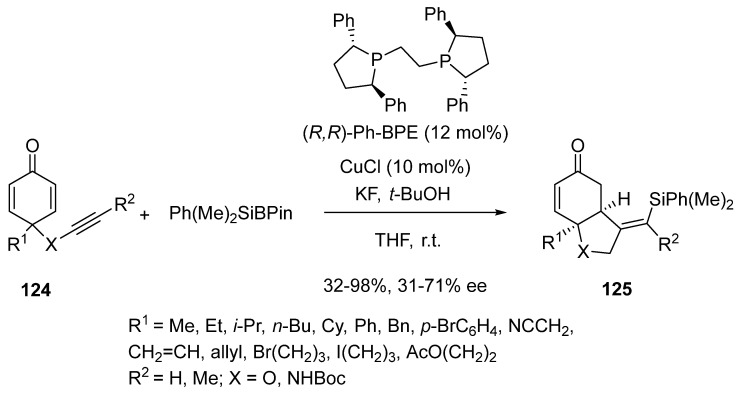
Domino silylation/cyclisation of alkyne-tethered cyclohexadienones with PhMe_2_SiBPin [69].

In 2019, Lin and Tian described the first enantioselective copper-catalysed domino silylation/cyclisation reaction of cyclohexadienone-containing 1,6-enynes **124** with PhMe_2_SiBPin (Scheme 51) [69]. The reaction was carried out at room temperature in THF under argon atmosphere in the presence of a combination of 10 mol% of CuCl and 12 mol% of chiral biphosphine ligand (*R*,*R*)-Ph-BPE, resulting in the regioselective formation of chiral *cis*-hydrobenzofuran (X = O) and *cis*-hydroindole (X = NHBoc) derivatives **125** bearing two contiguous stereocentres in 32–98% yields and 31–71% ee. The reaction evolved through a regioselective silylcupration of the alkyne-tethered cyclohexadienone in the presence of PhMe_2_SiBPin and the copper catalyst, leading to an alkenylsilyl copper intermediate which further reacted with the cyclohexadienone according to an enantioselective Michael addition. The catalyst system was compatible with a variety of substituents (R^1^) on the cyclohexadienone. In addition to terminal alkyne-tethered cyclohexadienones (R^2^ = H), an internal alkyne-tethered substrate (R^2^ = Me) also underwent the reaction albeit with both reduced yield (32%) and ee value (31% ee).

**Scheme 52:**
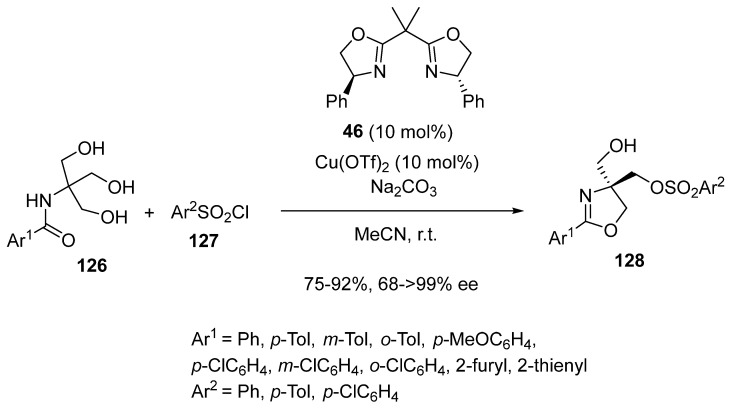
Domino sulfonylation/cyclisation reaction of aminotriols with aryl sulfonyl chlorides [70].

In 2020, an enantioselective copper-catalysed domino sulfonylation/cyclisation reaction between aminotriols **126** and aryl sulfonyl chlorides **127** was reported by Onomura et al. [70]. The catalyst system consisted in 10 mol% of a combination of chiral bisoxazoline ligand **46** and Cu(OTf)_2_ in acetonitrile. The process carried out at room temperature allowed the synthesis of chiral functionalised oxazolines **128** exhibiting a quaternary stereocentre (Scheme 52) in 75–92% yields and 68- > 99% ee. The domino reaction involved successively a mono-sulfonylation of aminotriols, an intramolecular cyclisation to afford prochiral oxazoline diols, and sulfonylative asymmetric desymmetrisation of the latter.

**Scheme 53:**
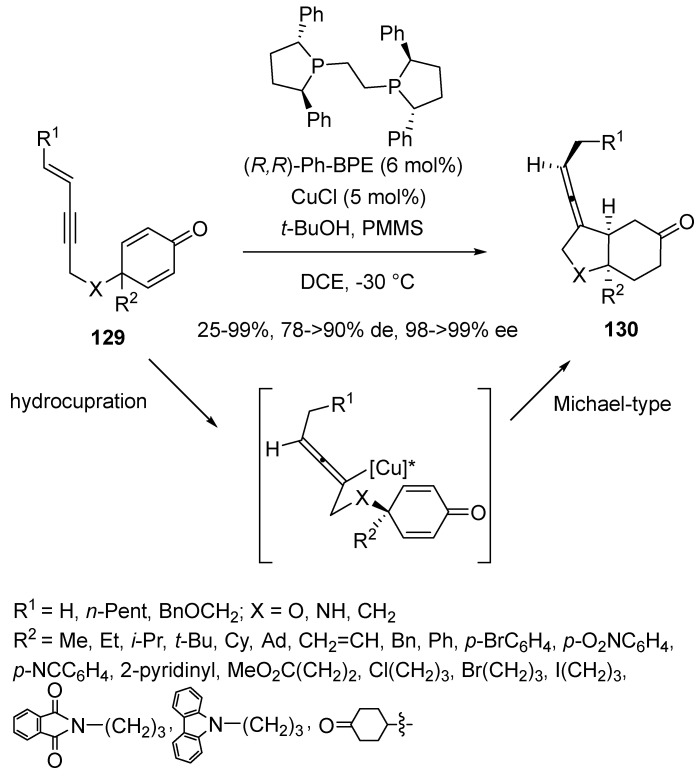
Domino hydrocupration/Michael-type reaction of 1,3-enyne-tethered cyclohexadienones [71].

In another area, Lin, Tian, and Gao developed in 2020 an enantioselective copper-catalysed intramolecular reductive coupling of 1,3-enynes to cyclohexadienones **129** which resulted in the formation of chiral trisubstituted exocyclic allenes **130** (Scheme 53) [71]. Performed in DCE at −30 °C under argon atmosphere in the presence of 5 mol% of CuCl combined with 6 mol% of chiral biphosphine ligand (*R*,*R*)-Ph-BPE, the domino hydrocupration/Michael-type reaction took place efficiently in the presence of a diversity of functional groups, thus producing a wide range of enantiopure (98- > 99% ee) exocyclic allenic products **130,** which included *cis*-hydrobenzofuran, *cis*-hydroindole, and *cis*-hydroindene moieties with 25–99% yields and 78- > 90% de.

**Scheme 54:**
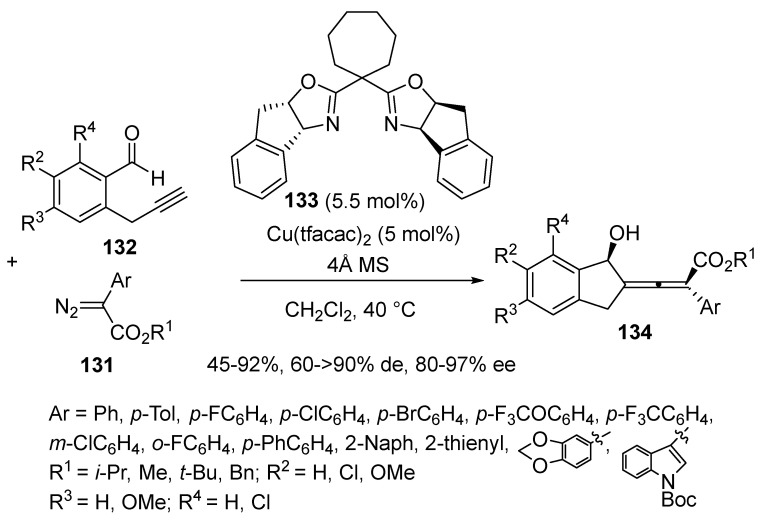
Domino coupling/aldol reaction of terminal alkynes with α-aryl diazoacetates [72].

In 2021, Sun et al. reported a synthesis of chiral exocyclic α-allenols arising from an enantioselective domino coupling/aldol reaction between α-aryl diazoacetates **131** and terminal alkynes **132** (Scheme 54) [72]. It was based on the use at 40 °C of a chiral catalyst in situ generated from 5 mol% of Cu(tfacac)_2_ and 5.5 mol% of chiral bisoxazoline ligand **133** in dichloromethane under argon atmosphere. This resulted in the formation of a range of chiral exocyclic α-allenols **134** with 45–92% yields, 60- > 90% de, and 80–97% ee. A diversity of either electron-donating or electron-withdrawing substituents was tolerated on the phenyl ring of the α-aryl diazoacetates, leading to the corresponding exocyclic α-allenols as almost single diastereomers (>90% de) with 55–92% yield, and 87–96% ee. α-Heteroaryl diazoacetates were also compatible.

**Scheme 55:**
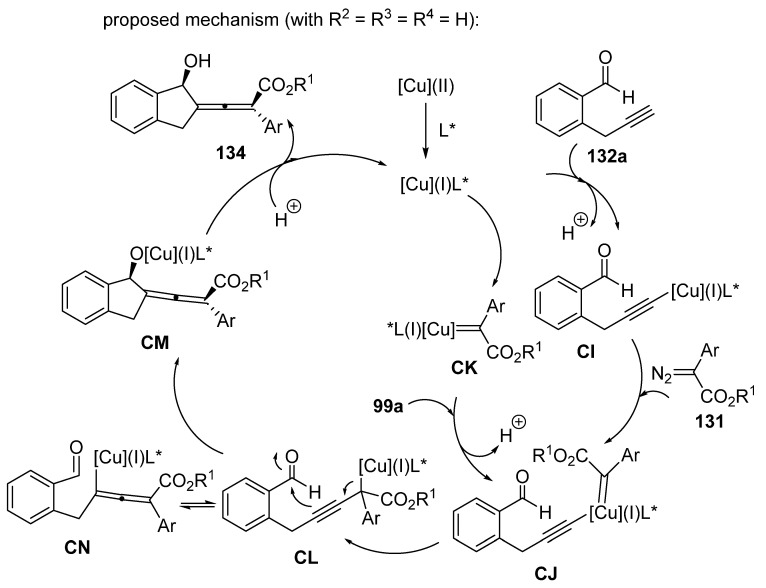
Mechanism for domino coupling/aldol reaction of terminal alkynes with α-aryl diazoacetates [72].

The authors proposed the mechanism detailed in Scheme 55, beginning with the formation of copper intermediate **CJ** from the coupling between copper acetylide **CI** and copper-carbene **CK**. Then, intermediate **CJ** underwent an alkynyl migratory insertion which produced intermediate **CL**. Subsequently, the alkynylogous aldol reaction of **CL** yielded allenoate copper species **CM**. Alternatively, the 1,3-copper migration of **CL** could form allenoate–copper intermediate **CN**, which was trapped by the aldehyde moiety to also generate **CM**. The final protonation of **CM** delivered the desired chiral exocyclic α-allenol along with regenerated catalyst.

**Scheme 56:**
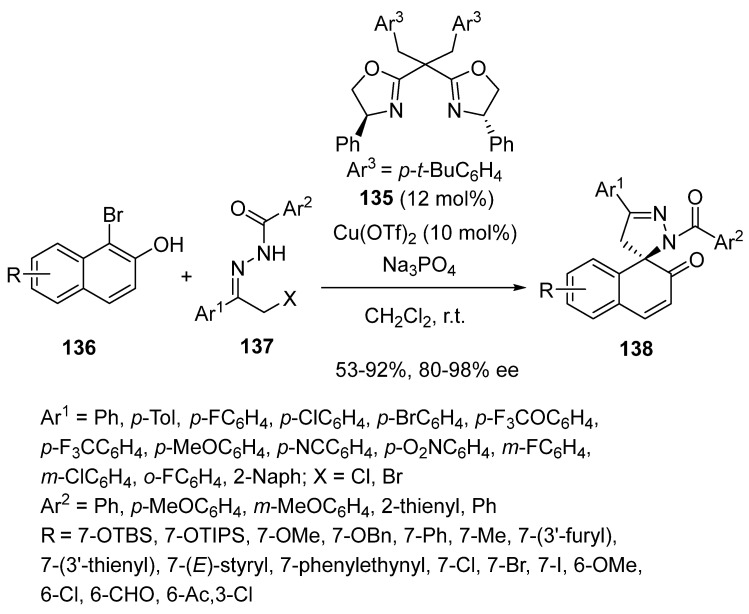
Domino electrophilic dearomatisation/S_RN_1-debromination/radical coupling reaction of α-bromo-β-naphthols with azoalkenes [73].

In 2022, a chiral copper catalyst derived from Cu(OTf)_2_ (10 mol%) and chiral bisoxazoline ligand **135** (12 mol%) was applied by Luan and Liu at room temperature in dichloromethane as the solvent to promote a formal asymmetric [4 + 1] spiroannulation reaction between α-bromo-β-naphthols **136** and azoalkenes **137** (Scheme 56) [73]. Actually, it dealt with an enantioselective domino electrophilic dearomatisation/S_RN_1-debromination/radical coupling reaction, allowing a direct access to chiral pyrazoline-based spirocyclic products **138** with 53–92% yields and 80–98% ee. The α-bromo-β-naphthols could bear different substituents on the phenyl ring as well as that of the aryl group (Ar^1^) of the azoalkene.

**Scheme 57:**
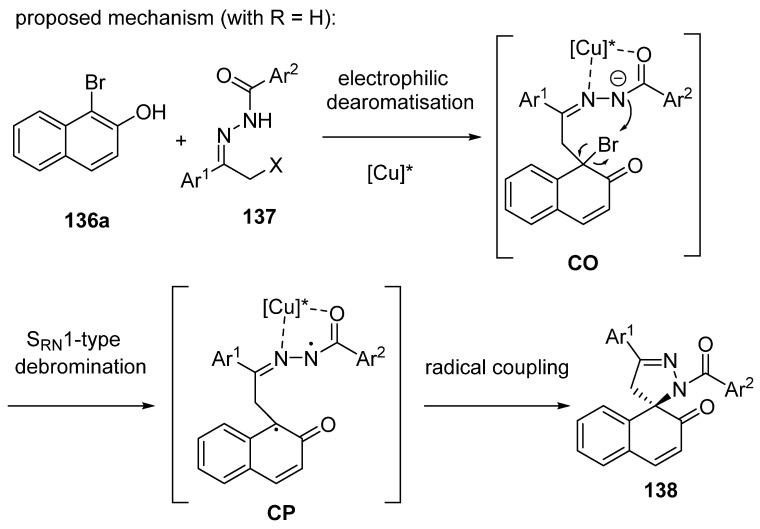
Mechanism for domino electrophilic dearomatisation/S_RN_1-debromination/radical coupling reaction of α-bromo-β-naphthols with azoalkenes [73].

Mechanistic studies (Scheme 57) demonstrated that the cascade was triggered by an electrophilic dearomatisation of the α-bromo-β-naphthol to give intermediate **CO**. The latter subsequently underwent debromination via S_RN_1-substitution within situ-formed *N*-nucleophile **CP**. The chiral copper(II)-species, bound with the azoalkene moiety, was assumed to control the enantiodiscrimination over the naphthoxy C-radical in **CP**. Finally, a radical coupling in **CP** delivered the product.

**Scheme 58:**
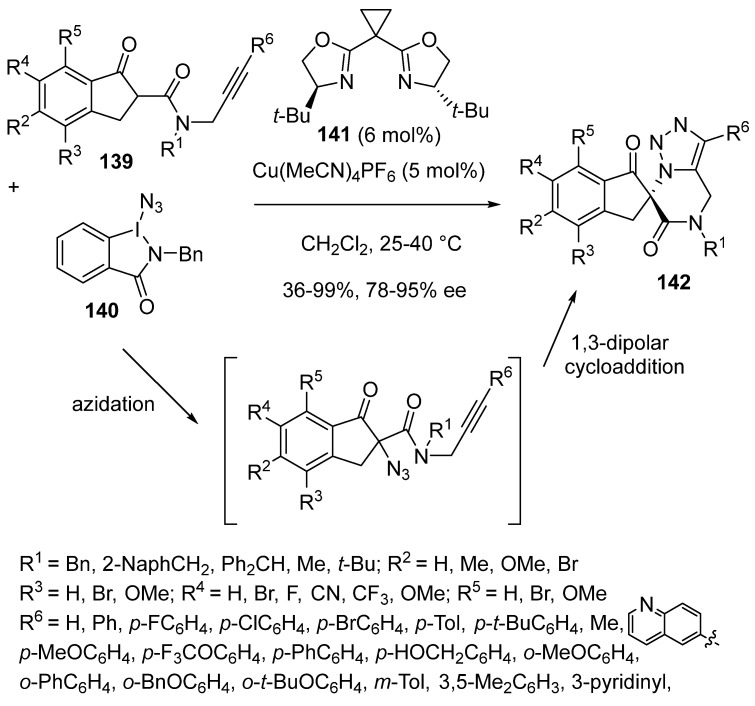
Domino azidation/1,3-dipolar cycloaddition reaction of *N*-propargyl-β-ketoamides with azidobenziodazolone [74].

A chiral bisoxazoline ligand was applied in 2024 by Cheng and Deng to access chiral triazoles, especially chiral 1,5-disubstituted triazoles (Scheme 58) [74]. The synthesis was based on an enantioselective domino azidation/1,3-dipolar cycloaddition reaction between *N*-propargyl-β-ketoamides **139** and azidobenziodazolone **140** as the azide source. The process employed 6 mol% of ligand **141** combined with 5 mol% of Cu(MeCN)_4_PF_6_ as the copper source in dichloromethane, thus affording at 25–40 °C a wide range of chiral triazoles **142** with 36–99% yields and 78–95% ee.

#### 2.9.2. Multicatalysis

**Scheme 59:**
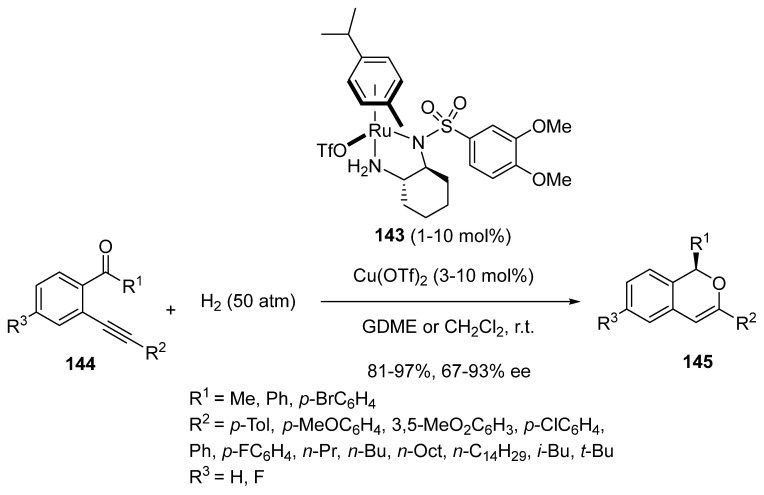
Domino cyclisation/hydrogenation reaction of *ortho*-(alkynyl)aryl ketones [75].

A cooperative Cu/Ru catalysis was applied by Yu and Fan in 2017 to develop the first asymmetric hydrogenation of in situ generated isochromenylium derivatives [75]. The binary catalyst system was constituted by 3–10 mol% of Cu(OTf)_2_ and 1–10 mol% of chiral cationic ruthenium–diamine complex **143**. The reaction was performed at room temperature in ethylene glycol dimethyl ether (GDME) or dichloromethane as solvent under H_2_ atmosphere (50 atm). As shown in Scheme 59, a broad range of *ortho*-(alkynyl)aryl ketones **144** were converted under these conditions into the corresponding chiral 1H-isochromenes **145** with high yields (81–97%) and moderate to excellent ee values (67–93% ee). These products could be further easily transformed into isochromanes, constituting an important structural motif in many biologically active/natural compounds.

**Scheme 60:**
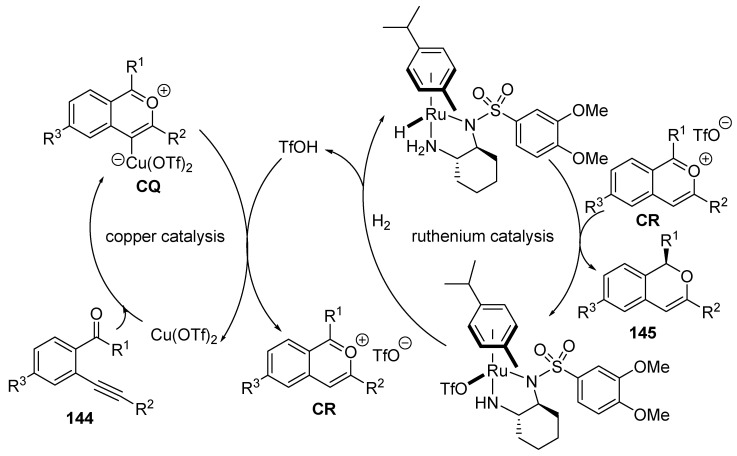
Mechanism for domino cyclisation/hydrogenation reaction of *ortho*-(alkynyl)aryl ketones [75].

The domino cyclisation/hydrogenation reaction evolved through the mechanism detailed in Scheme 60, beginning with the reaction of the *ortho*-(alkynyl)aryl ketone with Cu(OTf)_2_ to generate isobenzopyrylium salt **CQ**. Meanwhile, the chiral ruthenium–diamine catalyst reacted with H_2_ to produce a Ru—H complex and TfOH. Then, protonolysis of the C—Cu bond of intermediate **CQ** by TfOH regenerated Cu(OTf)_2_ and produced isobenzopyrylium intermediate **CR**. A final hydride transfer from the Ru—H complex to isobenzopyrylium intermediate **CR** delivered the product along with regenerated ruthenium catalyst.

**Scheme 61:**
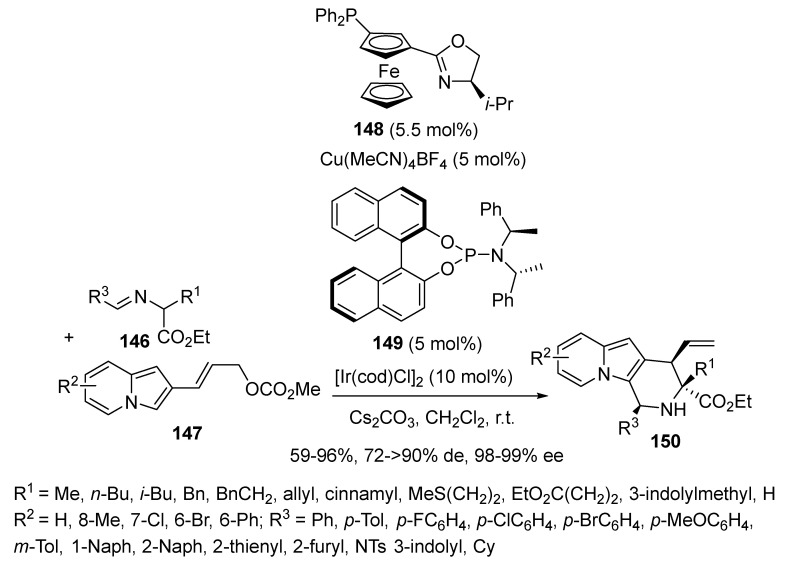
Domino allylic alkylation/Friedel–Crafts reaction of aldimine esters with 2-indolizinyl allyl carbonates [76].

In 2023, Wang, Dong and Dang described the first example of asymmetric synthesis of indolizines based on an enantioselective domino allylic alkylation/Friedel–Crafts reaction evolving through a synergistic Cu/Ir catalysis (Scheme 61) [76]. This remarkable process occurred at room temperature in dichloromethane between aldimine esters **146** and 2-indolizinyl allyl carbonates **147** in the presence of a combination of chiral copper catalyst in situ formed from 5 mol% of Cu(MeCN)_4_BF_4_ and 5.5 mol% of chiral oxazoline ligand **148**, and a chiral iridium catalyst in situ produced from 10 mol% of [Ir(cod)Cl]_2_ and 5 mol% of chiral phosphoramidite ligand **149**. The reaction allowed under this cooperative Cu/Ir catalysis an impressive number of enantiopure (98–99% ee) 2,3-fused indolizine derivatives **150** to be synthesised as single diastereomers in almost all cases of substrates studied (72- > 90% de) with good to excellent yields (59–96%). These products exhibited four stereogenic centres and the scope of this novel methodology was found remarkably broad especially toward the aldimine esters. According to the importance of indolizines in medicinal chemistry, the synthetic utility of this novel protocol was demonstrated by synthetic transformations of the domino products into other important chiral indolizine derivatives.

**Scheme 62:**
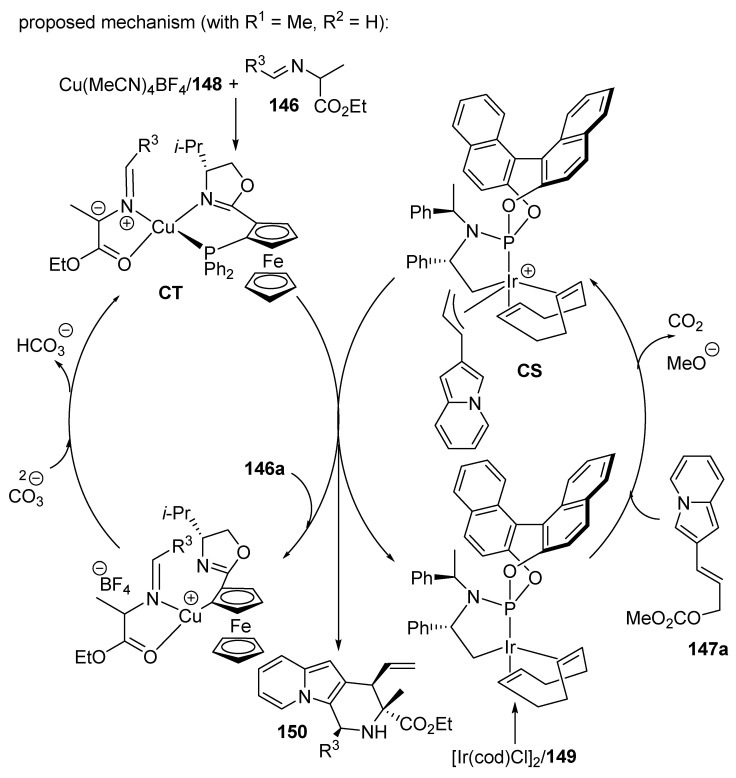
Mechanism for domino allylic alkylation/Friedel–Crafts reaction of aldimine esters with 2-indolizinyl allyl carbonates [76].

A cooperative mechanism is depicted in Scheme 62, proceeding through the asymmetric allylation between π-allyl-Ir(III) species **CS** derived from the 2-indolizine allyl carbonate and Cu(I)-azo-methine ylide **CT**, followed by a proton-assisted stereoselective Friedel–Crafts type cyclisation, delivering the final product.

**Scheme 63:**
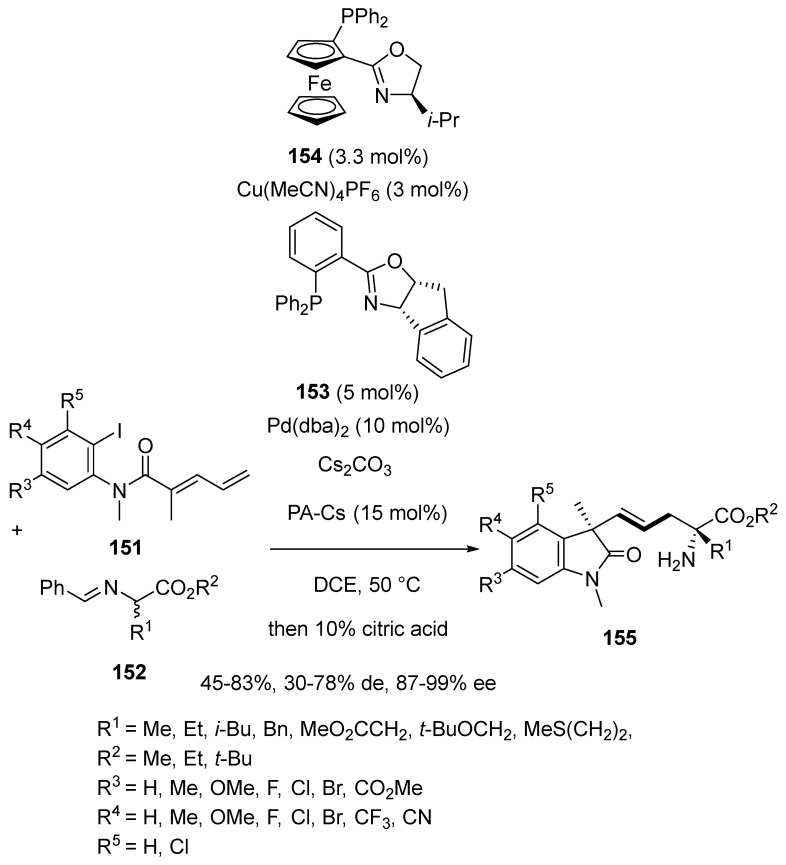
Domino Heck/nucleophilic trapping reaction of 2-iodophenyl-tethered dienamides with α-amino acid-derived aldimine esters [77].

Another dual catalysis was applied in 2024 by Huo and Zhang to develop enantioselective Heck-initiated domino reactions of 2-iodophenyl-tethered dienamides **151** with α-amino acid-derived aldimine esters **152** (Scheme 63) [77]. When these substrates were submitted at 50 °C in DCE to a combination of a chiral palladium catalyst, in situ generated from 10 mol% of Pd(dba)_2_ and 11 mol% of chiral phosphine **153**, and a chiral copper catalyst, in situ formed from 3 mol% of Cu(MeCN)_4_PF_6_ and 3.3 mol% of chiral ferrocenyl phosphine **154**, they underwent an asymmetric domino Heck/nucleophilic trapping reaction to give chiral products **155** exhibiting two privileged scaffolds that are oxindoles and non-natural α-amino acids. The reaction required the presence of Cs_2_CO_3_ as base and racemic phosphoric acid cesium (PA-Cs) as an additive. It enabled the regio-, diastereo- and enantioselective construction of 1,5-nonadjacent tetrasubstituted stereocentres through Pd/Cu-cocatalysed Heck-initiated domino reaction.

**Scheme 64:**
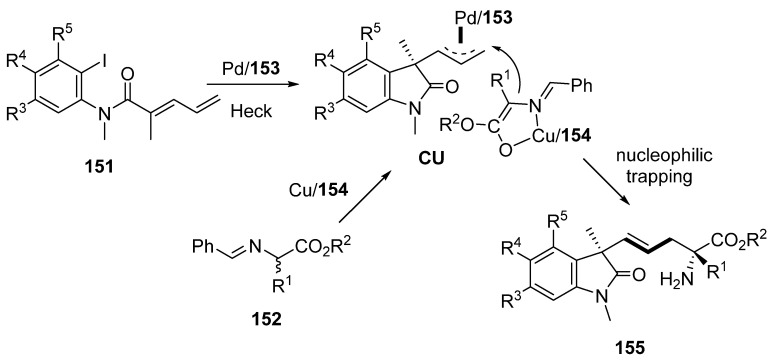
Mechanism for domino Heck/nucleophilic trapping reaction of 2-iodophenyl-tethered dienamides with α-amino acid-derived aldimine esters [77].

The reaction started by an asymmetric Heck reaction of the 2-iodophenyl-tethered dienamide, leading through intramolecular carbopalladation of the 1,1-disubstituted alkene to give π-allyl palladium intermediate **CU**. As illustrated in Scheme 64, the two chiral metal catalysts facilitated the attack of the nucleophilic α-amino acid-derived aldimine ester at the less-hindered terminal position of the π-allyl palladium intermediate **CU**, which afforded the products with 45–83% yields, 30–78% de, and 87–99% ee.

**Scheme 65:**
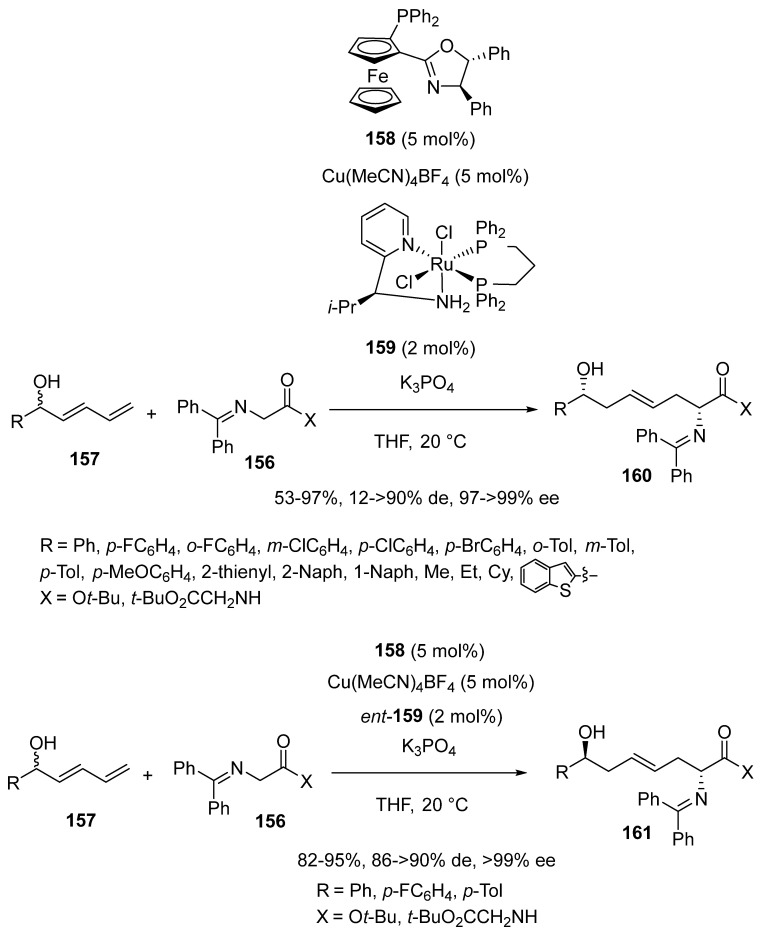
Domino dehydrogenation/1,6-Michael/hydrogenation reactions of diphenyl ketimine esters/peptides with branched dienyl carbinols [78].

In 2024, Dong and Wang developed unprecedented enantioselective copper/ruthenium relay-catalysed domino dehydrogenation/1,6-Michael addition/hydrogenation reactions occurring between diphenyl ketimine esters/peptides **156** and racemic branched dienyl carbinols **157** (Scheme 65) [78]. The process required the involvement of 5 mol% of a chiral copper catalyst derived from Cu(MeCN)_4_BF_4_ and phosphine ligand **158** combined with 2 mol% of chiral ruthenium catalyst **159** as bimetallic relay catalyst system. Performed at 20 °C in THF in the presence of K_3_PO_4_ as base, the domino process afforded chiral functionalised ζ-hydroxy amino ester/amide derivatives **160** exhibiting two 1,6-nonadjacent stereocentres and a unique β,γ-unsaturation moiety. A wide range of aromatic alcohols were compatible, providing the corresponding products **160** as almost single diastereomers (84- > 90% de) in 75–97% yields and >9% ee whatever the positions and electronic properties of the substituents exhibited on the aryl ring of the dienyl carbinols. Interestingly, even challenging aliphatic alcohols (R = Me, Et, Cy) reacted smoothly to give the desired products in good yields (70–80%) and excellent enantioselectivities (91- > 99% ee) albeit combined with low diastereoselectivities (0–26% de). Using the enantiomeric ruthenium catalyst *ent-***159** associated to the same chiral copper catalyst under the same conditions, allowed the synthesis of enantiopure (>99% ee) *trans-*diastereomers **161** to be achieved with 82–95% yields, 86- > 90% de, as illustrated in Scheme 65. This result constituted the first example of a stereodivergent synthesis of chiral products exhibiting remote 1,6-nonadjacent stereocentres.

## 3. Multicomponent Domino Reactions

### 3.1. Michael-Initiated Multicomponent Reactions

**Scheme 66:**
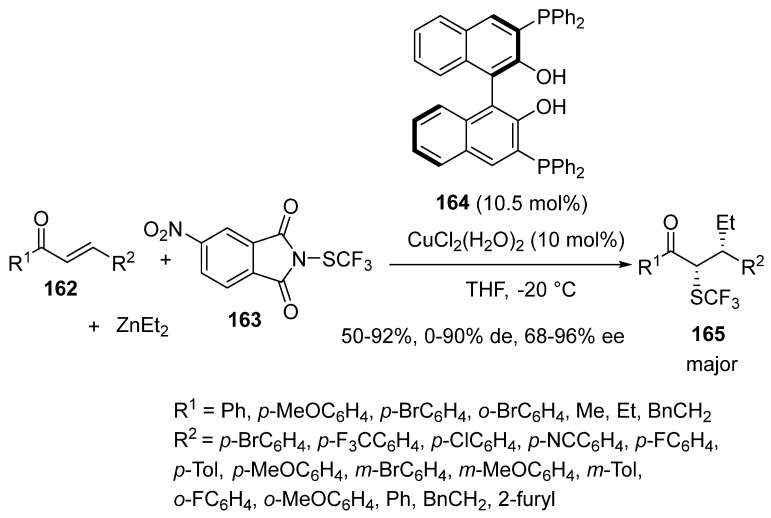
Three-component domino Michael/trifluoromethylthiolation reaction of α,β-unsaturated ketones, SCF_3_-4-nitro-phthalimide, and ZnEt_2_ [79].

Asymmetric trifluoromethylthiolation of carbonyl compounds remains underdeveloped. To fill this gap, Wang et al. described in 2018 the first example of enantioselective α-trifluoromethylthiolation of simple α,β-unsaturated ketones on the basis of a copper-catalysed three-component domino Michael/trifluoromethylthiolation of acyclic α,β-unsaturated ketones **162** with SCF_3–_4-nitro-phthalimide **163** and ZnEt_2_ (Scheme 66) [79]. The process involved a chiral catalyst in situ generated from 10 mol% of CuCl_2_(H_2_O)_2_ and 10.5 mol% of chiral BINOL-derived biphosphine ligand **164** employed at −20 °C in THF as the solvent. It allowed the asymmetric integration of a SCF_3_ group into carbonyl compounds **162**, thus affording α-SCF_3_-β-substituted carbonyl compounds **165** as major *syn*-diastereomers with 50–92% yields, variable diastereoselectivities (0–90% de), and moderate to excellent ee values (68–96% ee).

**Scheme 67:**
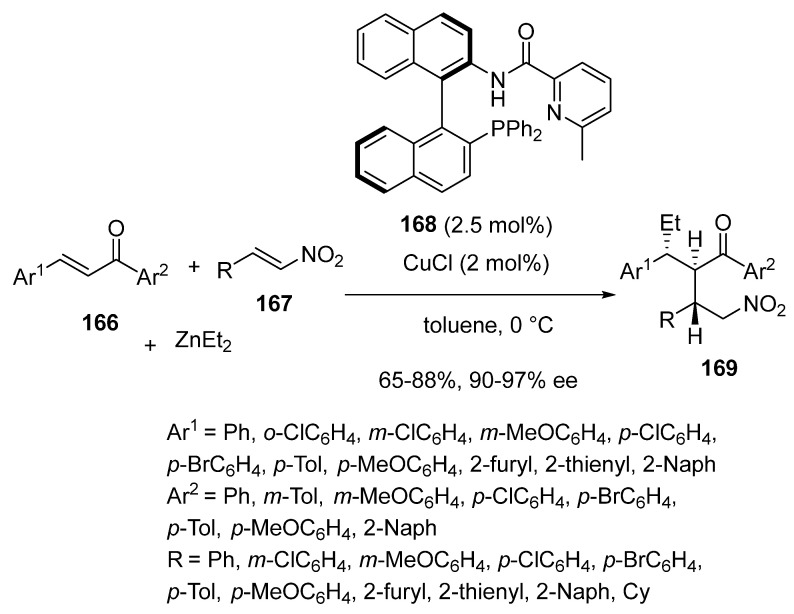
Three-component domino double Michael reaction of α,β-unsaturated ketones, nitroolefins and ZnEt_2_ [80].

An enantioselective three-component domino double Michael reaction of α,β-unsaturated ketones **166** with nitroolefins **167**, and ZnEt_2_ was described in 2019 Hu and Hou (Scheme 67) [80]. It employed a chiral copper catalyst in situ formed from 2 mol% of CuCl and 2.5 mol% of chiral *p*,*N*-ligand **168**. Carried out at 0 °C in toluene, the process resulted in the formation of functionalised chiral products **169** exhibiting three contiguous stereocentres as single diastereomers with 65–88% yields and 90–97% ee. It evolved through double Michael addition of ZnEt_2_ to α,β-unsaturated ketones followed by trapping with nitroolefins. It was found that the steric and electronic properties of the aromatic substituents of both chalcones and nitroolefins had low impact on the enantioselectivity of the reaction. Substrates with either electron-withdrawing or electron-donating substituents at any position of the phenyl ring of chalcones and aromatic nitroolefins were compatible, thereby affording the corresponding products with good yields and excellent enantioselectivities. Notably, an aliphatic nitroolefin (R = Cy) was also tolerated, resulting in the formation of the corresponding product with 60% yield and 94% ee.

**Scheme 68:**
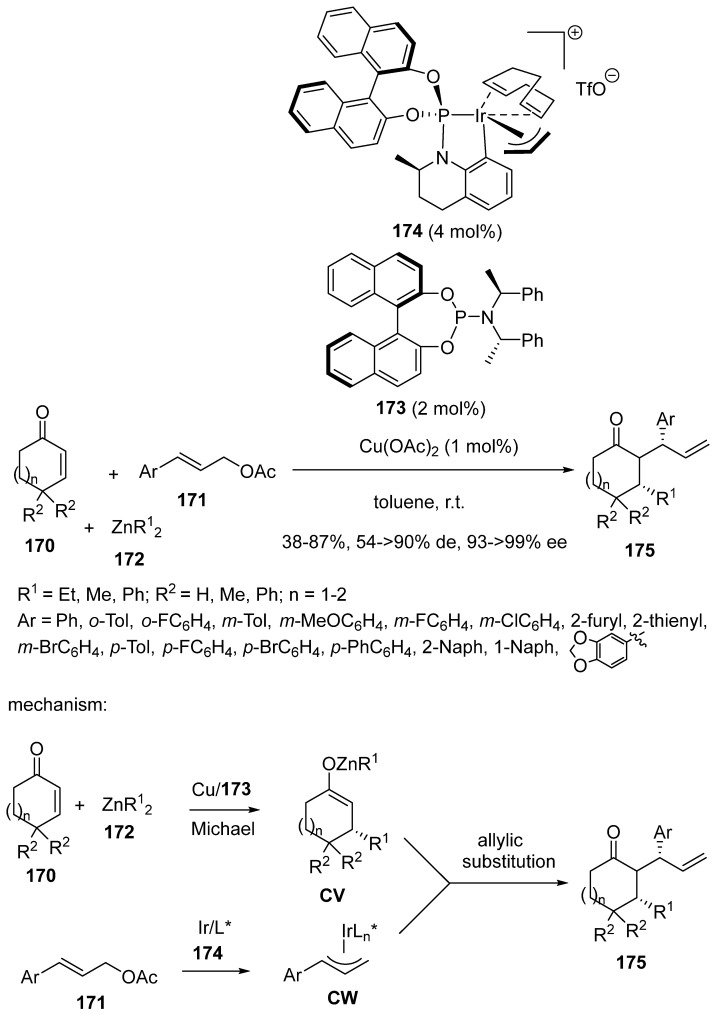
Three-component domino Michael/allylic substitution reaction of cyclic enones, allylic acetates and dialkylzinc reagents [81].

In 2023, You et al. developed an enantioselective three-component reaction between cyclic enones **170**, allylic acetates **171**, and dialkylzinc reagents **172** on the basis of a dual copper/iridium catalysis [81]. Indeed, a chiral copper catalyst in situ generated from 1 mol% of Cu(OAc)_2_ and 2 mol% of chiral phosphoramidite ligand **173** was involved to promote the first step of the domino process, namely the asymmetric conjugate addition of the dialkylzinc reagent **172** to the cyclic enone **170** which led to the corresponding chiral zinc enolate **CV**. In the same time, 4 mol% of chiral iridium catalyst **174** reacted with the allylic acetate **171** to give the π-allyl-Ir species **CW**. Then, the latter underwent an allylic alkylation reaction with enolate **CV** to deliver the final domino product **175** (Scheme 68). Performed in toluene at room temperature, the reaction allowed the synthesis of a range of enantiopure multisubstituted cyclic ketones **175** all obtained with both remarkable diastereo- (>90% de) and enantioselectivities (>99% ee) combined with moderate to high yields (38–87%). Lower diastereo- (54% de) and enantioselectivities (93% ee) were observed in only one case, that of the reaction involving ZnPh_2_ (R^1^ = Ph).

### 3.2. Photoredox-Catalysed Multicomponent Reactions

**Scheme 69:**
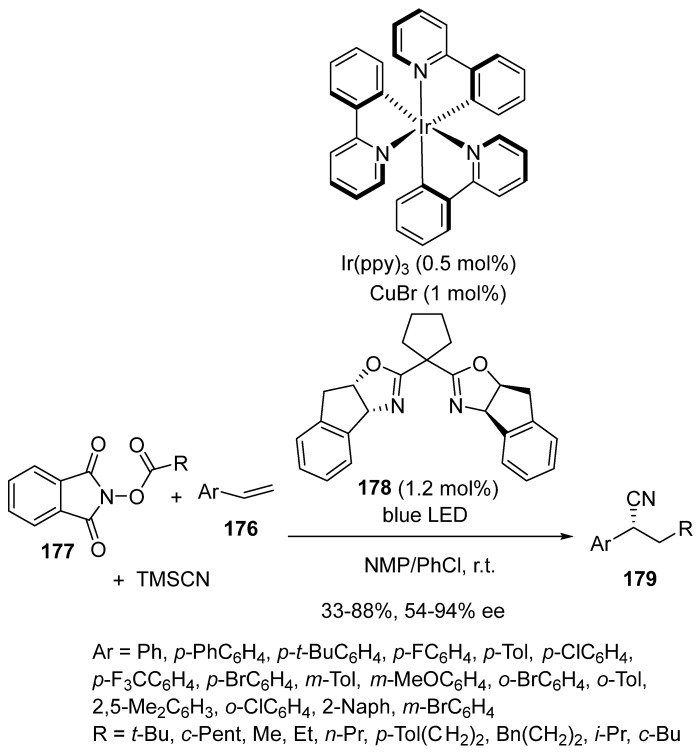
Three-component cyanoalkylation reaction of styrenes, alkyl *N*-hydroxyphthalimide esters and TMSCN [90].

The merger of photoredox catalysis with transition-metal catalysis, termed metallaphotoredox catalysis by MacMillan [82], has received considerable attention in both photochemistry and organometallic chemistry in the last decade [83,84,85,86,87,88]. Especially, merging photocatalysts with copper catalysts represents a green dual catalytic system for different reactions, spanning from cross-couplings to domino reactions performed under mild reaction conditions [89]. As an example, Han et al. developed in 2018 enantioselective domino cyanation/alkylation of aromatic alkenes **176** with TMSCN and alkyl *N*-hydroxyphthalimide esters **177** as alkylating agents [90]. As shown in Scheme 69, the three-component process involved as catalyst system 0.5 mol% of Ir(ppy)_3_ as the photocatalyst combined with a chiral copper catalyst in situ generated from 1 mol% of CuBr and 1.2 mol% of chiral bisoxazoline ligand **178**. Performed at room temperature in a mixture of chlorobenzene and *N-*methyl-2-pyrrolidone (NMP) as the solvent under blue LED irradiation, the reaction led to chiral products **179** with 33–88% yields and 54–94% ee. The catalyst system tolerated a wide scope since various styrenes bearing either electron-donating or electron-withdrawing groups on the phenyl ring were compatible. Especially, *para*-phenyl-substituted styrene reacted smoothly to give the desired product with 88% yield and 92% ee. Moreover, good results were achieved with both *meta*- and *ortho-*substituted styrenes. For example, the reaction of bulky *ortho*-bromostyrene provided the highest enantioselectivity (94% ee). Concerning the alkyl *N*-hydroxyphthalimide ester partners, they could include primary, secondary as well as tertiary alkyl groups.

**Scheme 70:**
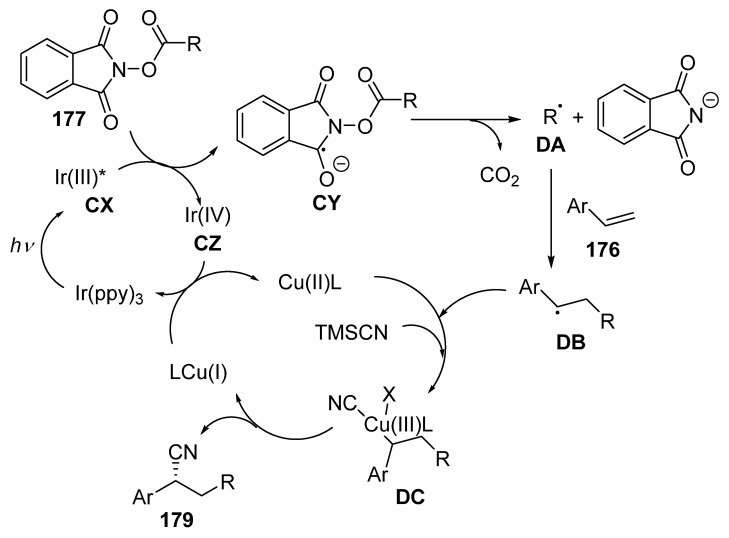
Mechanism for three-component cyanoalkylation reaction of styrenes, alkyl *N*-hydroxyphthalimide esters and TMSCN [90].

A possible mechanism for this cyanoalkylation reaction is depicted in Scheme 70. In a first time, the photoredox catalyst Ir(ppy)_3_ was irradiated into activated species **CX**, from which the alkyl *N*-hydroxyphthalimide ester abstracted an electron to give radical anion **CY** and the photocatalyst intermediate **CZ**. Then, radical anion **CY** generated alkyl radical **DA** and the phthalimide anion through releasing CO_2_. Subsequently, the addition of alkyl radical **DA** to the styrene afforded benzylic radical **DB**. Concomitantly, intermediate **CZ** oxidised a copper(I) catalyst to release photocatalyst Ir(ppy)_3_ along with formation of chiral Cu(II) catalyst. Then, the latter reacted with benzylic radical **DB** and TMSCN to provide chiral Cu(III) intermediate **DC**. Finally, the reductive elimination of **DC** delivered the product and regenerated the Cu(I) catalyst.

**Scheme 71:**
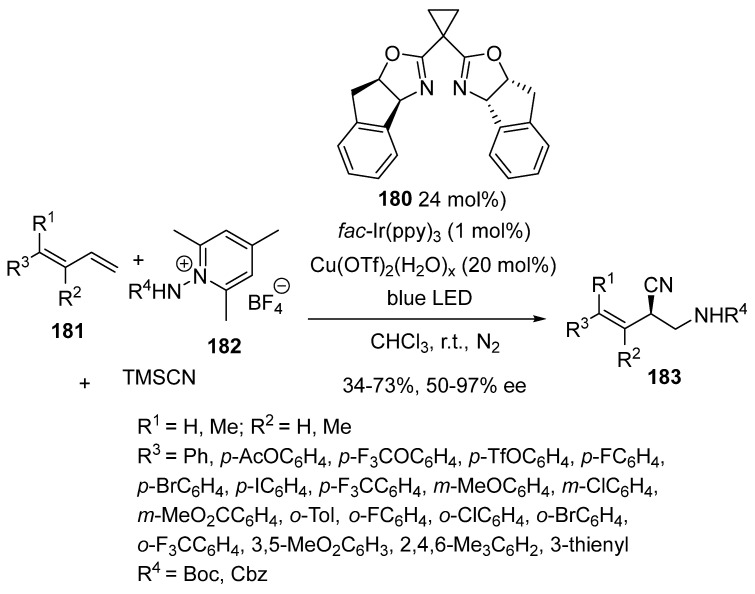
Three-component amidocyanation reaction of 1,3-dienes, TMSCN and *N*-protected-amidopyridinium salts [91].

While difunctionalisation of simple alkenes has been widely investigated on the basis of dual photoredox/metal catalysis, that involving 1,3-dienes remains underdeveloped. A rare example was reported in 2023 by Zhu et al., using the synergistic action of *fac-*Ir(ppy)_3_ (1 mol%) as the photoredox catalyst and a chiral copper catalyst in situ generated from 20 mol% of Cu(OTf)_2_(H_2_O)_x_ and 24 mol% of chiral bisoxazoline ligand **180** [91]. As illustrated in Scheme 71, this catalyst system was successfully applied to promote under N_2_ atmosphere enantioselective 1,2-amidocyanation of 1,3-dienes **181** with TMSCN and *N*-Boc-amidopyridinium salts **182**. Under blue LED irradiation at room temperature, the three-component reaction carried out in chloroform afforded chiral products **183** with moderate to good yields (34–73%) and moderate to excellent enantioselectivities (50–97% ee). A range of aryl-substituted 1,3-dienes (R^3^ = aryl) was tolerated with the presence of electron-withdrawing groups or electron-donating groups at any positions of the phenyl ring. Even sterically hindered 2,4,6-trimethyl-phenyl-substituted diene (R^3^ = 2,4,6-Me_3_C_6_H_2_) underwent the difunctionalisation to produce the desired product with 54% yield and 87% ee. An heteroaromatic-substituted substrate (R^3^ = 3-thienyl) was also compatible (43% yield, 93% ee). Notably, alkyl-substituted 1,3-dienes (R^3^ = alkyl) also reacted smoothly albeit with both moderate yields (34–49%) and ee values (50–62% ee). In addition to Boc-protected amidopyridinium salts, a *N*-Cbz-amidopyridinium salt led to the corresponding product with 49% yield and 93% ee.

**Scheme 72:**
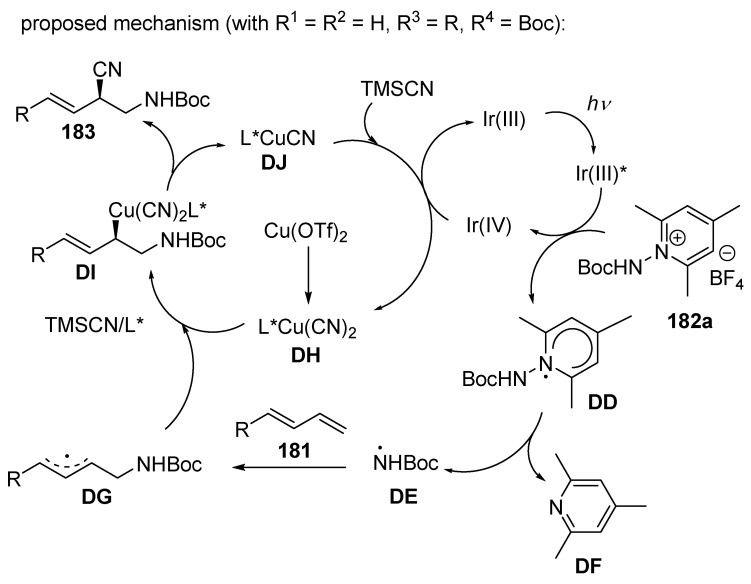
Mechanism for three-component amidocyanation reaction of 1,3-dienes, TMSCN, and *N*-protected-amidopyridinium salts [91].

The mechanism shown in Scheme 72 involved the reduction of the *N*-amidopyridinium salt into radical **DD** by excited *fac*-Ir(III)*, which subsequently underwent fragmentation to give radical **De, and** 2,4,6-collidine **DF**. Regioselective addition of nitrogen-centered radical **DE** to the 1,3-diene substrate generated allylic radical **DG**. The latter species was then converted into allylic Cu(III) complex **DI** in the presence of L*Cu(CN)_2_ (**DH**). Intermediate **DI** further underwent reductive elimination, which produced the final product, along with L*CuCN (**DJ**). Subsequent oxidation of **DJ** into **DH** in the presence of TMSCN by Ir(IV) closed the catalytic cycle, regenerating both the Ir(III) and the Cu(II) species **DH**.

### 3.3. Multicomponent Reactions Initiated by Hydrocupration

**Scheme 73:**
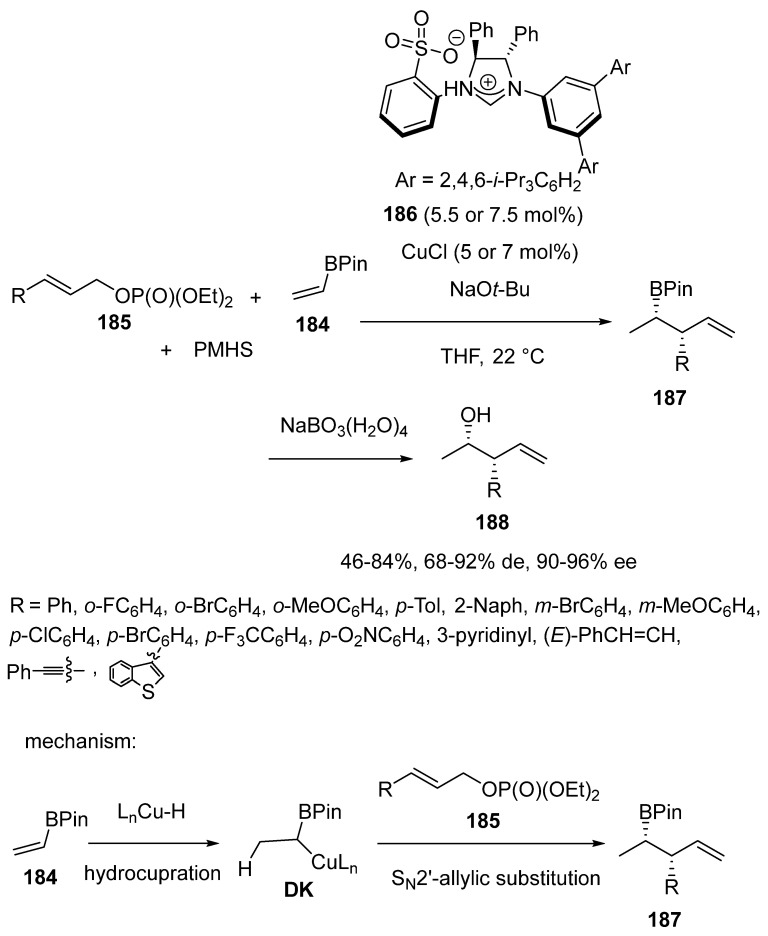
Three-component domino hydrocupration/S_N_2′-allylic substitution reaction of PMHS, vinyl-B(Pin) and (*E*)-1,2-disubstituted allylic phosphates [92].

In 2017, Hoveyda et al. disclosed an enantioselective three-component reaction between a silyl hydride, such as polymethylhydrosiloxane (PMHS), commercially available vinyl-B(Pin) **184**, and (*E*)-1,2-disubstituted allylic phosphates **185** (Scheme 73) [92]. A copper catalyst based on chiral sulfonate-containing *N*-heterocyclic carbene ligand **186** (5.5 or 7.5 mol%) was used to promote this reaction at 22 °C in THF. It occurred through asymmetric hydrocupration of the vinylborane to give intermediate **DK**, followed by S_N_2′-selective allylic substitution with the 1,2-disubstituted allylic phosphate, which afforded borane products **187** as major *syn*-diastereomers. The latter was further oxidised by treatment with NaBO_3_‧4H_2_O to provide the corresponding chiral alcohols **188** with 46–84% yields, excellent ee values (90–96% ee), and moderate to high de values (68–92% de). The substrate scope was found large since a range of aryl-substituted allylic phosphates bearing different types of substituents at any position of the phenyl ring were well tolerated. Moreover, heteroaryl-, alkenyl-, alkynyl-, and alkyl-substituted allylic phosphates could be converted into the desired homoallylic alcohols albeit in the presence of 7.5 mol% of chiral ligand **186** associated to 7 mol% of CuCl, whereas the reaction of aryl-substituted allylic phosphates required only 5.5 mol% of **186** combined with 5 mol% of CuCl.

**Scheme 74:**
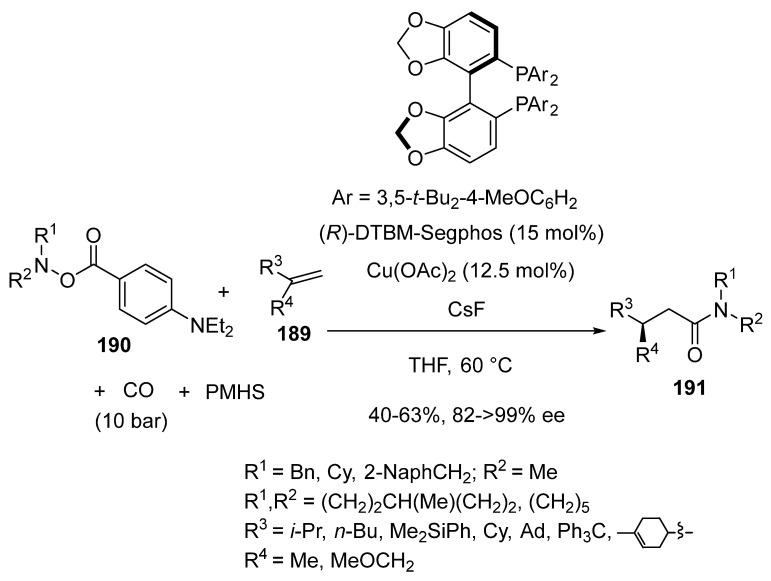
Four-component domino hydrocupration/aminocarbonylation reaction of 1,1-disubstituted alkenes, hydroxylamine esters, CO and PMHS [93].

Later in 2023, another hydrocupration of unactivated alkenes was incorporated by Wu et al. in an enantioselective copper-catalysed four-component reaction between 1,1-disubstituted alkenes **189**, hydroxylamine esters **190**, polymethylhydrosiloxane (PMHS) as the hydride source, and CO (10 bar) [93]. The process was carried out in THF at 60 °C in the presence of 12.5 mol% of Cu(OAc)_2_ as precatalyst and 15 mol% of biphosphine (*R)*-DTBM-Segphos as chiral ligand (Scheme 74). The hydrocupration of 1,1-disubstituted alkenes **189** proceeded preferentially to anti-Markovnikov selectivity, and was followed by aminocarbonylation to afford a range of chiral amides **191** with 40–63% yields and 82- > 99% ee. The catalyst system was compatible with variously substituted alkenes, providing the best levels of enantioselectivity when exhibiting steric bulky substituents.

### 3.4. Miscellaneous Multicomponent Reactions

**Scheme 75:**
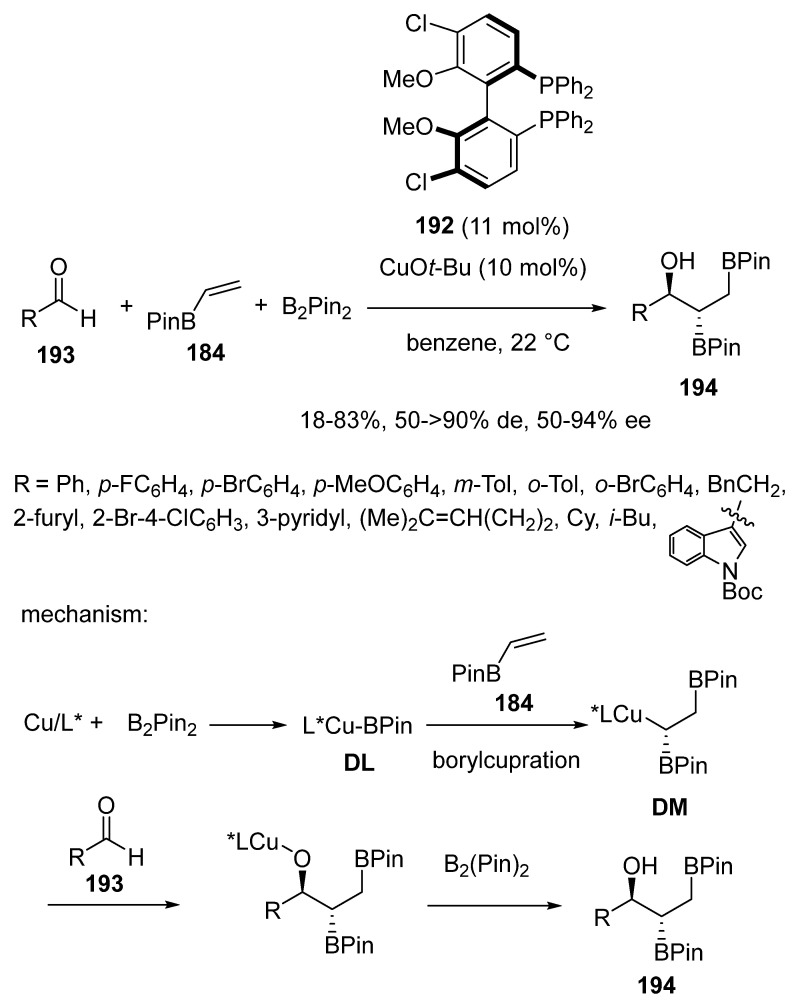
Three-component domino borylation/1,2-addition reaction of vinyl-B(Pin), B_2_Pin_2_, and aldehydes [94].

In 2017, Meek et al. employed 11 mol% of chiral biphosphine ligand **192** in combination with 10 mol% of CuO*t*-Bu as precatalyst to promote an enantioselective three-component reaction between vinyl-B(Pin) **184**, B_2_Pin_2_, and aldehydes **193** (Scheme 75) [94]. The reaction performed at 22 °C in benzene evolved through the borylcupration of the vinyl borane with (L*)Cu−B(Pin) species **DL**, arising from the reaction between B_2_Pin_2_ and the chiral copper complex, to give nucleophilic α,β-bisboryl-copper intermediate **DM**. The latter subsequently underwent 1,2-addition to aldehyde **193**, which produced the final chiral 1-hydroxy bis(boronate) ester **194** with 18–83% yields, 50- > 90% de, and 50–94% ee. The reaction conditions were tolerant with (hetero)aryl, vinyl, and alkyl aldehydes.

**Scheme 76:**
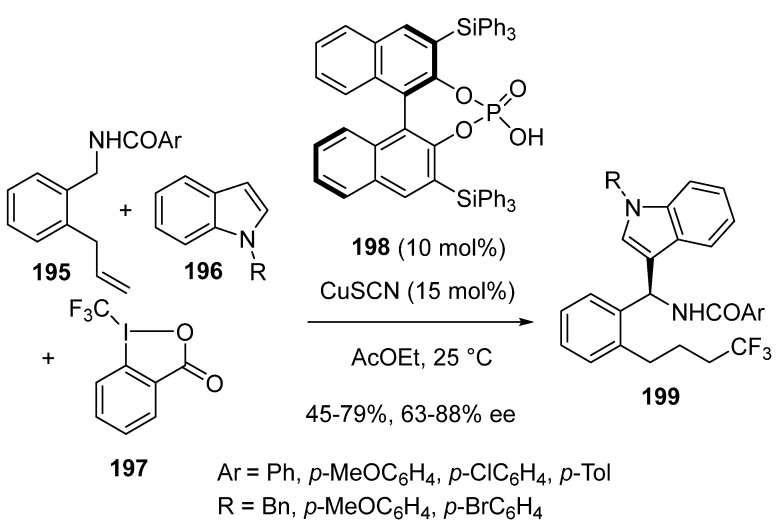
Three-component domino radical trifluoromethylation/1,5-shift/Friedel—Crafts alkylation reaction of *N*-(2-allylbenzyl)benzamides, *N-*protected indoles, and Togni’s reagent [95].

In the same year, a dual copper/Brønsted acid catalytic system was applied by Liu and Zhi to develop an enantioselective domino radical trifluoromethylation/1,5-shift/Friedel—Crafts alkylation reaction of *N*-(2-allylbenzyl)benzamide **195**, *N-*protected indole **196**, and commercially available Togni’s reagent **197** (Scheme 76) under argon atmosphere [95]. The process was carried out at 25 °C in ethyl acetate with 15 mol% of CuSCN and 10 mol% of chiral phosphoric acid **198**.

**Scheme 77:**
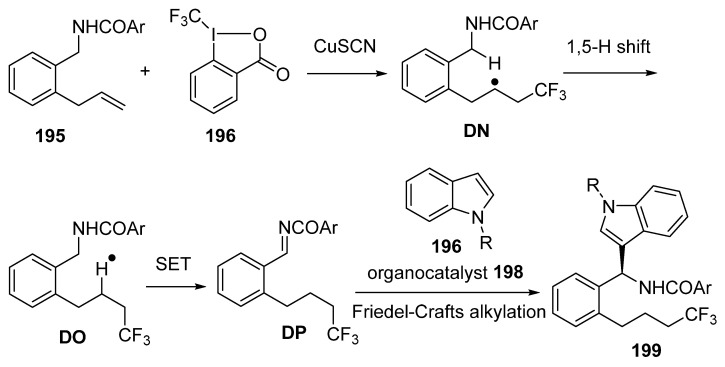
Mechanism for three-component domino radical trifluoromethylation/1,5-shift/Friedel—Crafts alkylation reaction of *N*-(2-allylbenzyl)benzamides, *N-*protected indoles, and Togni’s reagent [95].

As shown in Scheme 77, it began with a radical trifluoromethylation of the alkene moiety of *N*-(2-allylbenzyl)benzamide **195** by reaction with Togni’s reagent **197** to give radical intermediate **DN**. Then, the latter underwent an intramolecular 1,5-H shift which produced intermediate **DO** and then imine intermediate **DP** through single-electron oxidation. Subsequently, an enantioselective Friedel—Crafts alkylation of **DP** with indole **196** catalysed by the chiral Brønsted acid delivered final product **199**. This novel methodology based on mild and economic reaction conditions allowed broad range of useful chiral trifluoromethylated indole derivatives to be achieved with 45–79% yields and 63–88% ee.

**Scheme 78:**
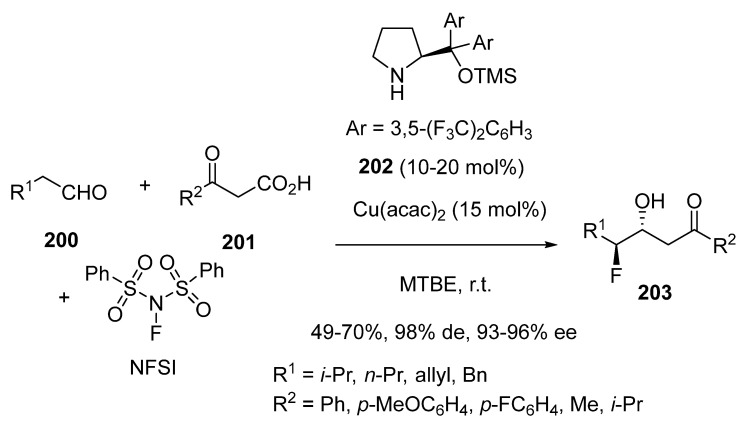
Three-component domino fluorination/aldol reaction of aliphatic enolisable aldehydes, keto acids, and NFSI [96].

Another dual catalysis was involved by Quintard and Rodriguez in 2017 in the development of enantioselective domino fluorination/aldol reactions between simple aliphatic enolisable aldehydes **200**, keto acids **201**, and an electrophilic fluorine source such as NFSI (Scheme 78) [96]. The catalyst system included 10–20 mol% of chiral diaryl-prolinol silyl ether **202** and 15 mol% of Cu(acac)_2_. The three-component process dealt with an organocatalysed fluorination followed by a copper-catalysed aldolisation. It was performed at room temperature in MTBE, leading to chiral vicinal fluorohydrins **203** as single diastereomers (98% de) with 49–70% yields and excellent enantioselectivities (93–96% ee).

**Scheme 79:**
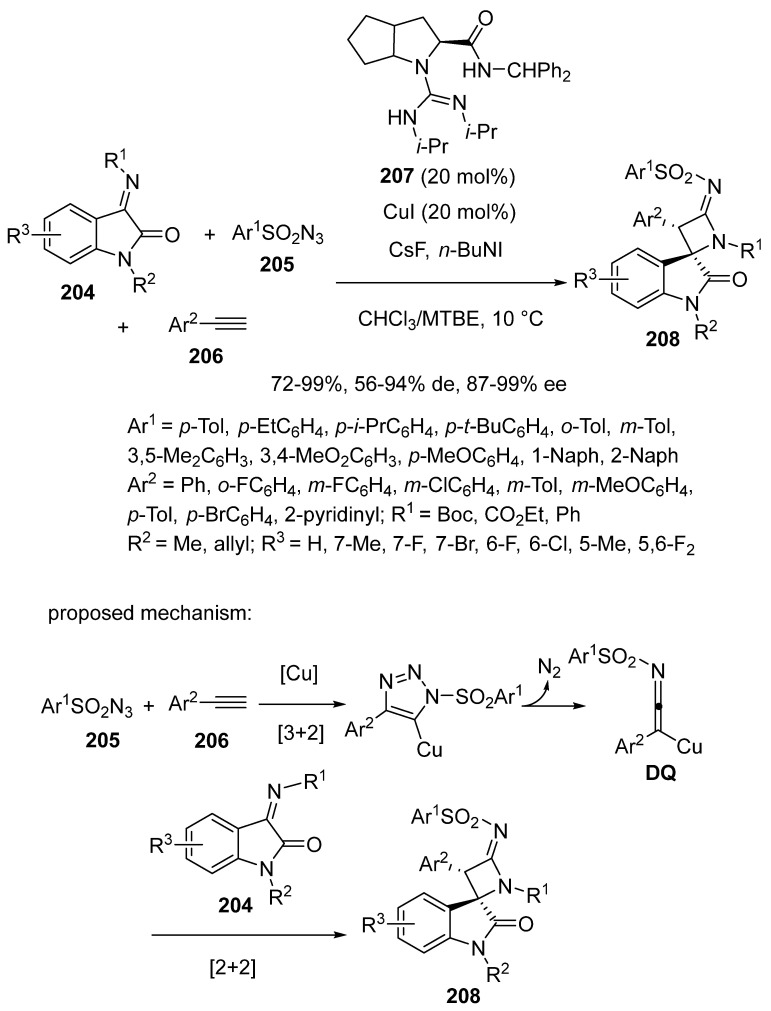
Three-component domino [3 + 2] cycloaddition/ring-opening/[2 + 2] cycloaddition reaction of isatin-derived ketimines, sulfonyl azides, and terminal alkynes [97].

In 2018, Liu et al. described an asymmetric three-component reaction between isatin-derived ketimines **204**, sulfonyl azides **205**, and terminal alkynes **206** (Scheme 79) [97]. It was carried out at 10 °C in a mixture of chloroform and MTBE as the solvent, employing 20 mol% of a chiral catalyst in situ prepared from CuI and chiral guanidine ligand **207**. It evolved through the formation of ketenimine species **DQ** arising from the [3 + 2] cycloaddition of sulfonyl azides **205** with terminal alkynes **206**, followed by ring-opening rearrangement upon the release of nitrogen. Then, intermediate **DQ** was trapped by isatin-derived ketimines **204** through a [2 + 2] cycloaddition to give a wide variety of chiral spiroazetidinimine oxindoles **208** with good to quantitative yields (72–99%), moderate to excellent diastereoselectivities (56–94% de) and uniformly high enantioselectivities (87–99% ee). This domino [3 + 2] cycloaddition/ring-opening/[2 + 2] cycloaddition reaction tolerated a wide variety of sulfonyl azides and terminal alkynes. It was found that the position and steric properties of substituents on the phenyl ring of the sulfonyl azides had no impact on the reactivity and stereoselectivities of the reaction. Furthermore, terminal aryl alkynes bearing halogen, methyl, or methoxy substituents at different positions of the phenyl group could be converted into the desired products with 92–98% ee. Unfortunately, the catalyst system did not tolerate alkyl-substituted alkynes.

**Scheme 80:**
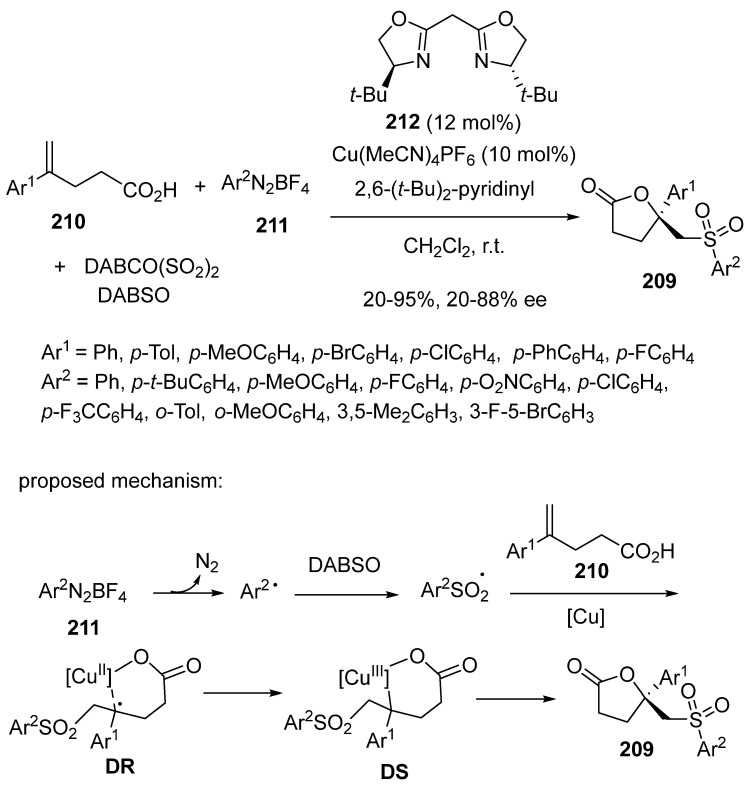
Three-component domino radical addition/cyclisation reaction of unsaturated carboxylic acids, aryldiazonium tetrafluoroborates and DABSO [98].

In another area, Han et al. disclosed an enantioselective domino radical addition/cyclisation reaction, allowing the synthesis of chiral sulfonyl lactones **209** (Scheme 80) [98]. The latter arose from the three-component reaction between unsaturated carboxylic acids **210**, aryldiazonium tetrafluoroborates **211** as the aryl radicals, and DABCO(SO_2_)_2_ (DABSO) as the sulfonyl precursor under argon atmosphere. The catalyst system was constituted by 10 mol% of Cu(MeCN)_4_BF_4_ and 12 mol% of chiral bisoxazoline ligand **212**. When performed at room temperature in dichloromethane as the solvent, a variety of fonctionalised lactones bearing a quaternary stereogenic centre were achieved with both moderate to high yields (20–95%) and enantioselectivities (20–88% ee). A mechanism is proposed in Scheme 80, beginning with the generation of aryl radical Ar^2^∙from the aryldiazonium tetrafluoroborate in the presence of Cu(I) catalyst. This aryl radical was then trapped by DABSO·to give the corresponding aryl sulfonyl radical Ar^2^SO_2_^∙^, which added to the unsaturated carboxylic acid in the presence of copper, thus generating Cu(II) intermediate **DR**. Subsequently, a single electron oxidation of **DR** occurred, producing chiral Cu(III) complex **DS**. A final reductive elimination of the latter species delivered the product.

**Scheme 81:**
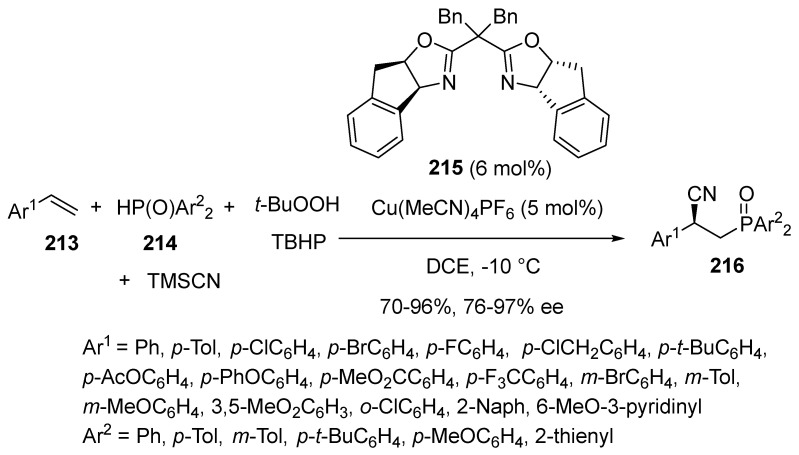
Four-component domino radical addition/cyanation reaction of styrenes, organophosphine oxides, TMSCN, and TBHP [99].

Always in the area of radical chemistry, Liu and Zou reported in 2019 the first copper-catalysed asymmetric phosphinocyanation of styrenes **213** with organophosphine oxides **214**, TMSCN, and *tert*-butyl hydrogen peroxide (TBHP) as the radical initiator. (Scheme 81) [99]. This four-component domino radical addition/cyanation reaction was performed under N_2_ atmosphere in the presence of a chiral catalyst in situ generated from 5 mol% of Cu(MeCN)_4_PF_6_ and 6 mol% of chiral bisoxazoline ligand **215** in DCE at −10 °C. This allowed the formation of a series of chiral β-cyanodiarylphosphine oxides **216** in 70–96 yields and 76–97% ee.

**Scheme 82:**
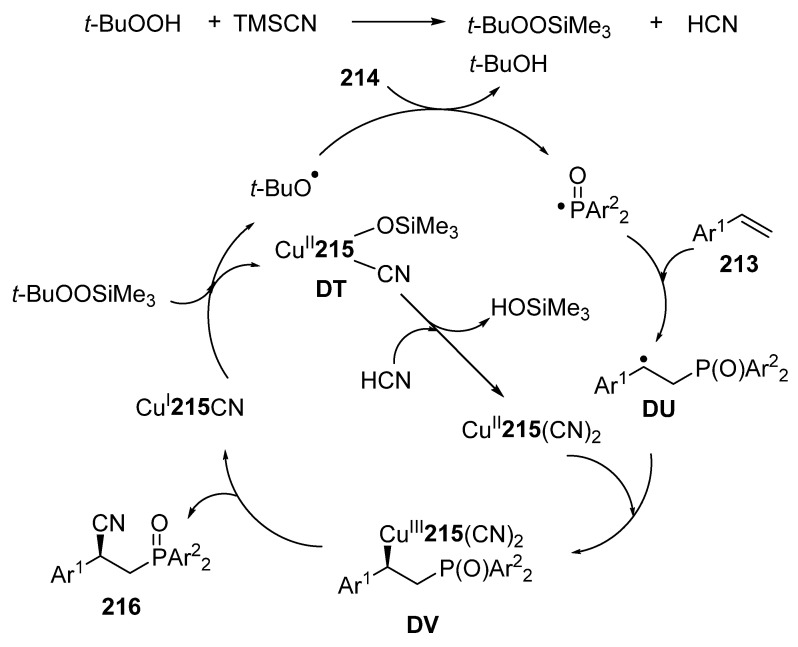
Mechanism for four-component domino radical addition/cyanation reaction of styrenes, organophosphine oxides, TMSCN and TBHP [99].

The authors proposed the mechanism shown in Scheme 82, beginning with the reaction of TBHP with TMSCN to generate the real reactive oxidant *t*-BuOOSiMe_3_ (TBPS) along with HCN. Then, *t*-BuOOSiMe_3_ coordinated to (**215**)Cu^I^(CN) to form Cu^II^ species **DT** along with *tert*-butoxy radical. Subsequently, **DT** reacted with HCN to yield active (**215**)Cu^II^(CN)_2_ species. Meanwhile, the *tert*-butoxy radical abstracted the hydrogen of Ar^2^_2_P(O)H to give phosphinoyl radical Ar^2^_2_P(O), which then added to the styrene to give benzylic radical species **DU**. The latter was subsequently enantioselectively trapped by (**215**)Cu^II^(CN)_2_ to give chiral intermediate **DV**, which delivered the final product.

**Scheme 83:**
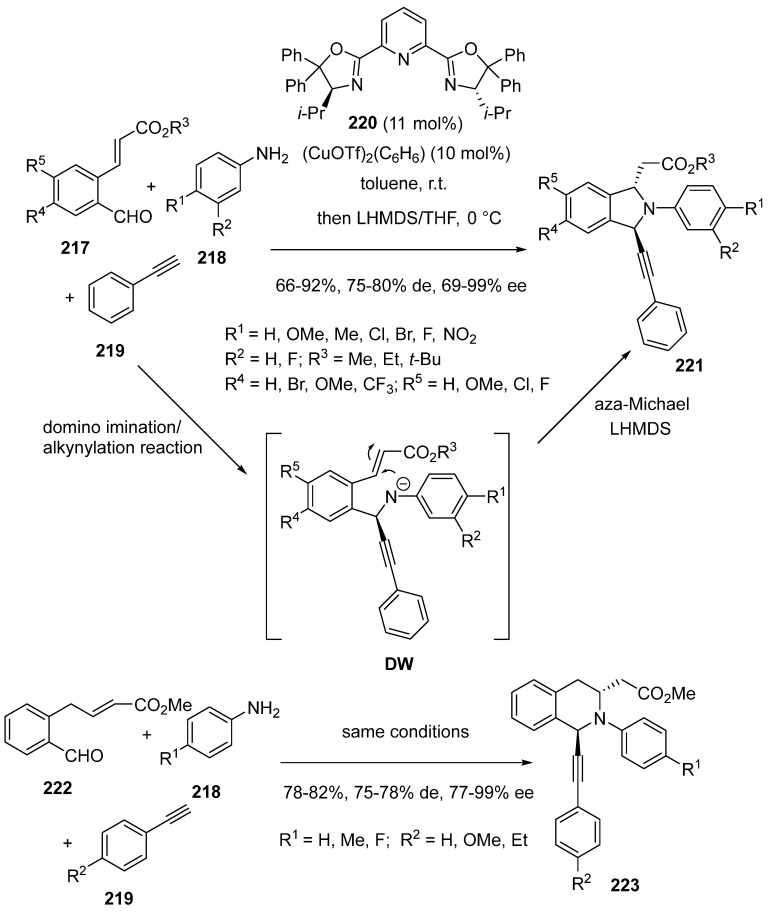
Three-component domino imination/alkynylation reactions followed by aza-Michael reaction of 2-formylphenyl acrylate/2-formylphenyl crotonate, anilines and phenyl acetylenes [100].

A number of biologically active products include chiral isoindolines and tetrahydroisoquinolines as skeletons, making their synthesis challenging. Despite this, only a few methodologies have been developed for their catalytic asymmetric synthesis. To fill this gap, Singh et al. reported in 2019 an asymmetric three-component reaction between 2-formylphenyl acrylate **217**, aniline **218**, and phenyl acetylene **219** (Scheme 83) [100]. It was achieved at room temperature in toluene under catalysis with a chiral copper catalyst derived from 10 mol% of (CuOTf)_2_∙C_6_H_6_ and 11 mol% of chiral Pybox ligand **220**. Under these conditions, the process evolved through a domino imination/alkynylation reaction which afforded intermediate **DW**, which was not isolated but directly submitted to an aza-Michael reaction in the presence of LHMDS as a base in THF at 0 °C, which allowed the cyclised final chiral 1,3-disubstituted isoindolines **221** to be obtained in one-pot with 66–92% yields, 75–80% de, and 69–99% ee. The scope of the methodology was extended to 2-formylphenyl crotonate **222** which, by reaction with anilines **218** and phenylacetylenes **219**, allowed the synthesis of chiral tetrahydroisoquinolines **223** with 78–82% yields, 75–78% de, and 77–99% ee, as illustrated in Scheme 83.

**Scheme 84:**
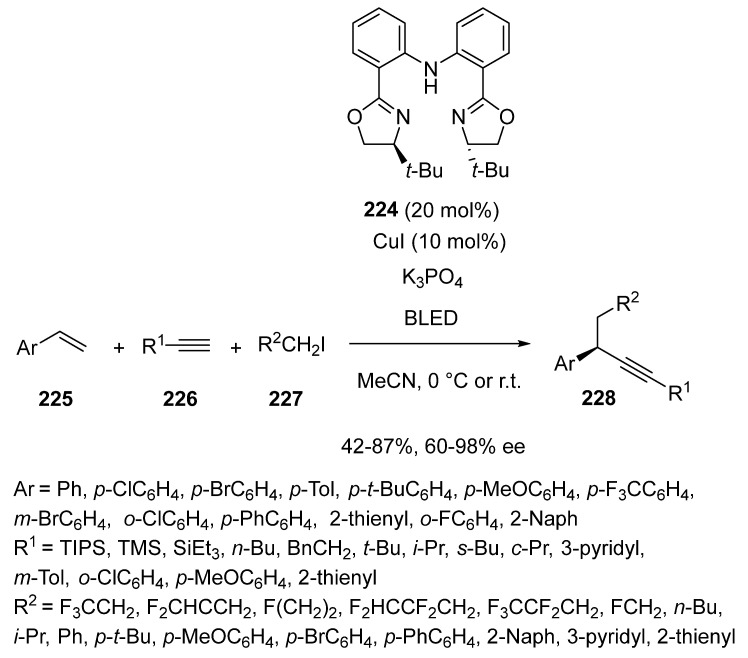
Three-component domino alkylation/alkynylation reaction of alkenes, terminal alkynes, and alkyl iodides [101].

In 2020, a copper catalyst in situ generated from chiral bisoxazoline ligand **224** (20 mol%) and CuI (10 mol%) was applied by Zhang and Zhang to promote an enantioselective photo-induced three-component domino alkylation/alkynylation reaction between styrenes **225**, terminal alkynes **226**, and alkyl iodides **227** [101]. Carried out in acetonitrile at room temperature or 0 °C, this constituted a novel simple methodology for asymmetric difunctionalisation of olefins (Scheme 84). It occurred under irradiation with blue light emitting diode (BLED), allowing chiral propargylic products **228** to be synthesised with 42–87% yields and 60–98% ee. A wide variety of substrates were compatible with generally the best enantioselectivities achieved in the reaction of silylated and aliphatic alkynes (R^1^ = Si(*i*-Pr)_3_, alkyl) while (hetero)aromatic alkynes reacted with lower ee values (60–84% ee). The styrene substrate tolerated substitutions at any position of the phenyl ring; however, the yields were decreased for the *ortho*-substituted components due to the steric hindrance. Furthermore, a diversity of alkyl iodides reacted smoothly including fluorinated ones.

**Scheme 85:**
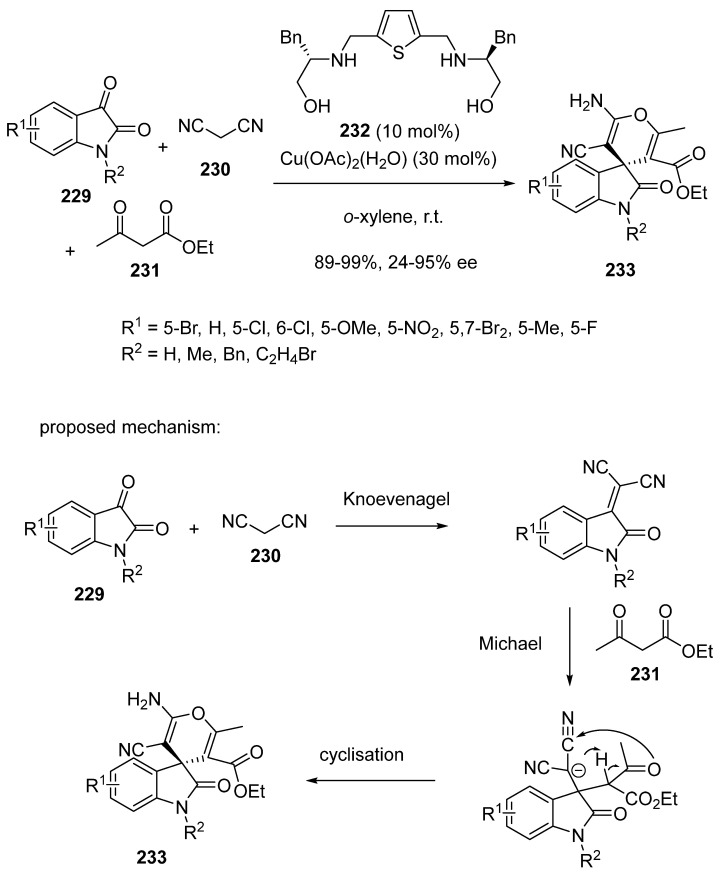
Three-component domino Knoevenagel/Michael/cyclisation reaction of isatins, ethyl acetoacetate, and malononitrile [102].

With the aim of synthesising biologically important chiral spirooxindoles, Islam and Al-Majid described in 2022 a novel enantioselective three-component reaction occurring between substituted isatins **229**, ethyl acetoacetate **231**, and malononitrile **230** (Scheme 85) [102]. This domino Knoevenagel/Michael/cyclisation reaction was carried out at 25 °C in THF in the presence of a chiral copper catalyst derived from 30 mol% of Cu(OAc)_2_(H_2_O) and 10 mol% of chiral thiophene-2,5-bis(amino-alcohol) ligand **232**, which led to desired chiral spirooxindoles **233** with 89–99% yields and 24–95% ee according the mechanism detailed in Scheme 85.

**Scheme 86:**
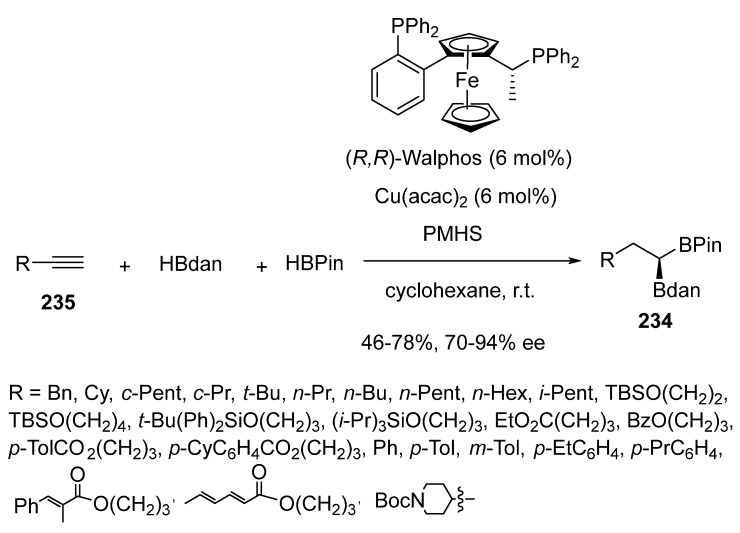
Three-component domino double hydroboration reaction of terminal alkynes with HBPin and HBdan [103].

In spite of the fact that chiral organoborons constitute key reagents in organic synthesis, methodologies for their synthesis remain rare and, consequently, challenging. In 2022, Yu and Song proposed a novel access to chiral 1,1-diborylalkanes **234** based on an enantioselective copper-catalysed double hydroboration of simple terminal alkynes **235** with two different boranes, such as pinacolborane (HBPin) and 1,8-diaminonaphthalatoborane (HBdan) [103]. The optimal catalyst arose from the combination of 6 mol% of Cu(acac)_2_ and the same quantity of ferrocenyl biphosphine ligand (*R*,*R*)-Walphos (Scheme 86). The three-component process was performed at room temperature in cyclohexane as the solvent in the presence of PMHS as an additive to afford chiral *gem*-bis(boryl) alkanes **234** with 46–78% yields and 70–94% ee. Unactivated aliphatic and aromatic alkynes were all compatible. Especially, an impressive series of aliphatic alkynes bearing various functionalities and different carbon chain length afforded the desired products in good yields and high levels of enantioselectivity.

**Scheme 87:**
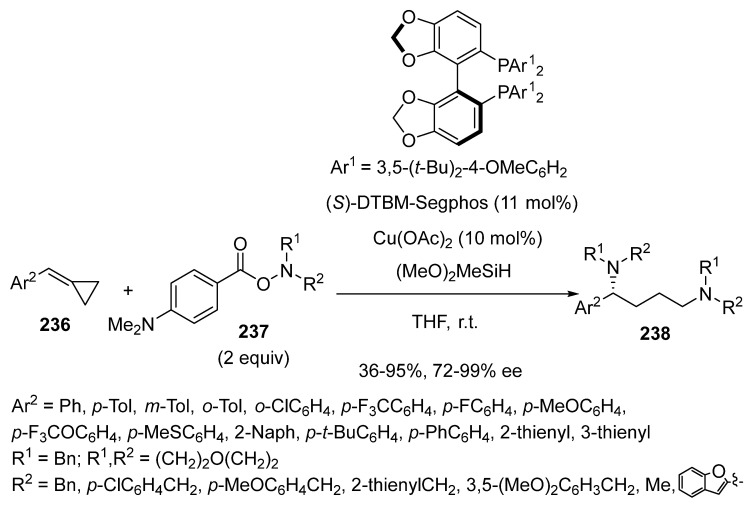
Pseudo-three-component domino hydroamination ring-opening/hydroamination reaction of methylene cyclopropanes with two equivalents of hydroxylamine esters [104].

Despite the importance of chiral 1,4-diamines in medicinal chemistry and their use as chiral ligands, the synthesis of these molecules remains challenging. In this context, Wang and Lee developed in 2022 an enantioselective copper-catalysed domino double hydroamination reaction between aryl methylene cyclopropanes **236** and two equivalents of hydroxylamine esters **237** to construct in THF at room temperature chiral 1,4-diamine derivatives **238** in moderate to high yields (36–95%) and generally high enantioselectivities (72–99% ee), as shown in Scheme 87 [104]. The optimal catalyst was in situ formed from 10 mol% of Cu(OAc)_2_ and 11 mol% of chiral biphosphine ligand (*S*)-DTBM-Segphos. Dimethoxymethylsilane was employed as a hydride source. Mechanistic studies demonstrated that this pseudo-three-component reaction evolved through an hydroamination ring-opening followed by a second hydroamination.

**Scheme 88:**
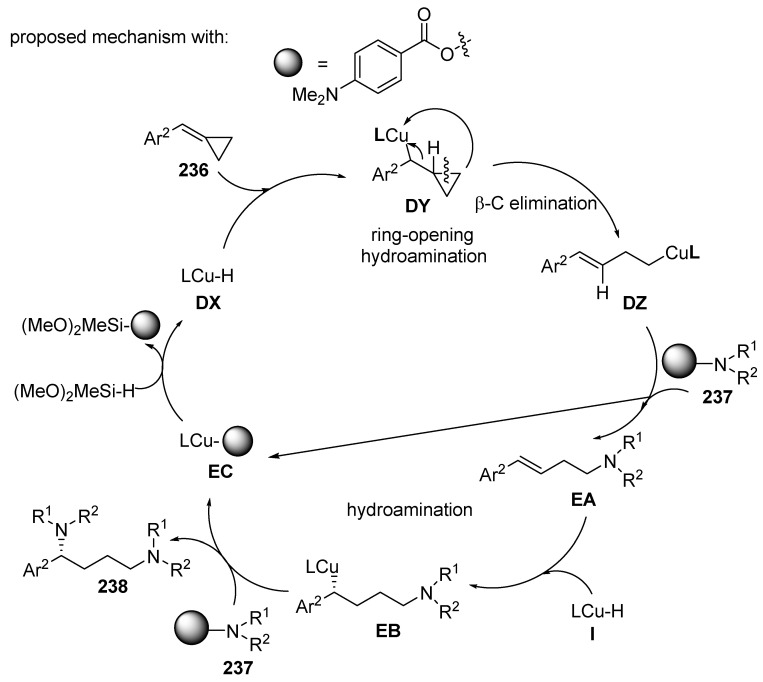
Mechanism for pseudo-three-component domino hydroamination ring-opening/hydroamination reaction of methylene cyclopropanes with two equivalents of hydroxylamine esters [104].

As detailed in the mechanism depicted in Scheme 88, copper intermediate **DY** was formed through the insertion of chiral L*Cu—H species **DX** into the double bond of the methylene cyclopropane. Then, the latter underwent a β-C elimination, which provided intermediate **DZ**. Then, the cleavage of the *N*—O bond of the hydroxyl–amine ester and subsequent reductive elimination generated intermediate **EA**. Subsequently, the latter underwent hydroamination to deliver the final product accompanied by a release of copper benzoate **EC,** which regenerated complex **DX** through transmetalation of the dimethoxymethylsilane.

**Scheme 89:**
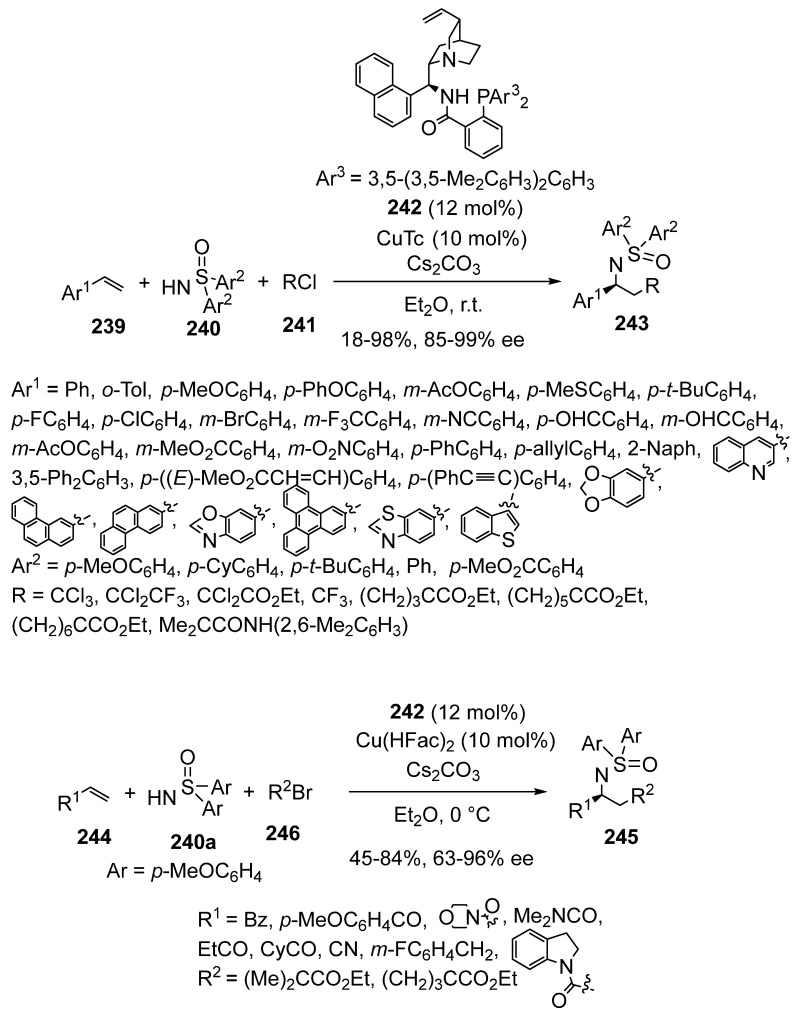
Three-component domino radical addition/amination reactions of alkenes, alkyl halides, and sulfoximines [105].

A chemo- and enantioselective copper-catalysed radical 1,2-carboamination of alkenes with simple alkyl halides and sulfoximines was disclosed in 2022 by Li, Liu, and Zhang in order to produce chiral amine scaffolds (Scheme 89) [105]. The process evolved through the radical addition of alkyl halides as radical precursors to alkenes followed by enantioselective radical C−N formation by reaction with sulfoximines. The three-component process between styrene-type alkenes **239**, sulfoximines **240**, and alkyl chlorides **241** employed at room temperature a combination of 10 mol% of CuTc and 12 mol% of chiral cinchona alkaloid-derived phosphine ligand **242** as catalyst system in the presence of Cs_2_CO_3_ as base in diethyl ether as solvent. It resulted in the formation of a wide range of chiral amines **243** in 18–98% yields and uniformly high ee values (85–99% ee). Along with styrene derivatives, carbonyl-substituted alkenes **244** were also compatible, leading to the corresponding chiral amines **245** with 45–84% yields and 63–96% ee by reaction with sulfoximine **240a**, and alkyl bromides **246** (Scheme 89). In this case, the reaction occurred at 0 °C in the presence of 10 mol% of Cu(HFac)_2_ as precatalyst combined with 12 mol% of the same ligand **242**. It must be noted that the scope of the reaction was remarkably wide, since it included alkenes bearing various electronic properties, such as aryl-, heteroaryl-, carbonyl-, and aminocarbonyl-substituted ones, along with diverse radical precursors, such as alkyl chlorides and bromides.

**Scheme 90:**
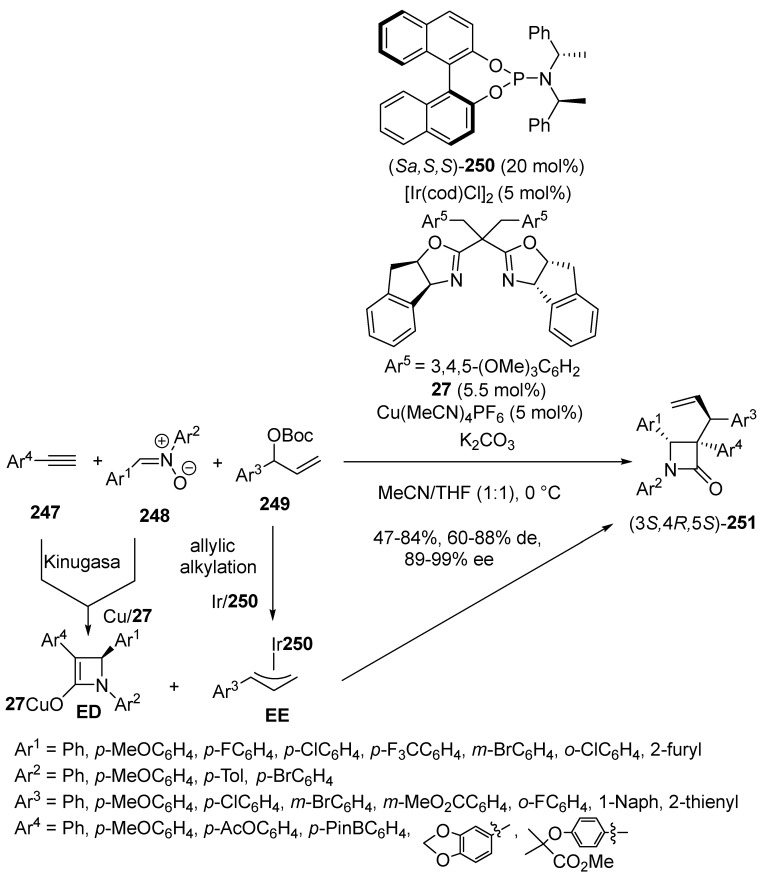
Three-component domino Kinugasa/allylic alkylation reaction of aromatic terminal alkynes, aromatic nitrones and aromatic allylic carbonates [106].

A cooperative dual Cu/Ir catalysis was applied by Xu et al. in 2023 to develop an enantioselective three-component domino Kinugasa/allylic alkylation reaction between terminal aromatic alkynes **247**, aromatic nitrones **248**, and aromatic allylic carbonates **249** (Scheme 90) [106]. The catalyst system included a chiral copper complex in situ generated from 5 mol% of Cu(MeCN)_4_PF_6_ and 5.5 mol% of chiral bisoxazoline ligand **27**, and a chiral iridium catalyst in situ formed from 5 mol% of [Ir(cod)Cl]_2_ and 20 mol% of chiral phosphoramidite ligand (*Sa*,*S*,*S*)-**250**. The former catalyst promoted the asymmetric Kinugasa reaction between the alkyne and the nitrone to give four-membered intermediate **ED**, and the iridium complex catalysed the second step of the domino process, e.g., the allylic alkylation of intermediate **ED** with reactive allyliridium intermediate **EE** arising from the allylic carbonate.

**Scheme 91:**
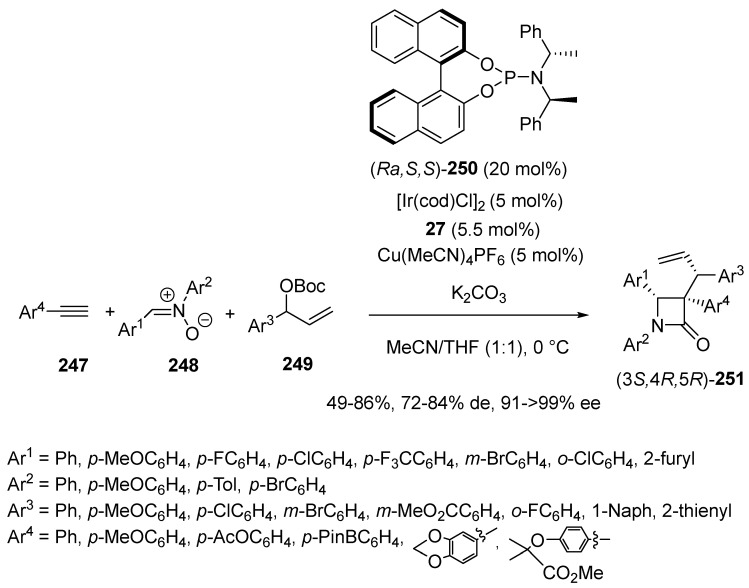
Three-component domino Kinugasa/allylic alkylation reaction of aromatic terminal alkynes, aromatic nitrones and aromatic allylic carbonates [106].

When this multicatalysed one-pot reaction was carried out at 0 °C in a 1:1 mixture of acetonitrile and THF as the solvent in the presence of K_2_CO_3_ as a base, it resulted in the formation of β-lactams (3*S*,4*R*,5*S*)-**251** exhibiting contiguous tertiary/quaternary/tertiary stereocentres with uniformly high ee values (89–99% ee), combined with both good to high yields (47–84%) and diastereoselectivities (60–88% de). The aromatic substituents of the three substrates could bear different types of substituents on the phenyl ring. Interestingly, the authors found that replacing phosphoramidite ligand (*Sa*,*S*,*S*)-**250** by enantiomeric ligand (*Ra*,*S*,*S*)-**250** under the same conditions allowed the stereodivergent access to diastereomeric lactams (3*S*,4*R*,5*R*)-**251**, with 49–86% yields, 72–84% de, and 91- > 99% ee, as illustrated in Scheme 91. Therefore, four of the eight possible stereoisomers could be achieved with using two different chiral iridium catalysts under similar conditions and from the same set of substrates.

**Scheme 92:**
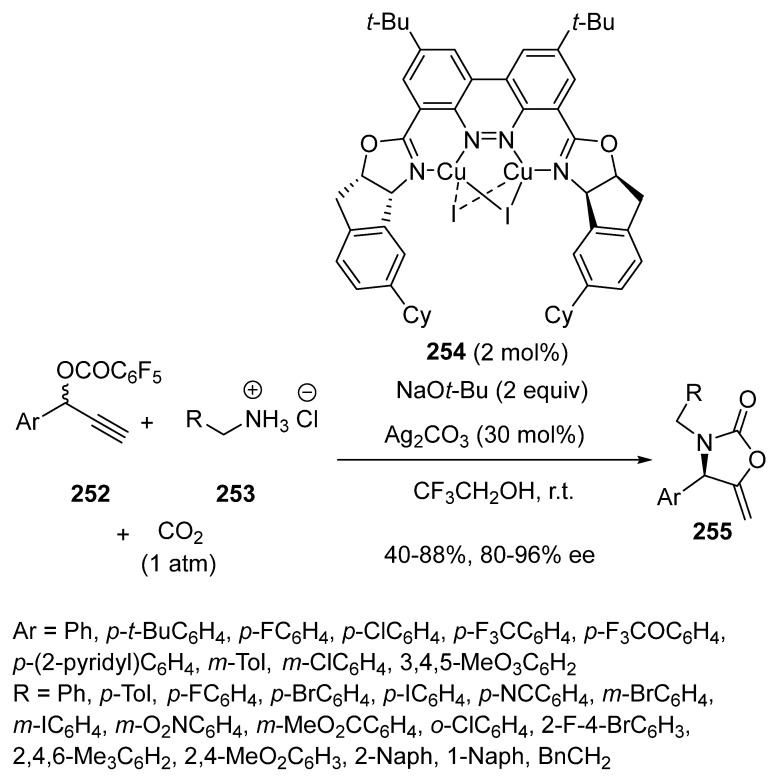
Three-component domino propargylic amination/carboxylative cyclisation reaction of propargylic esters, alkyl amine hydrochlorides and CO_2_ [107].

Chiral 2-oxazolidinones are important molecules in medicinal chemistry and are also of great significance in asymmetric synthesis by their use as chiral ligands or auxiliaries, making their synthesis challenging. In 2024, Wang and Cai developed asymmetric domino propargylic amination/carboxylative cyclisation reactions of aromatic propargylic esters **252** with alkyl amine hydrochlorides **253** and CO_2_ (Scheme 92) [107]. These novel strategies were promoted by 2 mol% of dinuclear copper catalyst **254** derived from a chiral 2-aminoindanol ligand to afford at room temperature under an ambient pressure of CO_2_ a wide variety of chiral 2-oxazolidinones **255** featuring an exocyclic methylene moiety with moderate to high yields (40–88%) and uniformly high ee values (80–96% ee). The methodology tolerated aromatic propargyl carbonates bearing either electron-donating or electron-withdrawing substituents at any position of the phenyl group while the reaction of a cycloalkyl-substituted propargylic carbonate only provided a trace amount of the corresponding product. Various hydrochloride salts of alkyl amines were compatible, such as benzylamines bearing either electronic donating or withdrawing groups in *para-*, *meta*-, *or ortho-*positions of the phenyl ring. The lowest results (40% yield, 80% ee) were observed in the reaction of a primary amine hydrochloride bearing a long alkyl chain (R = CH_2_Bn).

**Scheme 93:**
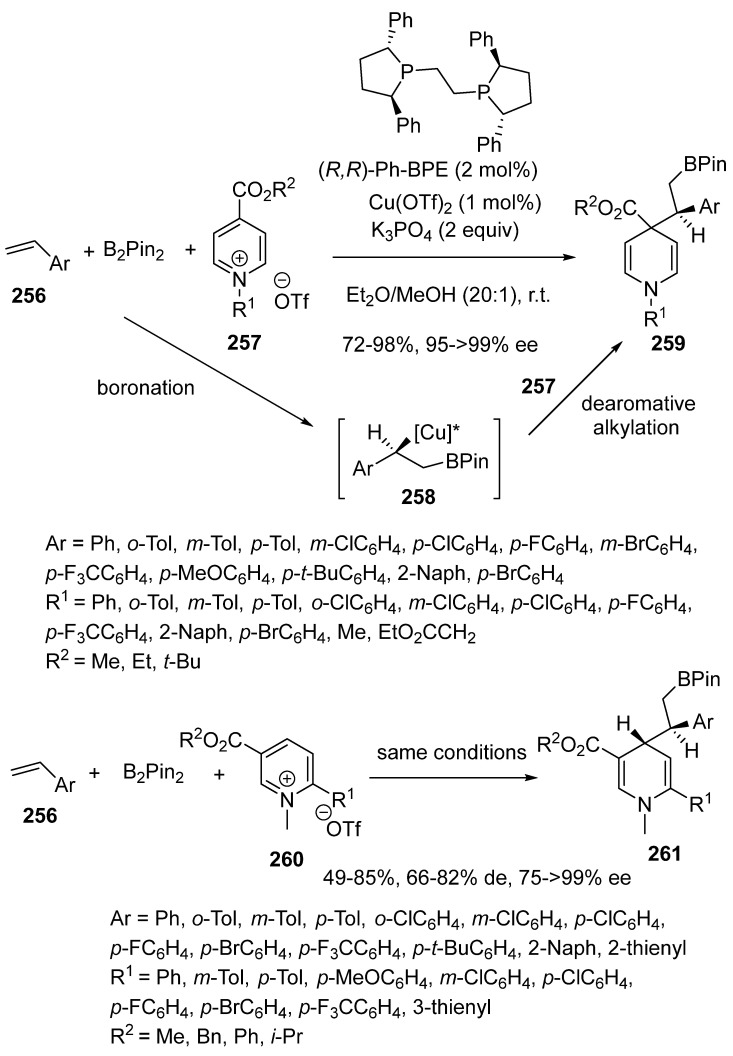
Three-component domino boronation/dearomative alkylation reactions of aromatic alkenes, B_2_Pin_2_ and 4-(alkoxycarbonyl)pyridinium salts/3-(alkoxycarbonyl)pyridinium salts [108].

1,4-Dihydropyridines constitute the skeletons of many drugs and, consequently, their synthesis especially asymmetric is a challenge. In 2025, Zhang et al. developed a novel methodology to prepare these important chiral molecules, which was based on an enantioselective three-component copper-catalysed borylative difunctionalisation of aromatic alkenes 256 with B_2_Pin_2_ and 4-(alkoxycarbonyl)pyridinium salts 257 (Scheme 93) [108]. The optimal conditions involved the use at room temperature of only 1 mol% of Cu(OTf)_2_ associated to 2 mol% of chiral biphenyl ligand (*R*,*R*)-Ph-BPE in a 20:1 mixture of diethyl ether and methanol as combined solvent. The reaction evolved through the nucleophilic addition of chiral borylalkyl copper 258 in situ generated from the addition of B_2_Pin_2_ to the alkene in the presence of the copper catalyst to the pyridinium salt, which provided through dearomatisation of the latter a range of desired chiral boroalkyl-substituted 1,4-dihydropyridines 259 with good to quantitative yields (72–98%) and remarkable enantioselectivities (95- > 99% ee). A diversity of styrenes bearing either electron-withdrawing or electron-donating substituents on the phenyl group were tolerated, all providing products with good to excellent yields and excellent enantioselectivities. Moreover, 2-vinylnaphthalene (Ar = 2-Naph) reacted to give the corresponding product with 77% yield and 99% ee. A range of 4-(alkoxycarbonyl)pyridiniums 257 with electronically different substituents on the phenyl ring were also evaluated, all giving excellent results which showed that the position of these substituents affected only the yield but not the enantioselectivity. To further expand the substrate scope, the authors studied the three-component reaction between styrenes 256, B_2_Pin_2_ and 3-(alkoxycarbonyl)pyridinium salts 260 as the electrophilic partner instead of 4-(alkoxycarbonyl)pyridinium salts 257 (Scheme 93). Under the same reaction conditions, the process afforded in this case chiral 1,4-dihydropyridine products 261 with both moderate to high yields (49–85%) and diastereoselectivities (66–82% de) combined with good to excellent ee values (75- > 99% ee). Styrenes bearing either electron-donating groups or electron-withdrawing groups were compatible, leading to products with remarkable enantioselectivities (97–99% ee) except for that with a chlorine atom at the *ortho* position (Ar = 2-ClC_6_H_4_), which reacted with a lower ee value (75% ee). A variety of 3-(alkoxycarbonyl)pyridinium salts were also demonstrated to be tolerated. In addition to the advantage of employing a low catalyst loading, these novel methodologies allowed an easy access to biologically interesting chiral dihydropyridines with exceptional levels of enantioselectivity, which could be further functionalised into diverse molecules through the transformation of the boron group.

**Scheme 94:**
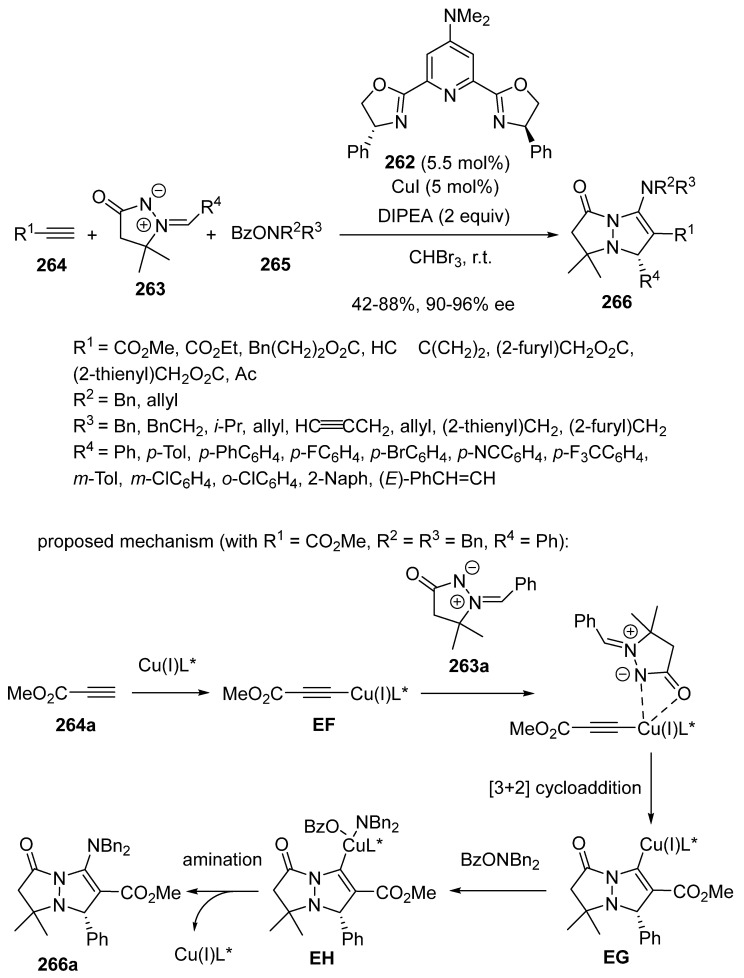
Three-component domino [3 + 2] cycloaddition/amination reaction of azomethine imines, alkynes, and *O*-benzoylhydroxylamines [109].

Chiral Pybox ligand 262 (5.5 mol%) was associated with CuI (5 mol%) by Li et al. in 2025 to promote an asymmetric [3 + 2] cycloaddition/amination domino reaction between azomethine imines **263**, terminal alkynes **264** and *O*-benzoylhydroxylamines **265** (Scheme 94) [109]. The process was performed at room temperature in the presence of DIPEA as a base and CHBr_3_ as a solvent, leading to a wide variety of chiral amino-substituted bicyclic pyrazolines **266** with moderate to high yields (42–88%) and uniformly high enantioselectivities (90–96% ee). The catalyst system tolerated several propiolates bearing alkyl, (hetero)aryl, and alkynyl groups, which afforded the corresponding products with high ee values. An acetylenic ketone (R^1^ = Ac) also reacted smoothly to give the desired pyrazoline with 42% yield and 96% ee. Concerning the *O*-benzoylhydroxylamine partner, *O*-benzoylhydroxylamine, amines bearing *N*-benzyl-*N*-isopropyl substituents, and allyl-, propargyl-, and heteroaryl-substituted hydroxylamines were all compatible with high enantioselectivities. Furthermore, phenyl-substituted azomethine imines exhibiting various types of substituents at the *para*-, *meta*- or *ortho*-positions reacted smoothly with 90–94% ee. In addition to aryl-substituted azomethine imines, an alkenyl-containing azomethine imine (R^4^ = (*E*)-PhCH = CH) produced the desired product with 95% ee albeit combined with a low yield (32%). A mechanism is depicted in Scheme 94, which began with the reaction between CuI, the PyBox ligand, and the terminal alkyne to provide chiral Cu(I) intermediate EF. Subsequently, enantioselective [3 + 2] cycloaddition of the latter with the azomethine imine produced copper(I) intermediate EG. Then, amination of complex EG with the *O*-benzoylhydroxylamine led to the product along with the regenerated active copper(I) species. It must be noted that this work represented the first direct access to chiral fully substituted pyrazolines from readily available terminal alkynes through a domino process.

## 4. Conclusions

This review updates the field of enantioselective domino reactions promoted by green chiral copper catalysts, covering the literature since 2017. These complexes are derived from a diversity of chiral ligands, including mostly bisoxazolines and biphosphines along with monophosphines, *N-*heterocyclic carbenes, proline derivatives, phosphoric acids, phosphoramidates, and different types of *N*,*N*-ligands. The review shows that under catalysis with this cheap and non-toxic metal, an impressive diversity of enantioselective one-, two-, three-, and even four-component domino processes have been developed in the last decade. These economic one-pot processes have allowed the synthesis of extremely complex and densely functionalised acyclic as well as cyclic chiral products under green reaction conditions. Remarkable enantioselectivities have been disclosed for asymmetric one- and two-component reactions, such as Michael-type-initiated reactions, Kinugasa-initiated reactions, borylcupration-initiated reactions, reactions based on intramolecular cyclisation, reactions initiated by decarboxylative propargylation, Mannich-initiated reactions, arylation-initiated reactions, domino yne-allylic substitution/cyclisation reactions, along with miscellaneous reactions. Furthermore, three- and even four-component reactions, including Michael-initiated reactions, photoredox-catalysed reactions, reactions initiated by hydrocupration, along with other reactions also provided excellent enantioselectivities. Undoubtedly, the discovery of novel reactions will continue in the next years on the basis of new chiral environmentally benign copper catalyst systems. In addition, the lower toxicity of copper combined with the high complexity of the chiral molecules synthesised constitute a real hope toward applications in medicinal chemistry.

## Data Availability

Data are contained within the article.

## Abbreviations

Ad	adamantyl
acac	acetylacetone
Ar	aryl
BAr^F^	tetrakis(3,5-bis(trifluoromethyl)phenyl)borate
BINOL	1,1′-bi-2-naphthol
BLED	blue light emitting diode
Bn	benzyl
Boc	*tert*-butoxycarbonyl
BPE	1,2-bis(2-pyridyl)ethane
Bs	benzenesulfonyl
BTM	benzotetramizole
Bz	benzoyl
Cbz	benzyloxycarbonyl
cod	cyclooctadiene
Cy	cyclohexyl
DABCO	1,4-diazabicyclo [2.2.2]octane
dan	1,8-diamino-naphthalatoborane
DCE	dichloroethane
de	diastereomeric excess
DIPEA	diisopropylethylamine
DTBP	ditert-butylpyridine
DTBM	ditertbutylmethoxy
ee	enantiomeric excess
EWG	electron-withdrawing group
Fac	hexafluoroacetylacetonate
FOXAP	ferrocenyloxazolinylphosphine
GDME	ethylene glycol dimethyl ether
Hex	hexyl
L	ligand
LED	light emitting diode
LHMDS	lithium hexamethyldisilazide
MBS	*p-*methoxybenzenesulfonyl
Mes	mesityl
Ms	mesyl
MS	molecular sieves
MTBE	methyl *tert*-butyl ether
Naph	naphthyl
NHC	*N-*heterocyclic carbene
NFSI	*N*-fluorobenzenesulfonimide
NMP	*N*-methyl-2-pyrrolidone
Ns	nosyl (4-nitrobenzene sulfonyl)
Oct	octyl
Pent	pentyl
PG	protective group
Phth	phthaloyl
Pin	pinacolato
PMB	*para*-methoxybenzyl
PMHS	polymethylhydrosiloxane
PMMS	poly[(3-mercaptopropyl)methylsiloxane]
ppy	2-phenylpyridine
Pybox	2,6-bis(2-oxazolyl)pyridine
r.t.	room temperature
Segphos	5,5′-Bis(diphenylphosphino)-4,4′-bi-1,3-benzodioxole
tAm	*tert-*amyl
TBDPS	*tert*-butyldiphenylsilyl
TBHP	*tert*-butyl hydroperoxide
TBS	*tert*-butyldimethylsilyl
Tc	thiophene carboxylate
TEA	trimethylamine
Tf	trifluoromethanesulfonyl
tfacac	trifluoroacetylacetonate
THF	tetrahydrofuran
TIPS	triisopropylsilyl
TMS	trimethylsilyl
Tol	tolyl
Ts	4-toluenesulfonyl (tosyl)
